# Prevention of type 2 diabetes through prediabetes remission without weight loss

**DOI:** 10.1038/s41591-025-03944-9

**Published:** 2025-09-29

**Authors:** Arvid Sandforth, Elsa Vazquez Arreola, Robert L. Hanson, Nicolai J. Wewer Albrechtsen, Jens Juul Holst, Robert Ahrends, Cristina Coman, Felicia Gerst, Estela Lorza-Gil, Yurong Cheng, Leontine Sandforth, Sarah Katzenstein, Marlene Ganslmeier, Jochen Seissler, Hans Hauner, Nikolaos Perakakis, Robert Wagner, Jürgen Machann, Fritz Schick, Andreas Peter, Rainer Lehmann, Cora Weigert, Jennifer Maurer, Hubert Preissl, Martin Heni, Julia Szendrödi, Stefan Kopf, Michele Solimena, Peter Schwarz, Matthias Blüher, Hans-Ulrich Häring, Martin Hrabé de Angelis, Annette Schürmann, Stefan Kabisch, Knut Mai, Andreas F. H. Pfeiffer, Stefan Bornstein, Michael Stumvoll, Michael Roden, Norbert Stefan, Andreas Fritsche, Andreas L. Birkenfeld, Reiner Jumpertz von Schwartzenberg

**Affiliations:** 1 German Center for Diabetes Research (DZD), Neuherberg, Germany; 2 Internal Medicine IV, Department of Diabetology, Endocrinology and Nephrology, University of Tübingen, Tübingen, Germany; 3 Institute for Diabetes Research and Metabolic Diseases of the Helmholtz Center Munich at the University of Tübingen, Tübingen, Germany; 4 Phoenix Epidemiology and Clinical Research Branch, National Institute of Diabetes and Digestive and Kidney Diseases, Phoenix, AZ USA; 5 Department of Clinical Biochemistry, Copenhagen University Hospital—Bispebjerg and Frederiksberg Hospital, Copenhagen, Denmark; 6 Department of Clinical Medicine, Faculty of Health and Medical Sciences, University of Copenhagen, Copenhagen, Denmark; 7 Copenhagen Center for Translational Research, Copenhagen University Hospital—Bispebjerg and Frederiksberg Hospital, Copenhagen, Denmark; 8 The NovoNordisk Foundation Center for Basic Metabolic Research and the Department of Biomedical Sciences, University of Copenhagen, Copenhagen, Denmark; 9 Department for Analytical Chemistry, University of Vienna, Vienna, Austria; 10 Diabetes Center, Department of Medicine IV, University Hospital Ludwig-Maximilians University Munich, Munich, Germany; 11 Institute of Nutritional Medicine and Health, School of Medicine, Technical University of Munich, Munich, Germany; 12 Department of Internal Medicine III, Technical University Dresden, Dresden, Germany; 13 Paul-Langerhans-Institut Dresden (PLID) of the Helmholtz Center Munich at University Clinic Carl Gustav Carus, Dresden, Germany; 14 Department of Endocrinology and Diabetology, Medical Faculty and University Hospital, Heinrich Heine University Düsseldorf, Düsseldorf, Germany; 15 Institute for Clinical Diabetology, German Diabetes Center, Leibniz Center for Diabetes Research at Heinrich Heine University Düsseldorf, Düsseldorf, Germany; 16 Department of Diagnostic and Interventional Radiology, Section of Experimental Radiology, University Hospital of Tübingen, Tübingen, Germany; 17 Institute for Clinical Chemistry and Pathobiochemistry, Department for Diagnostic Laboratory Medicine, University Hospital of Tübingen, Tübingen, Germany; 18 Department of Pharmacy and Biochemistry, Institute of Pharmaceutical Sciences, and Interfaculty Centre for Pharmacogenomics and Pharma Research, Auf der Morgenstelle 8, University of Tübingen, Tübingen, Germany; 19 Department of Internal Medicine I, Ulm University Hospital, Ulm, Germany; 20 Department of Medicine I and Clinical Chemistry, University Hospital of Heidelberg, Heidelberg, Germany; 21 Chair for Endocrinology and Diabetology at the University of Luebeck, Medizinische Klinik 1, University Hospital Schleswig-Holstein, Lübeck, Germany; 22 Department of Medicine, Endocrinology and Nephrology, Universität Leipzig, Leipzig, Germany; 23 Helmholtz Institute for Metabolic, Obesity and Vascular Research (HI-MAG) of the Helmholtz Zentrum München, University of Leipzig and University Hospital Leipzig, Leipzig, Germany; 24 Institute of Experimental Genetics, IEG Helmholtz Center Munich, Neuherberg, Germany; 25 Chair of Experimental Genetics, School of Life Sciences Weihenstephan, Technical University of Munich, Munich, Germany; 26 Department of Human Nutrition, German Institute of Human Nutrition Potsdam-Rehbrücke, Nuthetal, Germany; 27 Department of Endocrinology and Metabolism, Charité—Universitätsmedizin Berlin, Corporate Member of Freie Universität Berlin, Humboldt-Universität zu Berlinand Berlin Institute of Health, Berlin, Germany; 28 Diabetes & Obesity Theme, School of Cardiovascular and Metabolic Medicine & Sciences, Faculty of Life Sciences & Medicine, Kings College London, Denmark Hill Campus, The James Black Centre, London, UK; 29 Cluster of Excellence EXC 2124 ‘Controlling Microbes to Fight Infections’ (CMFI), University of Tübingen, Tübingen, Germany; 30 M3 Research Center for Malignome, Metabolome and Microbiome, Faculty of Medicine, University of Tübingen, Tübingen, Germany

**Keywords:** Metabolic syndrome, Lifestyle modification, Preventive medicine, Type 2 diabetes, Obesity

## Abstract

Clinical practice guidelines recommend defined weight loss goals for the prevention of type 2 diabetes (T2D) in those individuals with increased risk, such as prediabetes. However, achieving prediabetes remission, that is, reaching normal glucose regulation according to American Diabetes Association criteria, is more efficient in preventing T2D than solely reaching weight loss goals. Here we present a post hoc analysis of the large, multicenter, randomized, controlled Prediabetes Lifestyle Intervention Study (PLIS), demonstrating that prediabetes remission is achievable without weight loss or even weight gain, and that it also protects against incident T2D. The underlying mechanisms include improved insulin sensitivity, β-cell function and increments in β-cell-GLP-1 sensitivity. Weight gain was similar in those achieving prediabetes remission (responders) compared with nonresponders; however, adipose tissue was differentially redistributed in responders and nonresponders when compared against each other—while nonresponders increased visceral adipose tissue mass, responders increased adipose tissue in subcutaneous depots. The findings were reproduced in the US Diabetes Prevention Program. These data uncover essential pathways for prediabetes remission without weight loss and emphasize the need to include glycemic targets in current clinical practice guidelines to improve T2D prevention.

## Abstract (web summary)

A post hoc analysis of a multicentre, randomised trial showed that prediabetes remission is possible without total weight loss—providing weight is distributed to subcutaneous deposits as opposed to visceral ones.

## Main

Globally, more than 460 million people live with type 2 diabetes (T2D) and many go through comorbidities such as neuropathy, chronic kidney disease or cardiovascular (CV) disease, making T2D one of the top 10 leading causes of death worldwide. Indeed, the increase in global mortality burden until 2050 is projected to be mostly due to neoplasms, T2D and kidney diseases^1^. Moreover, the current data suggest that T2D incidence will continue to rise, with most cases attributable to diet quality^2^. Additionally, the highest total number of affected people live in middle-income and low-income countries, where current guideline-based treatments are not as easily available as in high-income countries. Therefore, prevention must still be regarded as a major pillar in achieving the WHO’s global noncommunicable disease goals and in equitably reducing the major burden of T2D^3^. Prediabetes is the most prominent risk factor of T2D with a yearly progression rate of 5–10% and a lifetime progression risk of 74%^4,5^, and an independent risk factor for vascular diseases, cancer and neurodegenerative diseases^4,6,7^.

According to the American Diabetes Association (ADA), prediabetes is diagnosed when glucose regulation is impaired and/or HbA1c elevated while criteria of T2D are not met^8,9^. The U.S. Diabetes Prevention Program (DPP) Outcome Study (DPPOS) has demonstrated that reversal of impaired glucose regulation to normal at least once during a lifestyle intervention (LI) was effective in reducing the risk of T2D and microvascular disease^10,11^. In the Prediabetes Lifestyle Intervention Study (PLIS)^12^, we extended these findings by establishing the concept of prediabetes remission^13,14^. Accordingly, weight-loss-induced remission of prediabetes is reached, once the normal glucose regulation (NGR; that is, fasting glucose < 5.6 mmol l^−1^ (100 mg dl^−1^), 2 h glucose < 7.8 mmol l^−1^ (140 mg dl^−^^1^) and HbA1c < 39 mmol mol^−1^ (5.7%)) is re-established. Weight-loss-induced prediabetes remission (>5% of initial body weight) was explained by improved insulin sensitivity and reduced visceral adipose tissue (VAT) volume. At long-term follow-up, participants reaching weight-loss-induced prediabetes remission had a 73% reduced risk of developing T2D compared to those who only met the weight loss goal (but not prediabetes remission) and also had reduced signs of kidney and small vessel damage^13^, which may be due to reduced glycemic exposure over time as has previously been indicated^10^. A comparable concept to prediabetes remission has successfully been established in people with T2D in the DIRECT trial, where substantial weight loss led to a return to nondiabetic glucose levels in up to 85% of participants, although the risk of redeveloping T2D was high^15,16^. In general, part of weight loss targeted LIs is physical exercise, which has been linked with reduced inflammation^17,18^ and has been shown to improve insulin sensitivity independently of body weight change^19^. In individuals with T2D, it has also been demonstrated that physical exercise can improve glycemia without a substantial weight loss effect^20^. However, neither DIRECT nor DPP reported on the preventive outcomes of patients who did not reduce body weight but achieved remission.

Here, we provide evidence that non-weight-loss-induced remission of prediabetes protects from T2D development for up to 10 years after the LI started, and that it is characterized by increased subcutaneous adipose tissue (SCAT) compared to nonremission, where VAT increases. Moreover, we show that non-weight-loss-induced remission of prediabetes is mediated by an improvement in insulin sensitivity and insulin secretion.

Our data provide new support for the importance of implementing glycemic targets into current treatment guidelines to improve T2D prevention^14^, and show that the sole assessment of weight trajectories without body fat distribution is not adequately informative for the pursuit of treatment success.

## Results

### Study design and anthropometry

We analyzed data from the ongoing multicenter LI study PLIS that recruited individuals with prediabetes in study centers throughout Germany within the framework of the German Center for Diabetes Research (DZD e.V.), where participants received the intervention for 12 months and were followed up for up to 9 years with metabolic phenotyping including oral glucose tolerance tests (OGTTs) for glucose metabolism and whole-body magnetic resonance imaging (MRI) for assessment of body fat distribution before and after the intervention and during follow-up (see Methods for details). Of the 1,105 individuals originally included in PLIS, 234 (21.2%) did not lose or even gained weight during the year of the intervention. Of these, 51 returned to NGR, that is, were designated ‘responders’ (R; 21.8%), while 183 (78.2%) were ‘nonresponders’ (NR). Baseline characteristics for both groups are given in Extended Data Table 1. Overall, there were more women in both groups and R tended to have more women than NR (60.1% in NR versus 74.5% in R, *P* = 0.085). As has previously been reported for prediabetes remission^11,13^, R were younger (median = 54.4 ± 17.6 years) than NR (59.4 ± 15.5 years, *P* = 0.013), had lower fasting and 2 h glucose and slightly higher insulin sensitivity. Intervention intensity was not different between groups (Extended Data Table 1).

**Extended Data Fig. 1:**
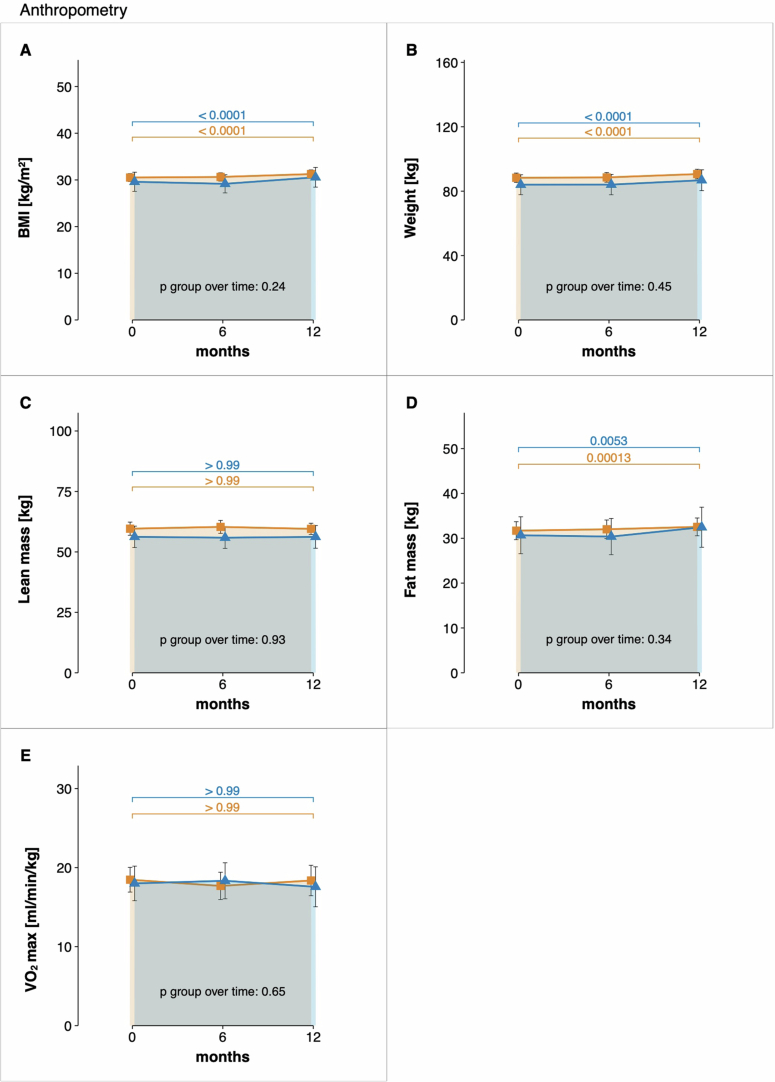
Anthropometric characteristics in PLIS. Anthropometric characteristics in PLIS: BMI, R: n = 51; NR: n = 183 (**a**), body weight, R: n = 49; NR: n = 170 (**b**), lean mass, R: n = 49; NR: n = 170 (**c**), fat mass R: n = 49; NR: n = 171 (**d**) and maximal aerobic capacity, R: n = 17; NR: n = 47 (**e**) during the intervention. Means of raw values are depicted with error bars indicating 95% confidence intervals. P values directly above the x axes indicate change in R versus NR over time (that is, interaction term of group and time) derived from mixed effects models. P values directly above the colored bars indicate the difference between baseline and 12 months for the responder group (blue) and the non-responder group (orange) as post-hoc corrected comparisons derived from the respective mixed effects models. Exact p-values: **a**: R p = 3.03 × 10^−13^; NR p < 2 × 10^−16^; **b**: R p = 6.67 × 10^−13^; NR p < 2 × 10^−16^.

**Extended Data Fig. 2:**
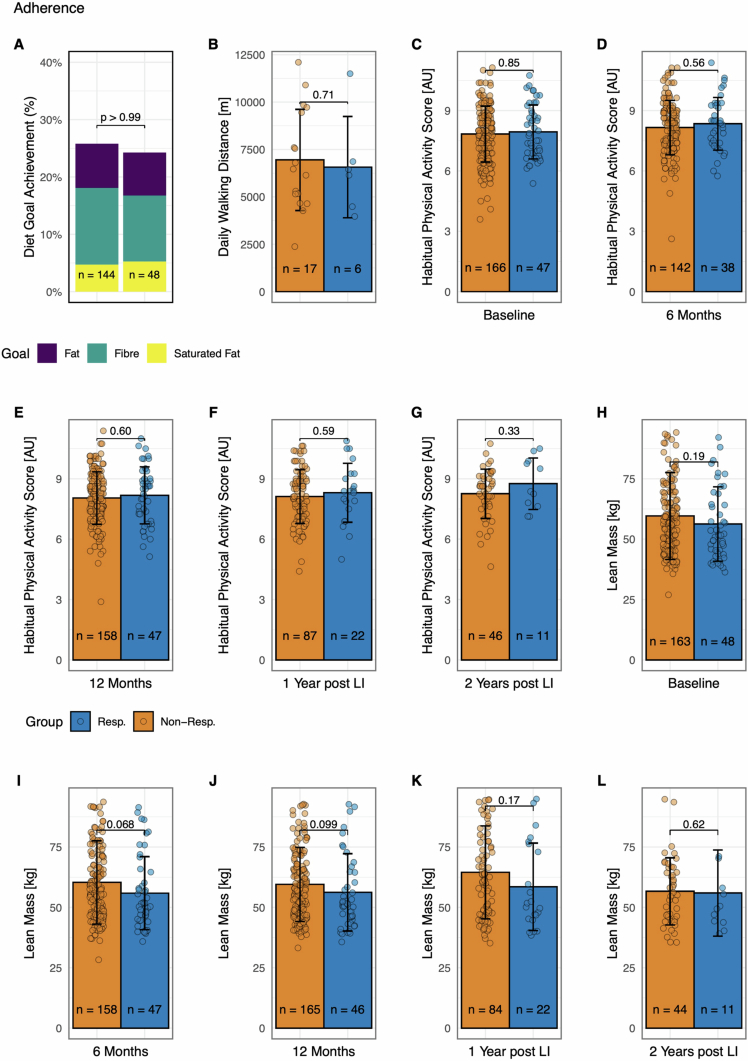
Diet adherence and physical activity. Assessment of markers of diet adherence and physical activity during and after the lifestyle intervention (LI) in PLIS. **a**, Adherence to the diet recommendations assessed during diet counseling sessions. **b**, Daily distance walked inferred by pedometer data after 6 months of LI in a subgroup of individuals (R: n = 6; NR: n = 17). **c**–**g**, The habitual physical activity score during LI and at follow-up. **h**–**l**, Lean body mass during LI and at follow-up. Chi-squared test was used for **a**, all other p values are derived from two-sided Wilcoxon rank sum tests. Means of raw values are depicted and error bars indicate s.d. in **b**–**l**. N for each panel is depicted in the respective group bars.

At first, we investigated whether weight trajectories during the 1-year intervention period were different between R and NR. However, BMI increased similarly in both groups (29.6 ± 2.1 kg m^−2^ to 30.6 ± 2.1 kg m^−2^ in R versus 30.5 ± 0.8 kg m^−2^ to 31.3 ± 0.9 kg m^−2^ in NR, *P* group over time = 0.24; Extended Data Fig. 1a). This was similar for body weight (84.0 ± 6.2 kg to 86.8 ± 6.5 kg in R versus 88.4 ± 2.9 kg to 90.6 ± 2.9 kg in NR, *P* group over time = 0.45), lean mass (56.2 ± 4.4 kg to 56.2 ± 4.7 kg in R versus 59.6 ± 2.7 kg to 59.5 ± 2.3 kg in NR, *P* group over time = 0.93; Extended Data Fig. 1b,c) and fat mass (30.7 ± 4.1 kg to 32.5 ± 4.5 kg in R versus 31.7 ± 2.0 kg to 32.5 ± 1.9 kg in NR, *P* group over time = 0.34; Extended Data Fig. 1d). Maximal aerobic capacity did not differ between groups (18 ± 2.2 ml min kg^−1^ to 17.6 ± 2.5 ml min kg^−1^ in R versus 18.5 ± 1.6 ml min kg^−1^ to 18.4 ± 1.9 ml min kg^−1^ in NR, *P* group over time = 0.65; Extended Data Fig. 1e). Additionally, adherence to dietary advice based on the evaluation of food diaries was not different between groups, similarly neither habitual physical activity (HPA) score nor daily distance walked (in a subgroup) or lean body mass differed between groups during the intervention and during follow-up, respectively (Extended Data Fig. 2).

Taken together, these data show that prediabetes remission without weight loss compared to nonremission was independent of weight trajectories, overall body composition and physical fitness.

### Glucose and insulin metabolism

**Fig. 1:**
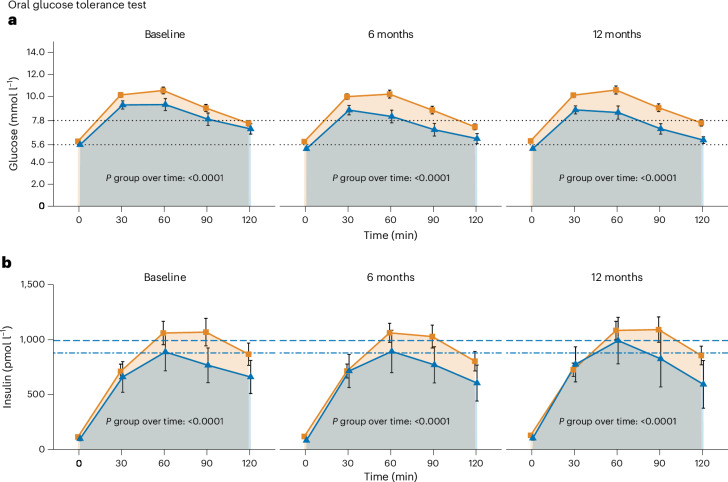
Oral glucose tolerance test. **a**, Time course of glucose (R, *n* = 51; NR, *n* = 183) concentrations during the OGTT over the course of the LI. **b**, Insulin (R, *n* = 51; NR, *n* = 183) concentrations during the OGTT over the course of the LI. Means of raw values are depicted with error bars indicating 95% confidence intervals in all panels. *P* values directly above the *x* axes indicate change in R versus NR over time (that is, interaction term of group and time) derived from mixed effects models. The R (defined by NGR, that is, prediabetes remission, after 12 months of LI) group and the NR (that is, not reaching NGR) group are depicted in blue and orange, respectively. FDR-corrected group × time *P* values are 0.0008 for all comparisons. Dashed lines in **a** represent ADA criteria-based cutoff values for prediabetes for fasting glucose (5.6 mmol l^−1^) and 2 h OGTT glucose (7.8 mmol l^−1^). In **b**, the lower dashed line shows the insulin concentrations at 60 min during the OGTT at baseline and the upper dashed line shows the 60 min peak at 12 months to visualize the change in insulin peak concentrations during the intervention.

Next, we explored the underlying determinants of improved glucose regulation in R versus NR. Glucose concentrations during the OGTT were lower in R and per definition decreased over time in R only (Fig. 1a). Also, consecutive insulin concentrations during the OGTT were higher in NR than in R before, during and after the intervention, but increased in R between 30 and 60 min during the second half of the intervention (Fig. 1b).

**Fig. 2:**
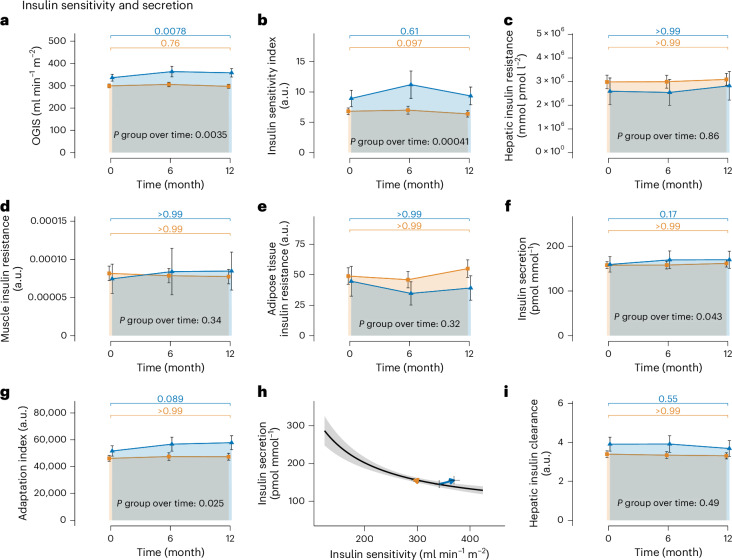
Insulin sensitivity and secretion. **a**, OGIS (R, *n* = 51; NR, *n* = 183). **b**, Matsuda insulin sensitivity index (R, *n* = 51; NR, *n* = 183). **c**, Hepatic insulin resistance (R, *n* = 51; NR, *n* = 183). **d**, Muscle insulin resistance (R, *n* = 51; NR, *n* = 183). **e**, Adipose tissue insulin resistance (R, *n* = 21; NR, *n* = 109). **f**, Insulin secretion (C-peptide/glucose AUC_0–__30 min_—R, *n* = 51; NR, *n* = 183). **g**, Adaptation index (R, *n* = 51; NR, *n* = 183). **h**, The hyperbolic relationship between insulin sensitivity (OGIS) and secretion (C-peptide/glucose AUC_0–__30 min_—R, *n* = 51; NR, *n* = 183) all derived from OGTT at baseline and 12 months. **i**, Hepatic insulin clearance (R, *n* = 51; NR, *n* = 183). The R (defined by NGR, that is, prediabetes remission, after 12 months of LI) group and the NR (that is, not reaching NGR) group are depicted in blue and orange, respectively. Means of raw values are depicted with error bars indicating 95% confidence intervals in **a**–**g**. In **h** and **i**, the base of the arrows depicts medians at baseline and the respective tip after 1 year of LI. Error bars depict s.e.m. *P* values directly above the *x* axes in **a**–**g** and **i** indicate change in R versus NR over time (that is, interaction term of group and time) derived from mixed effects models. *P* values directly above the colored bars indicate the difference between baseline and 12 months for the R and the NR group as post hoc corrected comparisons derived from the respective mixed effects models. FDR-corrected group × time *P* = 0.013 (**a**), *P* = 0.002 (**b**), *P* = 0.89 (**c**), *P* = 0.47 (**d**), *P* = 0.47 (**e**), *P* = 0.10 (**f**), *P* = 0.081 (**g**) and *P* = 0.62 (**i**).

To better understand the increase of insulin concentrations in R, we calculated OGTT-derived indexes for insulin sensitivity, secretion and β-cell function. We found that throughout the LI, insulin sensitivity did not change in NR but increased in R. The oral glucose insulin sensitivity (OGIS) index increased from 336.45 ± 15.41 ml min^−1^ m^−2^ to 358.23 ± 18.88 ml min^−1^ m^−2^ in R, whereas it remained unchanged (299.42 ± 8.67 ml min^−1^ m^−2^ to 297.42 ± 9.09 ml min^−1^ m^−2^) in NR, *P* group over time: 0.0035 (Fig. 2a). Matsuda index of insulin sensitivity revealed the same development with a numeric increase from 8.94 ± 1.35 a.u. to 9.36 ± 1.47 a.u., *P* = 0.096 in R, but did not increase in NR (6.82 ± 0.55 a.u. to 6.39 ± 0.53 a.u. in NR, *P* group over time = 0.00041, Fig. 2b). An index of hepatic insulin resistance did not show differences between groups (Fig. 2c). Also, there was no indication of differences in muscle or adipose tissue insulin resistance (Fig. 2d,e). However, insulin secretion and β-cell function increased in R but not in NR. The C-peptide area under the curve (AUC)_0–__30 min_/glucose AUC_0–__30 min_, an index of insulin secretion, increased from 159.5 ± 17.13 pmol mmol^−1^ to 169.96 ± 19.3 pmol mmol^−1^ in R, but remained constant (157.89 ± 7.81 pmol mmol^−1^ to 161.81 ± 8.15 pmol mmol^−1^) in NR (*P* group over time = 0.043; Fig. 2f). The Adaptation Index, a marker of β-cell function, increased from 5.16 × 10^4^ ± 3,833.80 a.u. to 5.78 × 10^4^ ± 5,145.06 a.u. in R and remained unchanged (4.61 × 10^4^ ± 2,192.89 a.u. to 4.74 × 10^4^ ± 2,554.91 a.u.) in NR (*P* group over time = 0.025; Fig. 2g). An overview of the changes in the hyperbolic relationship between insulin sensitivity and insulin secretion for the two groups is given in Fig. 2h. The hyperbola shows that while R were able to increase insulin sensitivity and secretion at the same time, NR did not improve insulin sensitivity or insulin secretion. Change of hepatic insulin clearance was not different between groups (3.91 ± 0.36 a.u to 3.68 ± 0.4 a.u in R versus 3.39 ± 0.17 a.u to 3.31 ± 0.16 a.u in NR, *P* group over time = 0.49; Fig. 2i) and, thus, cannot explain the increase in insulin concentrations observed in R. With the increase in insulin secretion and β-cell function alongside improved insulin sensitivity, remission mechanisms without weight loss differ from weight-loss-associated prediabetes remission, where the increase in insulin secretion has not been observed previously^13^.

### Body fat distribution

**Fig. 3:**
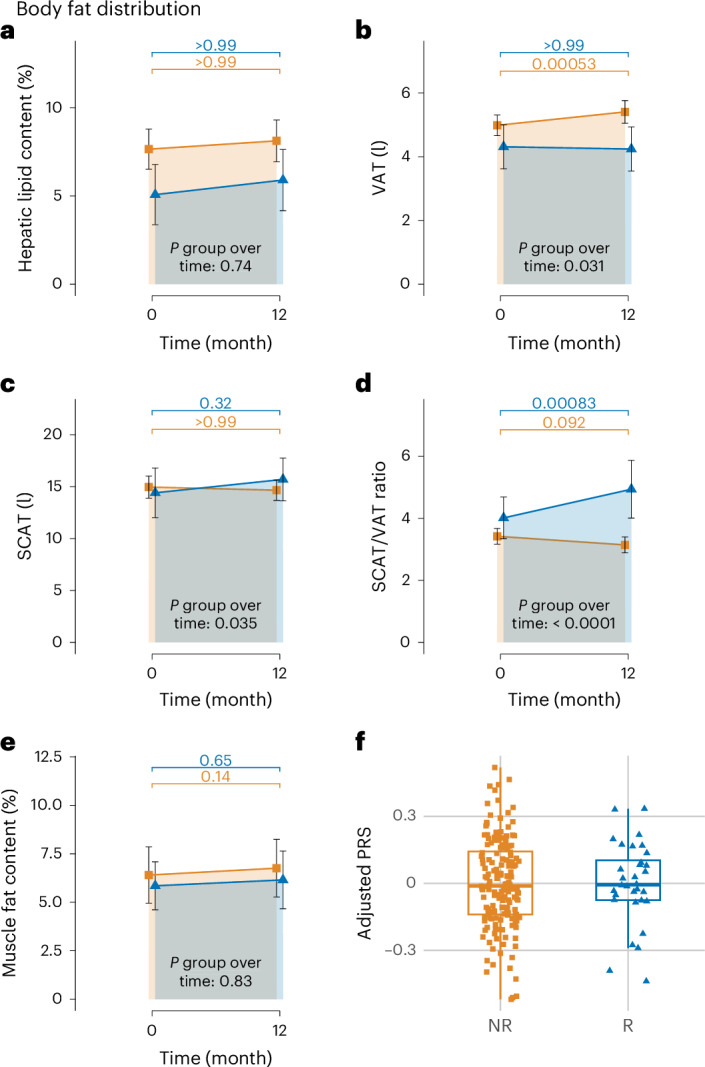
Body fat distribution. **a**, Trajectories of hepatic lipid content as assessed with ^1^H-MRS (R, *n* = 51; NR, *n* = 183). **b**, VAT as assessed with MRI (R, *n* = 51; NR, *n* = 183). **c**, SCAT as assessed with MRI (R, *n* = 51; NR, *n* = 183). **d**, SCAT/VAT ratio (R, *n* = 51; NR, *n* = 183). **e**, Muscle fat content as assessed with MRI (R, *n* = 16; NR, *n* = 27). **f**, Group comparison of the adjusted PRS for VAT volume (R, *n* = 32; NR, *n* = 164). In **a**–**e**, means of raw values are depicted with error bars indicating 95% confidence intervals. In **f**, box plots are centered on medians, boxes extend to 25th and 75th percentiles and whiskers extend to 1.5× IQR (top and bottom). *P* values directly above the *x* axes in **a**–**e** indicate change in R versus NR over time (that is, interaction term of group and time) derived from mixed effects models. *P* values directly above the colored bars indicate the difference between baseline and 12 months for the R and the NR group as post hoc corrected comparisons derived from the respective mixed effects models. The R (defined by NGR, that is, prediabetes remission, after 12 months of LI) group and the NR (that is, not reaching NGR) group are depicted in blue and orange, respectively. FDR-corrected group × time *P* = 0.83 (**a**), *P* = 0.090 (**b**), *P* = 0.092 (**c**), *P* = 0.0008 (**d**) and *P* = 0.89 (**e**). IQR, interquartile range.

Because insulin sensitivity and secretion are linked with ectopic adipose tissue deposition^21–23^, we then determined lipid accumulation in SCAT, VAT and ectopic lipid depots based on the MRI and ^1^H-spectroscopy data. Intrahepatic lipid content (IHL) was slightly higher in NR versus R (Extended Data Table 1) but remained constant in both groups during the LI (5.08 ± 1.71% to 5.9 ± 1.74% in R versus 7.65 ± 1.14% to 8.13 ± 1.18% in NR, *P* group over time = 0.74; Fig. 3a), suggesting no critical contribution of changes in IHL for nonweight-loss remission. However, while VAT did not increase in R despite weight gain, it increased along with weight gain in NR (4.31 ± 0.69 l to 4.24 ± 0.69 l in R versus 4.99 ± 0.32 l to 5.41 ± 0.36 l in NR, *P* group over time = 0.031; Fig. 3b). In contrast, SCAT increased more in R versus NR (14.41 ± 2.39 l to 15.7 ± 2.06 l in R versus 14.97 ± 1.06 l to 14.66 ± 0.98 l in NR, *P* group over time = 0.035) showing that with weight gain, R predominantly stored additional energy in SCAT but NR in VAT (Fig. 3c). This is reflected in a substantial increase in SCAT/VAT ratio in R, whereas the ratio tended to decrease in NR (4.01 ± 0.67 to 4.94 ± 0.93 in R versus 3.42 ± 0.25 to 3.14 ± 0.26 in NR, *P* group over time < 0.0001; Fig. 3d). Change in intramuscular fat was not different between groups (5.85 ± 1.24% to 6.15 ± 1.49% in R versus 6.4 ± 1.45% to 6.76 ± 1.49% in NR, *P* group over time = 0.83; Fig. 3e). These data highlight that lipid deposition during weight gain is most likely a crucial factor for improvements in glucose regulation during non-weight-loss prediabetes remission.

To further investigate whether the genetic background is a key driver of ectopic lipid deposition during weight gain, we calculated a polygenic risk score (PRS) derived from a subset of single-nucleotide polymorphisms (SNPs; Methods) that have been described to be associated with VAT^24^. However, there were no differences in the PRS between R and NR with or without adjustments for covariates (Fig. 3f).

**Fig. 4:**
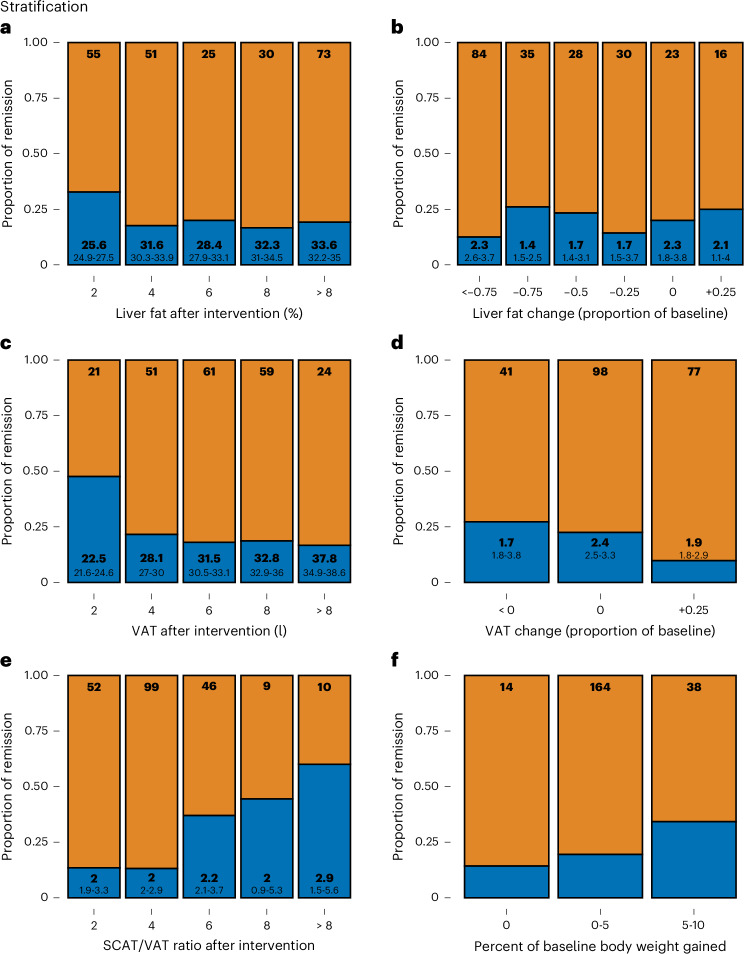
Stratification of prediabetes remission. **a**, Stratification of R (defined by NGR, that is, prediabetes remission, after 12 months of LI, *n* = 51; blue) and NR (that is, not reaching NGR, *n* = 183) by postintervention hepatic lipid content as assessed with ^1^H-MRS. **b**, Change of hepatic lipid content during LI. **c**, Postintervention VAT volume as assessed with MRI. **d**, Change of VAT volume during LI. **e**, SCAT/VAT ratio. **f**, Change of body weight during LI. Top numbers inside the bars indicate stratum size (*n*). Large bottom numbers indicate BMI (**a**,**c**) or percent body weight change (**b**,**d**,**e**). Small bottom numbers indicate 95% CI, respectively.

Similar to previously published data on weight-loss-induced prediabetes remission, there was no association between the change in IHL and the proportion of remission (Fig. 4a,b). However, lower VAT increase or even reductions in VAT were linked with higher remission success (*P* = 0.024; Fig. 4c,d). According to the changes in SCAT and VAT, a higher SCAT/VAT ratio after the LI was associated with higher frequency of remission, again suggesting that the distribution of adipose tissue gained may determine prediabetes remission (*P* < 0.0001; Fig. 4e). Finally, there was a trend toward higher remission rates with weight gain (Fig. 4f).

### Inflammatory markers and adipokines

VAT is associated with increased low-grade inflammation and weight-loss-associated prediabetes remission^13^. Thus, we measured biomarkers of inflammation suggested to contribute to insulin resistance. However, there were no substantial differences in individual trajectories of inflammatory markers between R and NR (Supplementary Tables 1 and 2). Thus, low-grade inflammation, at least at the circulation level, may not explain improved insulin sensitivity during prediabetes remission.

Because hormonal factors derived from adipose tissue are also linked with insulin sensitivity, we measured the adipokines leptin and adiponectin. Leptin levels were not different between groups and did not develop differently (37.25 ± 11.81 ng ml^−1^ to 43.63 ± 11.68 ng ml^−1^ in R versus 40.68 ± 6.24 ng ml^−1^ to 47.26 ± 6.49 ng ml^−1^ in NR). Adiponectin levels, however, were not different at baseline (3096.48 ± 535.75 ng ml^−1^ in R, 2412.30 ± 207.49 ng ml^−1^ in NR, *P* = 0.094), but were substantially higher after the intervention in R versus NR (3371.57 ± 596.55 ng ml^−1^ in R, 2337.20 ± 217.24 ng ml^−1^ in NR, *P* = 0.0040, *P* group over time = 0.088; Extended Data Fig. 2), suggesting that increases in SCAT may chaperone improved insulin sensitivity by increased adiponectin secretion, at least in part.

### Very-low-density lipoprotein (VLDL) palmitate, incretins and glucagon

**Extended Data Fig. 3:**
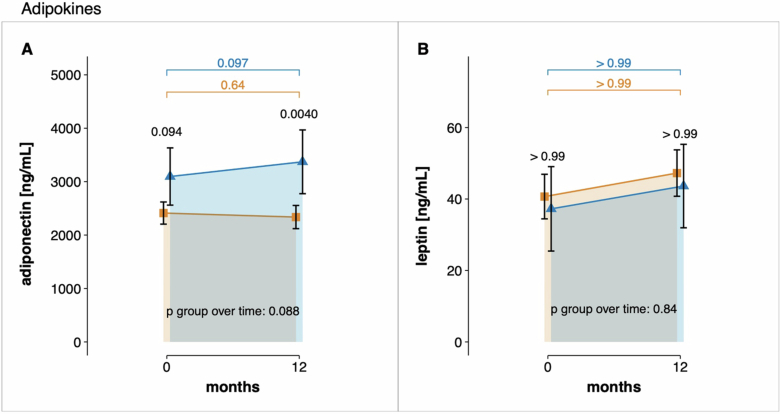
Adiponectin and leptin concentrations during the lifestyle intervention. Concentrations of circulating adiponectin, R: n = 43; NR: n = 169 (**a**) and R: n = 43; NR: n = 149 (**b**) before and after the intervention in PLIS. Means of raw values are depicted with error bars indicating 95% confidence intervals. P values directly above the x axes indicate change in R versus NR over time (that is, interaction term of group and time) derived from mixed effects models. P values directly above the colored bars indicate the difference between baseline and 12 months for the responder group (blue) and the non-responder group (orange) and those directly above the panels comparing the group comparisons at each timepoint, all derived from post-hoc corrected comparisons from the respective mixed effects models.

To further understand the improvement in insulin secretion and β-cell function in R, we assessed lipid content within triglyceride-containing VLDL particles, because a reduction in VLDL1 palmitate has been shown to be associated with improved β-cell secretory capacity during the remission of T2D^25^. Despite higher levels in total and specific lipid species-bound palmitate in NR as well as triglyceride levels, there was no difference in the trajectories during LI between R and NR (Extended Data Fig. 3a–l), suggesting that hepatic palmitate export may not have major impact on the insulin secretion phenotype during prediabetes remission.

**Fig. 5:**
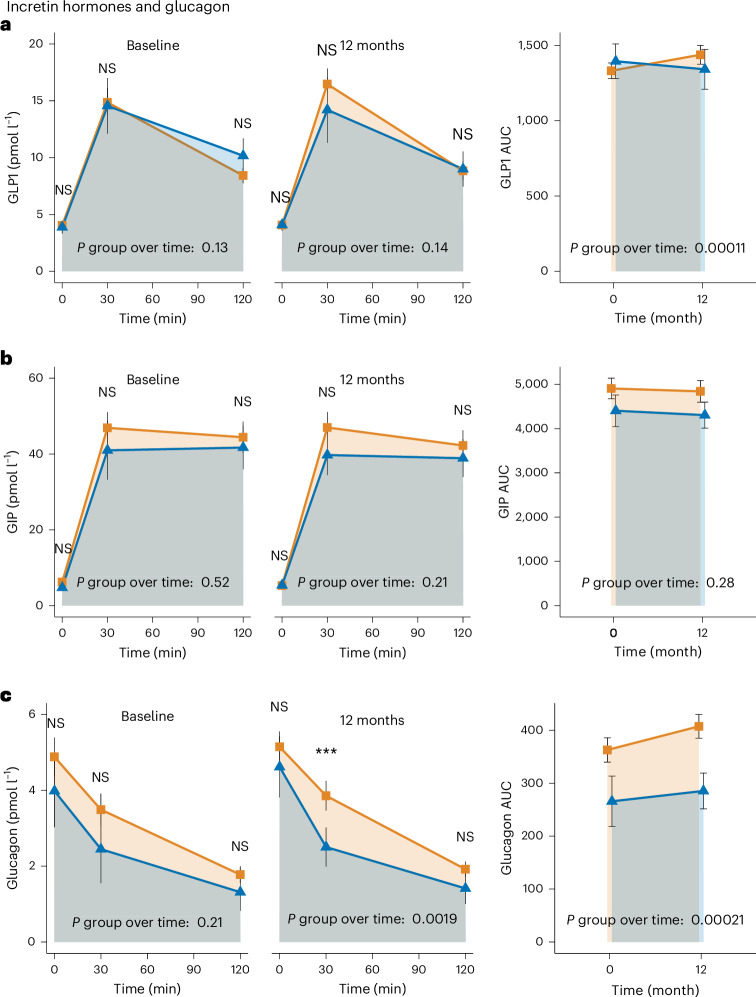
Incretin hormones and glucagon. Incretin hormone levels during the OGTT at baseline and 12 months, *n* = 32 R versus 131 NR. **a**, GLP-1. **b**, GIP. **c**, Glucagon. Means of raw values are depicted with error bars indicating 95% confidence intervals. *P* values directly above the *x* axes indicate change in R (defined by NGR, that i,s prediabetes remission, after 12 months of LI) versus NR (that is, not reaching NGR) over time (that is, interaction term of group and time) derived from mixed effects models. Indicators above the time points indicate between-group difference at the respective time point derived from post hoc comparisons of the respective mixed effects models. ***P < 0.0001; FDR-corrected group × time *P* values: baseline—*P* = 0.29, 12 months—*P* = 0.29, AUC—*P* = 0.0008 (all to **a**); baseline—*P* = 0.63, 12 months—*P* = 0.38, AUC—*P* = 0.45 (all to **b**); baseline—*P* = 0.38, 12 months—*P* = 0.0079, AUC—*P* = 0.0012 (all to **c**). NS, nonsignificant.

We then assessed concentrations of glucagon-like peptide 1 (GLP-1), gastric inhibitory polypeptide (GIP) and glucagon to investigate whether incretin responses during the OGTT or sensitivity (see below) may explain improved insulin secretion in R. As expected, in both groups GLP-1 concentrations increased between 0 and 30 min, and then decreased at 120 min (Fig. 5a). While there was no substantial difference in the trajectories over time between groups (before, *P* = 0.13; after, *P* = 0.14), the GLP-1_AUC_ substantially increased in NR (who maintained that their glucose levels in the prediabetic range or advanced to overt T2D) but remained stable in R despite reduction of glucose levels to normal (*P* = 0.78; Fig. 5a).

GIP followed similar trajectories during the OGTT but remained elevated at 120 min in both groups (Fig. 5b). Neither group trajectories during the OGTT before or after nor GIP_AUC_ differed between R and NR, rendering GIP unlikely to be involved in improved β-cell function during nonweight-loss prediabetes remission (Fig. 5b).

**Extended Data Fig. 4:**
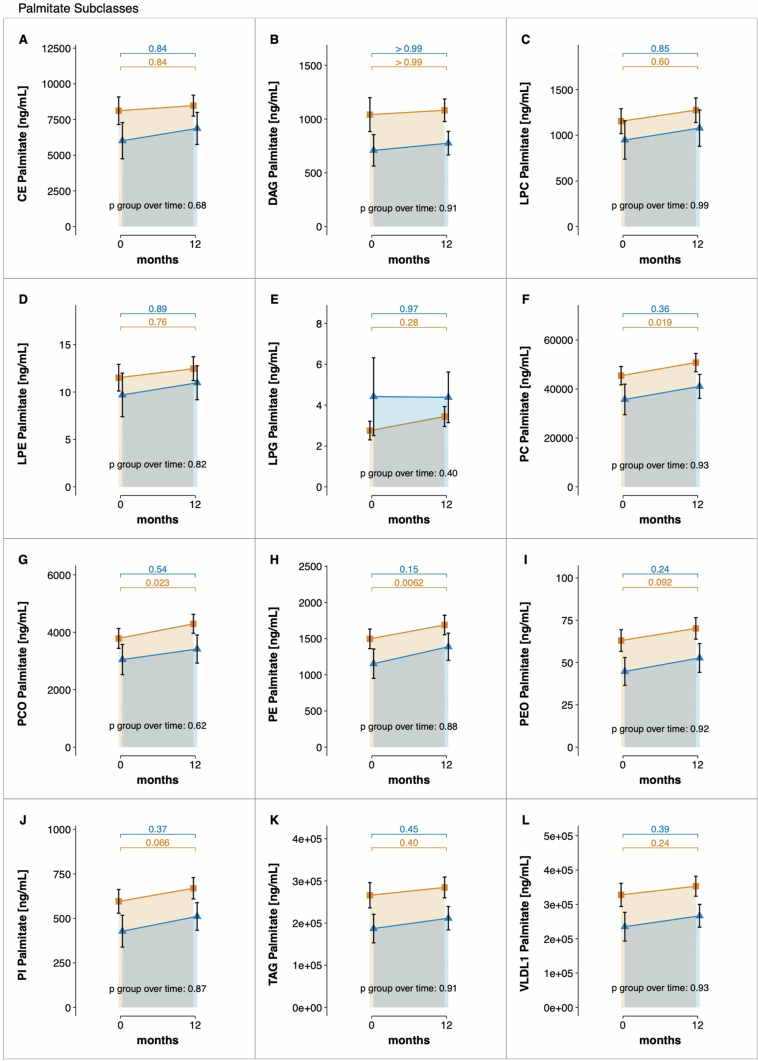
VLDL palmitate subclasses during the livestyle intervention. VLDL palmitate subclasses, R: n=47; NR: n=169 for all panels, in PLIS. Cholesterol ester palmitate (CE, **a**), diacylglycerol palmitate (DAG, **b**), lysophosphatidylcholine palmitate (LPC, **c**), lysophosphatidylethanolamine palmitate (LPE, **d**), lysophosphatidylglycerol palmitate (LPG, **e**), phosphatidylcholine palmitate (PC, **f**), phosphatidylcholine oxidized palmitate (PCO, **g**), phosphatidylethanolamine palmitate (PE, **h**), phosphatidylethanolamine oxidized palmitate (PEO, **i**), phosphatidylinositol palmitate (PI, **j**), triacylglycerol palmitate (TAG, **k**), combined very low-density lipoprotein palmitate (VLDL1, **l**). Means of raw values are depicted with error bars indicating 95% confidence intervals. P values directly above the x axes indicate change in R versus NR over time (that is, interaction term of group and time) derived from mixed effects models. P values directly above the colored bars indicate the difference between baseline and 12 months for the responder group (blue) and the non-responder group (orange) as post-hoc corrected comparisons derived from the respective mixed effects models.

However, glucagon was lower in R and remained unchanged during the LI, whereas in NR glucagon showed an additional increase after the intervention (Fig. 5c). Interestingly, reduction in glucagon concentrations between 0 and 30 min during the OGTT remained stable in NR, but increased substantially in R (Fig. 5c), implying improved glucagonostatic and insulinotropic (considering improved insulin secretion) effects of GLP-1. Therefore, we then calculated the insulin secretion rate based on a previously published algorithm^26–28^ and plotted it against GLP-1 secretion as a measure of β-cell-GLP-1 sensitivity. Indeed, these calculations display that β-cell-GLP-1 sensitivity exhibited marked improvements in R, but not in NR (insulin secretion rate AUC—3,749 ± 401.1 to 4360 ± 382.5 in R and 4,381 ± 164.2 to 4,326 ± 170.1 in NR; GLP-1 AUC—122.1 ± 10.3 to 115.4 ± 13.3 in R and 142.5 ± 5.12 to 152.2 ± 5.96 in NR; *P* group over time = 0.0050; Extended Data Fig. 4).

### Protection from T2D by nonweight-loss-induced prediabetes remission

**Fig. 6:**
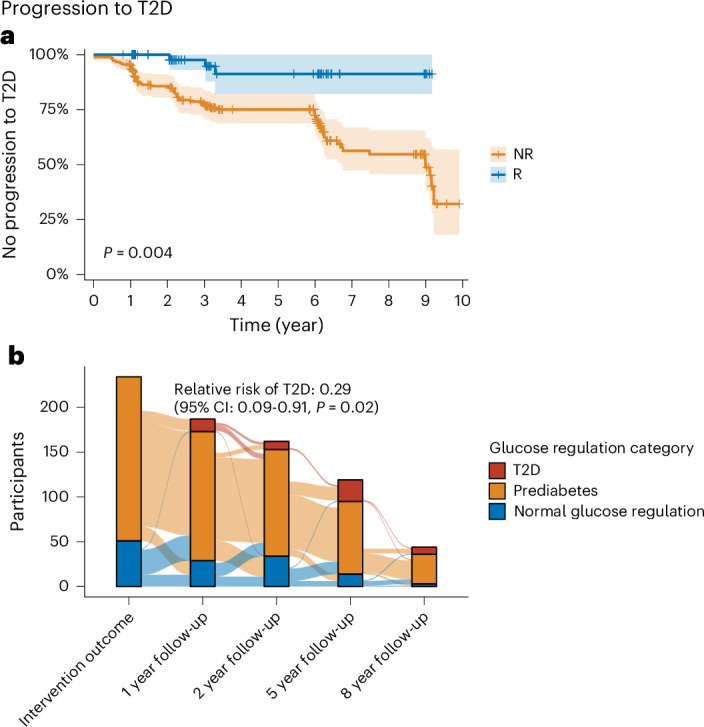
Progression to type 2 diabetes. **a**, Kaplan–Meier curve representing proportion of individuals without T2D over time. Faded colors indicate s.d. from the estimand. *P* value is derived from log-rank tests. **b**, Sankey plot of glycemic categories (that is, T2D, prediabetes and NGR) over time. *P* value is derived from two-sided Fisher’s exact test.

Because of the recent data showing that prediabetes remission with weight loss reduces the risk for future T2D development^13^, we here investigated if remission of prediabetes despite the absence of weight loss or even with weight gain is also protective from future T2D development. Indeed, more NR than R developed T2D leading to a relative risk (RR) reduction of 71% in R over the period of up to 10 years (RR = 0.29, 95% CI = 0.09–0.91, *P* = 0.02; Fig. 6a,b) and thus prediabetes remission without weight loss was comparably protective for T2D development as prediabetes remission with weight loss (73%)^13^.

### Replication of results in the DPP

**Extended Data Fig. 5:**
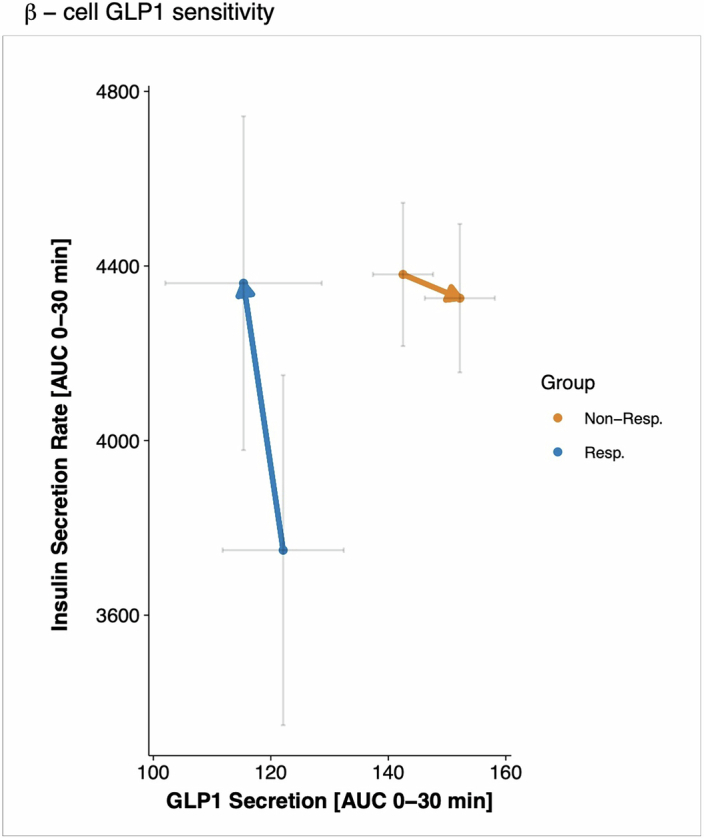
Change in β-cell incretin sensitivity during the lifestyle intervention. β-cell incretin sensitivity expressed as area under the curve of the insulin secretion rate during the first 30 min of the OGTT plotted against the area under the curve of GLP-1 secretion during the first 30 min of the OGTT^83^. The base of the arrows depicts medians at baseline and the respective tip after 1 year of LI. Error bars depict s.e.m. Arrow base indicates coordinate at baseline, arrow tip indicates coordinate at 12 months for the responder group (blue) and the non-responder group (orange), respectively. R: n = 32; NR: n = 131 in PLIS.

**Extended Data Fig. 6:**
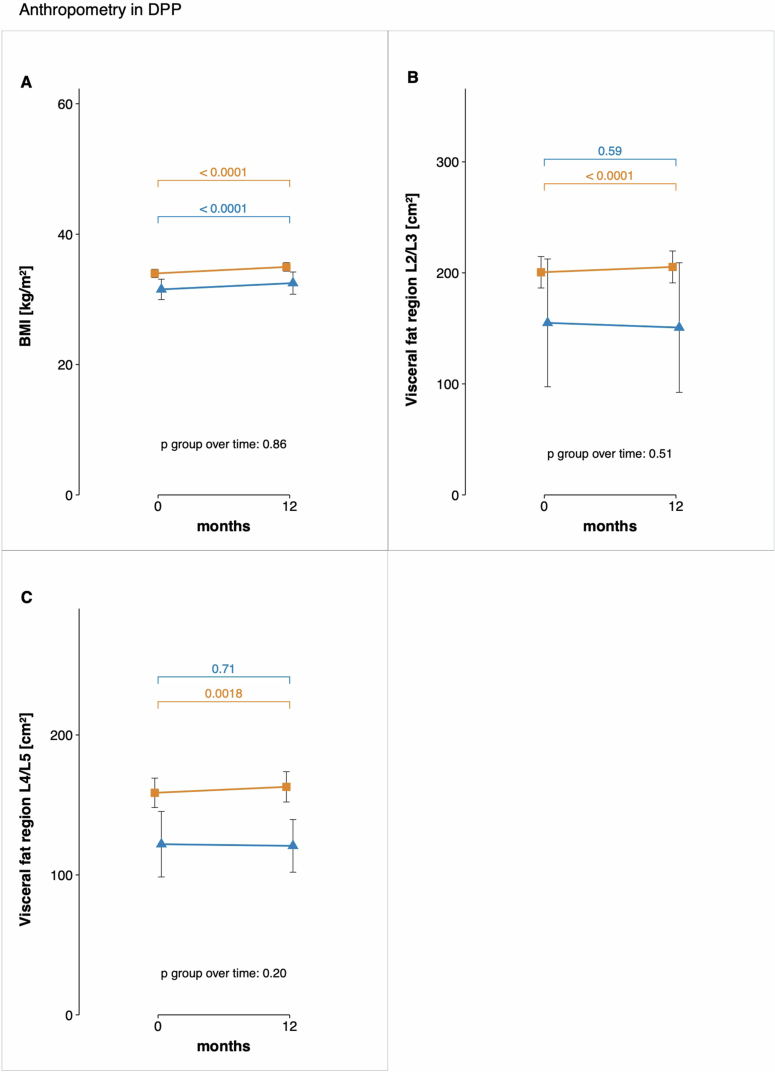
Antropometry in DPP. BMI, R: n = 25; NR: n = 469 (**a**) and visceral adipose tissue trajectories, R: n = 8; NR: n = 160 (**b**, **c**) in individuals participating in DPP. Means of raw values are depicted with error bars indicating 95% confidence intervals. P values directly above the x axes indicate change in R versus NR over time (that is interaction term of group and time) derived from mixed effects models. P values directly above the colored bars indicate the difference between baseline and 12 months for the responder group (blue) and the non-responder group (orange) as post-hoc corrected comparisons derived from the respective mixed effects models. Exact p-values: **a**: R, p = 2.13 × 10^−6^; NR, p = 1 × 10^−8^; **b**: NR, p = 5.85 × 10^−6^.

To exclude any potential effect that may be specific to our cohort and to enhance the generalizability of the results, we validated our findings in repository data from DPP. We also identified 494 individuals who did not lose or even gain body weight after 12 months of the intervention. Baseline characteristics are given in Supplementary Table 3. Similar to PLIS, R and NR increased BMI comparably, and NR increased VAT while R did not (Extended Data Fig. 5a–c). Additionally, R showed improved insulin sensitivity as well as insulin secretion (Extended Data Fig. 6a–c), even after adjusting for insulin sensitivity. Finally, individuals with prediabetes remission in DPP at 1 year without weight loss also had an RR reduction for future T2D of 73% (RR = 0.27, 95% CI: 0.11, 0.66, *P* < 0.0001) during a median follow-up time of 4.11 (IQR: 1.44, 13.48) years. These data reproduce the findings that prediabetes remission without weight loss is characterized by improved insulin sensitivity as well as improved insulin secretion and that excess lipid deposition into VAT may preclude persons from nonweight-loss prediabetes remission.

### Validation using different cutoff values for prediabetes remission

**Extended Data Fig. 7:**
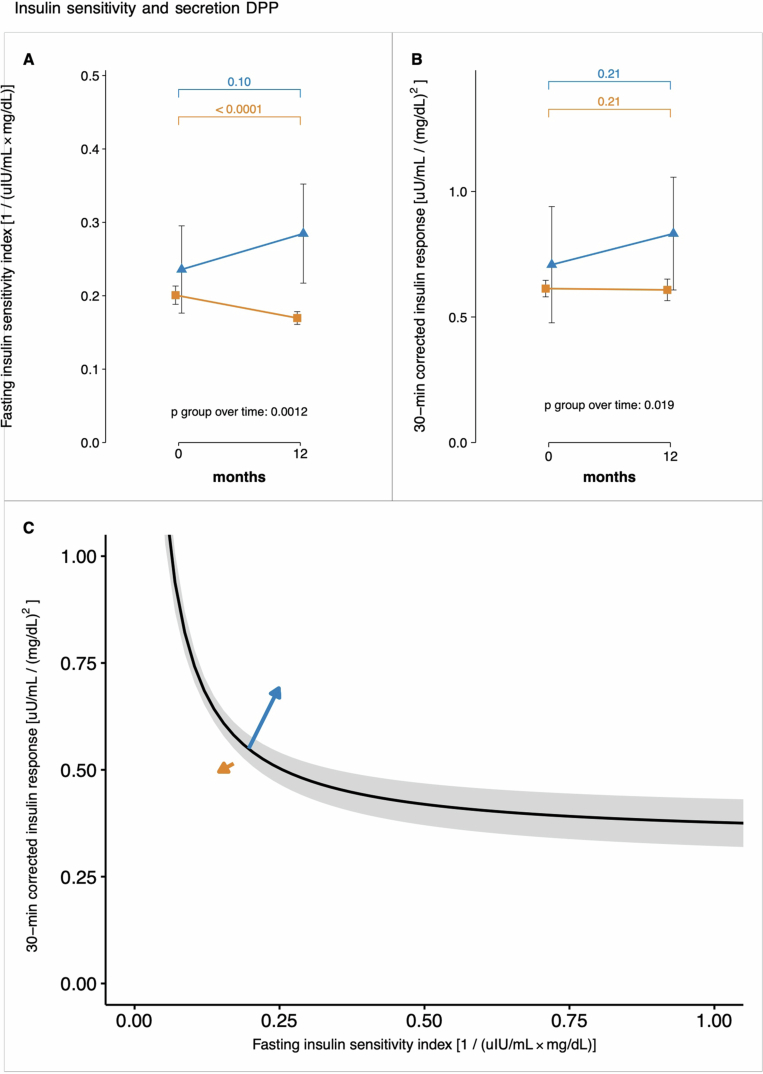
Insulin sensitivity and secretion DPP. Indexes of insulin sensitivity (**a**) and insulin secretion (**b**) in DPP as described in the methods. **c**, The hyperbolic relationship between insulin sensitivity and secretion. Arrow base indicates coordinate at baseline, arrow tip indicates coordinate at 12 months for the R group (blue) and the NR group (orange), respectively. In panel C, means of raw values are depicted with error bars indicating 95% confidence intervals. The base of the arrows depict medians at baseline and the respective tip after 1 year of LI. P values directly above the x axes indicate change in R versus NR over time (that is, interaction term of group and time) derived from mixed effects models. P values directly above the colored bars indicate the difference between baseline and 12 months for the responder group (blue) and the non-responder group (orange) as post-hoc corrected comparisons derived from the respective mixed effects models. R: n = 25; NR: n = 469 for each panel. Exact p-values: **a**: NR, p = 2.48 × 10^−7^.

Moreover, recent data indicate that the 1 h glucose OGTT value may better predict the development of T2D than the 2 h OGTT value^29^. We, therefore, performed the same analysis by using the proposed 1 h cutoff of 155 mg dl^−1^ to identify participants as having prediabetes or NGR, but this change in criteria for prediabetes did not change our findings regarding glucose and insulin metabolism during prediabetes remission (Extended Data Fig. 7).

## Discussion

Our data show that remission of prediabetes without weight loss is a genuine and reproducible response to an LI in a substantial proportion of participants (up to 22%) and that the response is highly effective in preventing T2D. Individuals going into remission without losing body weight are characterized by increased SCAT/VAT ratio, indicating that the site of fat deposition during an LI determines the probability of prediabetes remission. Elegant studies have shown that physical exercise reduces glycemia with no or only marginal effects on body weight^20,30^. The studies in refs. ^31,32^ have shown that exercise without a weight loss effect can specifically reduce VAT. Additionally, diet composition has a role in VAT accumulation—diets high in polyunsaturated fatty acids have been shown to protect against VAT gain compared to those rich in saturated fats, even during overfeeding and weight gain^33^. Thus, physical exercise and dietary habits may have contributed to lower glycemia and to the observed SCAT and VAT distribution phenotype.

Moreover, non-weight-loss-associated prediabetes remission was characterized by a combination of higher insulin sensitivity and improved insulin secretion, which contrasts this response from weight-loss-induced prediabetes remission in PLIS and DPP where remission was characterized by improved insulin sensitivity but not secretion and T2D remission in DIRECT, which was accompanied by improved β-cell function but not insulin sensitivity^13,21,34^. Notably, prediabetes remission without weight loss was protective from T2D development with an RR reduction of >70%, and thus to a comparable degree as weight-loss-induced prediabetes remission in the previous study^13^. Taken together, these data show that distinct physiological mechanisms underlie non-weight-loss-induced remission of prediabetes and that it is essential to differentiate between weight-loss and non-weight-loss strata, when investigating the mechanisms that can mediate prediabetes remission. Moreover, our data suggest that weight loss, as recommended in current guidelines, should be complemented by remission to NGR to prevent T2D in those people with prediabetes. This is in line with recent efforts delineating the clinical heterogeneity of obesity in projection for cardiometabolic diseases, specifically when cardiometabolic risk is discordant with the risk that is to be expected when obesity is classically defined solely by the BMI^35^. Furthermore, new statements and definitions of expert commissions for the diagnosis and management of obesity highlight the importance of body fat distribution and function beyond adiposity and underscore the clinical manifestation of organ dysfunction for the diagnosis of clinical obesity^36,37^.

While body weight trajectories were similar between groups, fat accumulation in SCAT was mainly observed in R and accumulation in VAT was observed in NR. The details in ref. ^38^ showed that large amounts of SCAT in combination with little visceral adiposity are associated with high insulin sensitivity during a hyperinsulinemic–euglycemic clamp. Exercise can favorably influence SCAT composition, even without weight loss^39^, and SCAT of regularly exercising individuals is more expandable than that of sedentary people with obesity^40^. The importance of exercise for improving insulin sensitivity has previously been demonstrated^41^; however, lean body mass and VO_2_ max, a marker of maximum aerobic capacity, were not different between R and NR. Nonetheless, we cannot rule out that subtle differences in physical exercise (volume) may have impacted insulin sensitivity. Although we did not observe a difference in the PRS, the genetic basis of unfavorable body fat distribution and T2D risk has previously been reported^42–44^. Interestingly, thiazolidinediones (TZDs), a class of oral glucose-lowering medications that activate the transcription factor, peroxisome proliferator-activated receptor γ, led to a redistribution of fat mass from VAT to SCAT depots. TZDs act as insulin sensitizers at least in part by this mechanism^45,46^. They have also been shown to increase body weight and to improve insulin sensitivity and β-cell function, similar to our observation with nonweight-loss prediabetes remission^45,47^.

This trend toward a favorable adipose tissue composition tracks closely with systemic adiponectin concentrations. Adiponectin is released by adipose tissue and is associated with increased systemic insulin sensitivity^48^. This finding is in line with recent findings of a gradual decrease of adiponectin levels from metabolically healthy lean to metabolically healthy obese to metabolically unhealthy obese persons^49^. Also, in the data presented here, adiponectin levels were higher in R after the intervention, which may contribute to their increased insulin sensitivity.

In R, but not NR, insulin secretion and β-cell function increased during LI. Palmitate, partly a product of hepatic de novo lipogenesis, has a particularly detrimental role in reducing β-cell function, most likely by inducing β-cell dedifferentiation^50^. Hepatic palmitate secretion has been shown to be modifiable by LI and reduced VLDL1 palmitate export from the liver has been shown to be linked with T2D remission in the DIRECT trial as well as remission maintenance after weight-loss-dependent remission^25^. In our study, in the setting of non-weight-loss prediabetes remission, VLDL1 palmitate export did not change between R and NR, implying that hepatic VLDL1 palmitate export may not be responsible for the lack of increase in β-cell function in the NR. However, we did not assess VLDL-production rates, previously demonstrated to be important for T2D remission^25^.

We then assessed the incretin system to explain the improvement in insulin secretion observed in R, but not in NR. Both GLP-1 and GIP enhance glucose-dependent insulin secretion^51^. GLP-1 secretion after glucose ingestion is only minimally reduced in impaired glucose tolerance (IGT) as well as in T2D^52,53^. Exogenous application of GLP-1 in pharmacologically relevant doses largely maintains its effectiveness in IGT as well as in T2D^54^. GIP levels after glucose ingestion are in the normal range in IGT and may be elevated in T2D^53,55^. However, its effectiveness in augmenting insulin secretion is reduced^56^. In our cohort, GLP-1 and GIP time courses during OGTT were not different between groups. GLP-1_AUC_ remained stable in R despite reduced glucose levels and increased insulin levels (Fig. 2). This may reflect improved GLP-1 sensitivity in R, potentially contributing to improved β-cell function by its improved insulinotropic effect that has been shown to be more pronounced during normoglycemic conditions^57^. Indeed, an index of β-cell-GLP-1 sensitivity showed increased sensitivity in R, but not in NR. Because GLP-1 has a glucagonostatic effect^58^, improved GLP-1 sensitivity may be supported by the finding that R showed improved glucagon suppression during the OGTT. However, we cannot exclude that this could also be a consequence of the improvements in glucose regulation restoring the glucosuppressive effect on glucagon that may also be influenced by diet^59^.

Prediabetes remission without weight loss reduced the risk of subsequent T2D by 71% in our study. Previous studies of people with weight-loss-induced prediabetes remission showed similar risk reductions (73%)^13,60^, which is comparable to T2D risk reduction induced by pharmacological interventions^61^. In a post hoc analysis from the PROactive trial, weight gain with pioglitazone, a TZD, which leads to weight gain with a redistribution from VAT to SCAT and improvements in insulin sensitivity^62,63^, was associated with improved CV outcomes (excluding heart failure)^64^. Interestingly, recent data from our group show that prediabetes remission, in contrast to multimodal LIs targeting mainly weight loss, is also associated with reduced CV outcomes^65,66^.

Together, these data indicate that there is a weight-independent component of the glycemic status, which is related to body fat distribution. Indeed, individuals reaching both prediabetes remission and the guideline-recommended weight loss target of 7% are 76% less likely to develop T2D^8^, compared to the group that only met the weight loss target^60^. These data highlight the importance of incorporating glycemic targets into practice guidelines in addition to weight loss targets. Remission of prediabetes is the most effective way to prevent future T2D cases, and our current data indicate that this is partially independent of weight loss. In fact, sustainable weight loss is rarely achievable with >90% of weight loss recidivism and the probability of obtaining normal body weight is very low with less than 1 per 1,000 for men and less than 1 per 600 for women in individuals with severe obesity^67,68^. Therefore, from a clinical perspective, primary treatment goals should focus on achieving metabolic health rather than weight loss alone. Bringing prediabetic hyperglycemia back to normoglycemia seems to be an important contribution and indicator for metabolic health^69^.

This study is limited by the surrogate parameters derived from the OGTT for estimation of insulin sensitivity and secretion/β-cell function. Additionally, we cannot rule out confounders that were not assessed, such as genetic predisposition (beyond the investigated SNPs) and environmental factors. This study is additionally limited by the lack of a priori powering due to the post hoc nature of this analysis. To strengthen the validity of the data, we have therefore included data from the DPPOS cohort that replicate and support our current findings and interpretations. Additionally, the post hoc group stratification may lead to residual confounding effects, that is, the stratification into remission and nonremission leads to baseline differences, for example, in glucose levels, as has also been observed in studies investigating T2D remission mechanisms^70^ and in previous analyses from DPPOS^10,11^, which may affect the likelihood of remission at the individual level. Nonetheless, we show that for individuals who do achieve remission, the risk of future T2D development is substantially reduced—even in the absence of weight loss. This finding underscores the importance of incorporating remission as a core element of modern T2D prevention strategies, regardless of baseline. To minimize confounding of our results by baseline misclassification, we included fasting as well as postchallenge glucose and HbA1c into our definition of remission, whereby test–retest reliability has been shown to be up to 98.6%^71^. While spontaneous remission from prediabetes to normoglycemia might be observable even without LI^72^, our findings are not likely to be influenced by an erroneous classification.

We conclude that the protective effect of LI in individuals with prediabetes is not solely dependent on weight loss, as none of the participants included in the present analysis lost weight. Non-weight-loss prediabetes remission is characterized by a ‘TZD-like’ body fat distribution. Current guideline recommendations involve multimodal LI with a focus on weight loss targets of 5–10%. In light of the current data, we recommend achieving metabolic health by incorporating glycemic targets to reach NGR (prediabetes remission) in addition to weight loss, thereby optimizing T2D risk reduction through a precision prevention approach. Specifically, LI needs to be tailored to the goal of prediabetes remission^73^.

## Methods

### Study design and PLIS cohort

Participants of the risk-stratified, randomized, controlled and multicenter PLIS were recruited between 1 March 2012 and 31 August 2016 to assess potential superiority of an intensified LI compared to conventional DPP-based LI in improving glucose tolerance in people with prediabetes at high risk for T2D development. Sex was determined through self-report. People with prediabetes (impaired fasting glucose, IGT or both according to criteria from the ADA) between the ages of 18 and 75 years were included^8^. Participants with high IHL in addition to either high insulin resistance or low insulin secretion were stratified as high risk. After stratification, randomization of high-risk participants to receive either intensified LI or conventional LI and of low-risk participants to receive either conventional LI or a control intervention was performed. For this analysis, individuals from PLIS who did not experience weight loss over the 12-month period of those of the intensified LI, the conventional LI or the control intervention were included. Participants were classified as either R or NR. R were defined by return to normal fasting plasma glucose (that is, <5.6 mmol l^−1^), normal glucose tolerance (that is, 2 ­h post­load glucose <7.8 mmol l^−1^) and HbA1c less than 39 mmol mol^−1^ after 12 months of all three interventions. NR were defined by fasting plasma glucose, 2 h glucose or HbA1c higher than these thresholds after 12 months of all three interventions. Glucose regulation was assessed in the morning at 8 a.m. during an overnight fast through OGTT with blood sampling at 0, 30, 60, 90 and 120 min using a 75 g glucose load (Accu-Check Dextro O.G.T.; Roche). Glucose concentrations were assessed using the glucose oxidase method in certified laboratories at each clinical study site. All insulin and C-peptide measurements were done at the Tübingen study site on an ADVIA Centaur XP system (Siemens Healthineers). Clinical chemistry parameters, including HbA1c, were assessed at the local routine diagnostic laboratories of the participating centers, and all of these were certified by the German accreditation council (DAkkS).

Volunteers participated in this study for a mean of 5.2 ± 2.8 years (max = 9.9 years). Adherence to the dietary intervention was assessed by evaluation of food diaries through the study counselors during the counseling sessions, where food diaries of 4 consecutive days were evaluated. The dietary goals were reduction of fat intake to <30% of total energy intake, reduction of saturated fat intake to <10% of total energy intake and increase of fiber intake to >15 g per 1,000 kcal total energy intake. Markers of physical activity and fitness were assessed by the VO_2_ max during ergometry, quantification of lean body mass and the HPA. The VO_2_ max was assessed as the maximal oxygen uptake during incremental physical exertion on an ergometer, lean body mass was estimated through bioelectric impedance analysis and the HPA based on the self-report that addressed the following main components: physical activity during work, physical activity during leisure time and sports during leisure time^74^. In a small subgroup of individuals, step counts were extracted to document daily distance walked (R/NR, *n* = 6/17). Further details of the intervention are provided elsewhere^12^. Of these measures, lean body mass and the HPA were continuously assessed. Follow-up is ongoing. Written informed consent was provided by each participant at inclusion. The study protocol is available online (https://www.dzdev.de/fileadmin/DZD/PDF/Papers/Forschung_KlinStud_Ethik_Studienprotokoll_PLIS_1.4.pdf) and approved by the ethics committee of the University Clinic of Tübingen (Tübingen, 55/2012; ClinicalTrials.gov registration: NCT01947595). Further details have been published previously^12^. Financial compensation for participants was granted exceptionally upon request, only if participants lived far away from the study center.

### DPP study validation cohort

Repository data from the U.S. DPP study were analyzed to verify results from PLIS in a different population^75,76^. Participants were recruited from 23 clinical study centers in the United States between 31 July 1996 and 18 May 1999, and randomized to either LI or metformin or placebo groups. For the present analysis, data from LI and placebo group participants were used, who did not lose body weight during the first 12 ­months and had complete data on measurements of BMI, fasting and 30­-min insulin, and fasting, 30 ­min and 120-­min glucose during an OGTT.

Ethics approval for the study was provided by the institutional review board of each participating clinical center. Written informed consent was obtained from all DPP participants. The DPP study protocol is available online at https://repository.niddk.nih.gov/study/38.

### Outcomes

This is a post hoc analysis of a prespecified endpoint in PLIS. Main outcomes are indexes of insulin secretion and sensitivity and distribution of body fat as main determinants of response or nonresponse to an LI.

The specifics of sampling and processing have been published elsewhere^12^. All measurements were taken from distinct participants. The following OGTT-based indexes for insulin sensitivity were calculated: OGIS index^77^, insulin sensitivity index^78^, the adipose tissue insulin resistance index^79^, muscle insulin sensitivity index^80^ and hepatic insulin resistance index^81^. To evaluate insulin secretion, we used C­-peptide AUC divided by glucose AUC during the first 30 min of the OGTT^82^. The Adaptation Index was calculated as (AUC_c­pep0–30_ divided by AUC_gluc0–30_) × OGIS to provide an integrated measure for insulin secretion adapted to underlying insulin resistance^83^. AUC_c­pep0–120_ divided by AUC_ins0–120_ was calculated as a marker of hepatic insulin clearance^84^.

Insulin secretion rate was calculated by fitting C-peptide concentrations at each time point during the OGTT to an established model of C-peptide deconvolution^26–28^. An incremental area under the first 30 min of the curve resulting from this modeling was calculated using the trapezoidal rule^85^. An incremental AUC was obtained from GLP-1 measurements during the OGTT in a similar fashion. These values were plotted against each other to depict β-cell-GLP-1 sensitivity, as previously described^86^. Insulin secretion and sensitivity were assessed from OGTT-based indexes in DPP in a similar fashion. Details have been previously published^75,76^.

The MRI was performed to measure muscle fat, subcutaneous and visceral fat compartments in PLIS. The applied methods are described in detail elsewhere^12,87^. VAT volume was determined from T1-weighted fast spin echo images that were assessed with a slice thickness of 10 mm (ref. ^88^). Segmentation of VAT was done between hip and thoracic diaphragm using an automatic fuzzy c-means algorithm and orthonormal snakes^89^. IHL was quantified in PLIS only. Localized proton magnetic resonance spectroscopy (^1^H­MRS) applying a single­voxel stimulated echo acquisition mode localization technique with short echo time in the posterior part of segment VII was applied for IHL assessment. The ratio of signal integrals of fat (methylene + methyl signal) and total signal (water + fat) was used to determine IHL content, which is expressed in percent. In one center, ^1^H-MRS was not available and IHL content was determined by a chemical-shift selective imaging technique that generates fat and water selective images as described elsewhere^90^. From these, manually determined regions of interest in liver segment 7 performed in fat and water selective images, respectively, were used for IHL-content assessment. To ensure comparability with MRS-guided assessments, IHL content was calculated by fat/(water + fat) × 100, including corrections for relaxation effects. Both techniques have been shown to yield comparable assessments of IHL content^91^. More details have been described previously^92^.

In DPP, VAT measurements were obtained by lumbar spine CT scans at L2, L3, L4 and L5. IHL and SCAT were not quantified in DPP. Only in PLIS, fat-selective MRI sequences were used to measure intramuscular fat fraction of musculus erector spinae and musculus spinalis thoracis. A region of interest avoiding macroscopic fat septae was manually drawn at the Th10/Th11 intervertebral level in a single axial slice^90,92^. Close proximity SCAT was used for reference (that is, 100%) and fat fractions were calculated^12,87^.

#### Assessment of VLDL palmitate

VLDL were isolated from plasma by ultracentrifugation applying an NaCl density gradient (*ρ* = 1.006 g ml^−1^) at 541,000*g* for 60 min at 4 °C on an Optima Max-XP ultracentrifuge (Beckman). Subsequently, 60 µl of the VLDL-containing fraction were used for lipid extraction and shotgun analysis as previously described^93,94^. Lipid extracts were resuspended in IPA/MeOH/CHCl3 (4:2:1; vol/vol/vol) with 7.5 mM ammonium acetate and then infused through the TriVersa NanoMate ion source (Advion Biosciences) into an Exploris 240 (Thermo Fisher Scientific) mass spectrometer. All spectra were imported by LipidXplorer (1.2.8.1) into a MasterScan database. Lipid identification was carried out as described^95^.

### Inflammatory markers, adipokines, incretins and glucagon

Multianalyte profiling on the Luminex-100 system (Bio-Rad Laboratories), with a combination of Bio-Plex Pro Human Immunotherapy Panel 20-Plex, Bio-Plex Pro Huma Inflammation Panel, Bio-Plex Pro Human Cytokine Panel and the 2-Plex Panel for vascular cell adhesion molecule 1 and intercellular adhesion molecule 1, was used to assess concentrations of circulating inflammatory markers in serum. Bio-Plex Multiplex Immunoassays (Bio-Rad Laboratories) are based on the xMAP Technology licensed from Luminex; all measurements were conducted according to the manufacturer’s protocols. A detailed description of the methods is given elsewhere^96^. Serum concentrations of leptin (11-LEPHU-E01; Alpco) and high molecular weight adiponectin (80-ADPHU-E01; ALPCO) were determined using ELISA, following the manufacturer’s instructions.

Plasma levels of total GIP, total GLP-1 and glucagon were measured as previously described using validated immunoassays^97^.

### Calculation of a PRS

Individuals from PLIS were genotyped onto two different versions of the Illumina Global Screening Array chip (280 on GSA-MD-24v1 and 549 on GSA-MD-24v2). A previously described set of 205 SNPs for predicted VAT, including only genome-wide substantial variants described in ref. ^24^ were selected and subsequently restricted to 186 SNPs. Quality control was performed on all participants using the PLINK 1.9 software. SNPs exclusion criteria included missingness threshold of >5%, minor allele frequency <1% and Hardy–Weinberg equilibrium with *P* < 0.05. The summary statistics were downloaded from the NHGRI–EBI GWAS on 8 October 2024 for study GCST008744 (refs. ^24,98^).

### Statistical analysis

**Extended Data Fig. 9:**
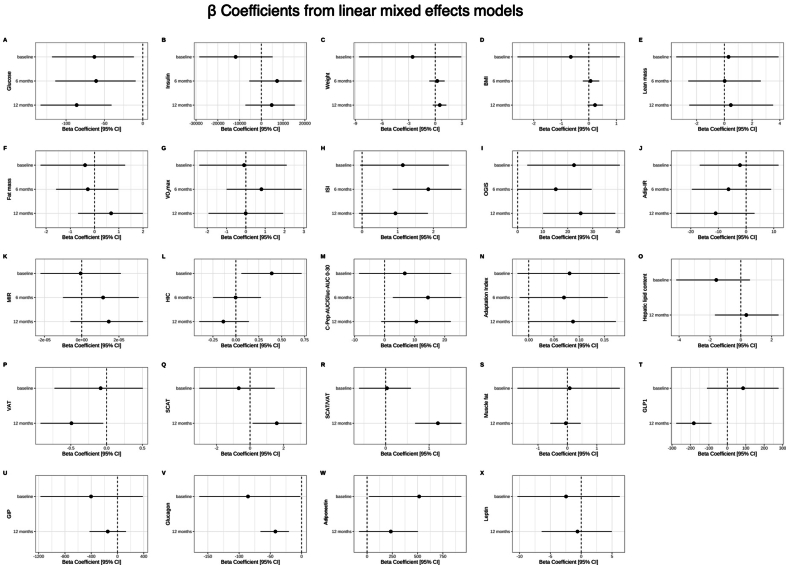
β Coefficients from linear mixed effects models. β coefficients for group (baseline) and group: time (6 months, 12 months) with 95% confidence intervals from linear mixed effects models reported above. Glucose (**a**, R: n = 51; NR: n = 183), insulin (**b**, R: n = 51; NR: n = 183), weight (**c**, R: n = 51; NR: n = 183), BMI (**d**, R: n = 51; NR: n = 183), lean mass (**e**, R: n = 49; NR: n = 170 NR), fat mass (**f**, n = 49; NR: n = 171), VO_2_ max (**g**, R: n = 17; NR: n = 47), insulin sensitivity index (**h**, R: n = 51; NR: n = 183), oral glucose insulin sensitivity index (**i**, R: n = 51; NR: n = 183), adipose tissue insulin resistance index (**j**, R: n = 21; NR: n = 109), muscle insulin resistance index (**k**, R: n = 51; NR: n = 183), hepatic insulin clearance (**l**, R: n = 51; NR: n = 183), C-peptide AUC 0-30 min/glucose AUC 0-30min (**m**, R: n = 51; NR: n = 183), adaptation index of β-cell function (**n**, R: n = 51; NR: n = 183), hepatic lipid content (**o**, R: n = 51; NR: n = 183), visceral adipose tissue (**p**, R: n = 51; NR: n = 183), subcutaneous adipose tissue (**q**, R: n = 51; NR: n = 183), ratio of subcutaneous to visceral adipose tissue (**r**, R: n = 51; NR: n = 183), muscle fat (**s**, R: n = 16; NR: n = 27), glucagon-like peptide 1 (**t**, R: n = 32; NR: n = 131), gastric inhibitory peptide (**u**, R: n = 16; NR: n = 27), glucagon (**v**, R: n = 16; NR: n = 27), adiponectin (**w**, R: n = 43; NR: n = 169), leptin (**x**, R: n = 43; NR: n = 149) in PLIS. β-coefficients are depicted as point estimates and 95% confidence intervals.

Linear mixed effects models with group (that is, R and NR), time point and the interaction group × time point as model terms adjusted for age, sex, BMI, risk stratification and intervention intensity as fixed effects and each individual as random effect were fitted to analyze longitudinal data. Additionally, the model evaluating insulin secretion included insulin sensitivity as fixed effect. All group × time *P* values were corrected for multiplicity using the Benjamini–Hochberg procedure. To avoid overreliance on significance testing and dichotomization, we report β coefficients for all models, including 95% confidence intervals in Extended Data Fig. 9. Diagnostic plots were plotted for each model to ensure normal distribution of residuals by visual inspection. For group-wise, cross-sectional comparisons, two-sided Wilcoxon rank-sum tests were performed. Resulting *P* values were also corrected for multiplicity of comparisons (Benjamini–Hochberg). T2D incidence up to almost 9 years after intervention end was compared through risk ratios and Fisher’s exact test using the epitools package, version 0.5-10.1. A Kaplan–Meier curve with log-rank test is also reported.

Statistical analyses were performed using RStudio (version 2024.12.0 + 467) with R (version 4.4.1). In the PLIS dataset, missing values for liver, subcutaneous, and visceral fat were imputed using the mouse package (version 3.16.0). In the DPP dataset, no imputation was performed.

### Reporting summary

Further information on research design is available in the Nature Portfolio Reporting Summary linked to this article.

## Online content

Any methods, additional references, Nature Portfolio reporting summaries, source data, extended data, supplementary information, acknowledgements, peer review information; details of author contributions and competing interests; and statements of data and code availability are available at 10.1038/s41591-025-03944-9.

## Supplementary information

Supplementary information Supplementary Tables 1–3.

Reporting Summary

## Figures and Tables

**Extended Data Fig. 8:**
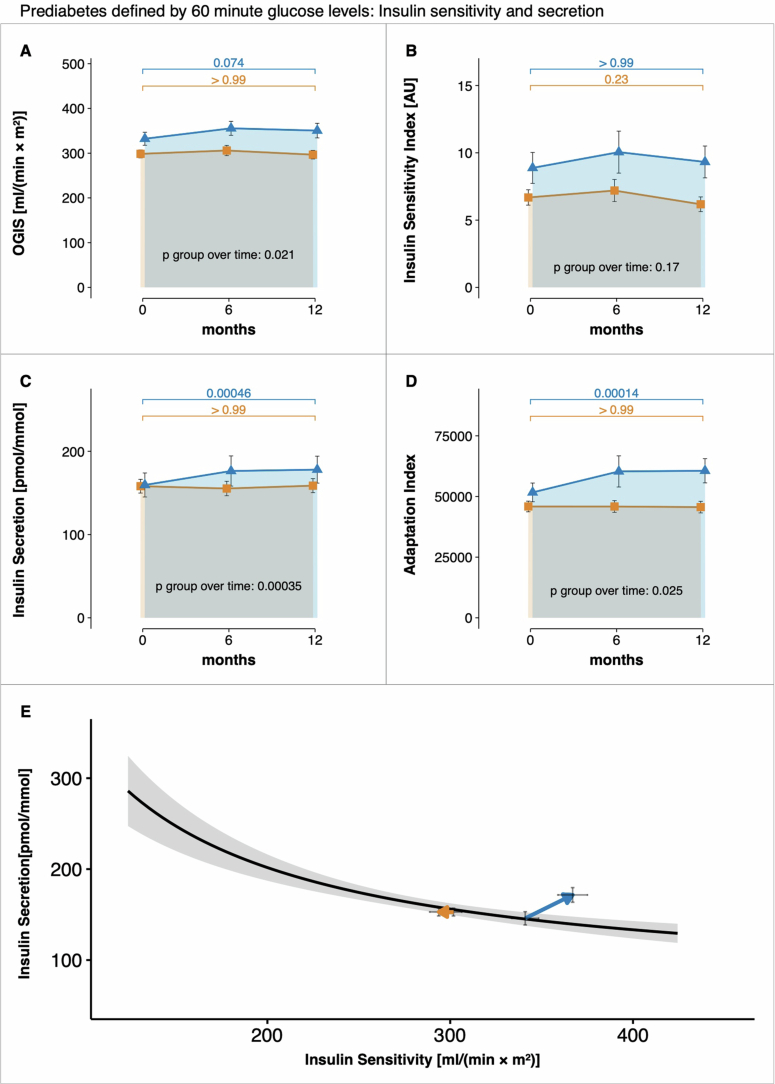
Prediabetes defined by 60-min glucose levels—insulin sensitivity and secretion. Indexes of insulin sensitivity (**a**, **b**), insulin secretion (**c**) and beta cell function (**d**) in PLIS with response defined as return of 60min glucose during the OGTT below 155 mg/dL. P values directly above the x axes indicate change in R versus NR over time (that is, interaction term of group and time) derived from mixed effects models. P values directly above the colored bars indicate the difference between baseline and 12 months for the responder group (blue) and the non-responder group (orange) as post-hoc corrected comparisons derived from the respective mixed effects models. **e**, The hyperbolic relationship between insulin sensitivity and secretion. Arrow base indicates coordinate at baseline, arrow tip indicates coordinate at 12 months for the R group and the NR group, respectively. Means of raw values are depicted with error bars indicating 95% confidence intervals for **a**-**d**. In **e**, the base of the arrows depict medians at baseline and the respective tip after 1 year of LI. Error bars depict s.e.m. R: n = 61; NR: n = 171 for each panel.

**Extended Data Table 1:**
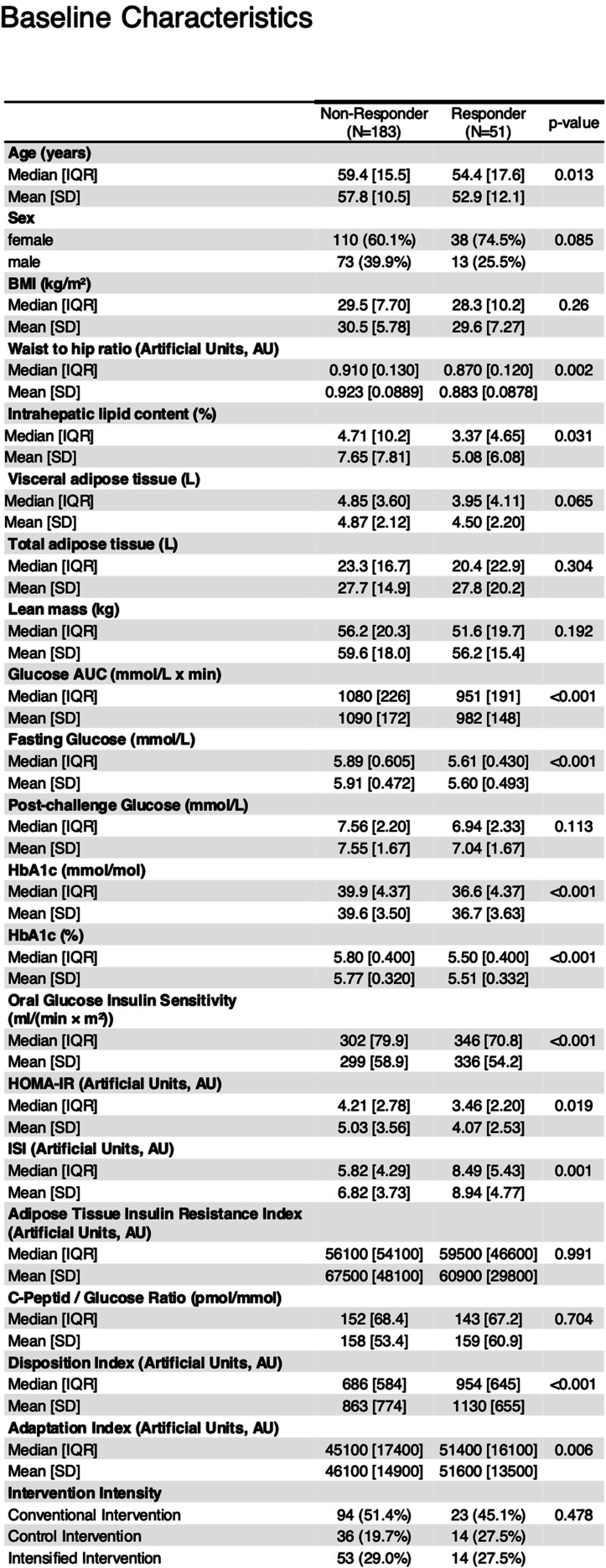
Baseline characteristics

Baseline characteristics of individuals participating in the Prediabetes Lifestyle Intervention Study (PLIS). PLIS is a multicenter, risk-stratified, randomized, controlled study examining the effectiveness of different lifestyle interventions in improving glycemic control. s.d. = standard deviation; IQR = inter-quartile range. P values are derived from two-sided Wilcoxon rank sum tests or chi-squared tests. Exact p-values: glucose AUC p = 6.37 × 10^−5^; fasting glucose p = 7.57 × 10^−5^; HbA1c both p = 1.58 × 10^−6^; oral glucose insulin sensitivity index p = 7.11 × 10^−5^; disposition index p = 0.00029.

## Data Availability

The datasets from this study are not publicly accessible due to national data protection regulations and ethical restrictions designed to protect participant privacy. They can be requested after publication by contacting the corresponding author. Each request will be reviewed by the PLIS data steering committee, and approved access will require a formal data use agreement. The PLIS data steering committee will respond to requests within 3 months. Data from the DPP referenced in this study can be obtained through the U.S. NIDDK Central Repository (https://repository.niddk.nih.gov/)^99^. These datasets include demographic details, anthropometric and laboratory assessments, imaging data and time-to-diabetes diagnosis.

## Code availability

No custom code was generated for this study. Previously published packages and functions have been referenced.
