# Supplementary material for: Prevention of type 2 diabetes through prediabetes remission without weight loss
Source: Nat Med. 2025 Sep 29;31(10):3330–40. doi: 10.1038/s41591-025-03944-9 (PMC12532634; doi:10.1038/s41591-025-03944-9)
Supplement: Supplementary file 1 — Supplementary Tables 1–3. [file 41591_2025_3944_MOESM1_ESM.pdf]

nature medicine

Supplementary information https://doi.org/10.1038/s41591-025-03944-9

Prevention of type 2 diabetes through prediabetes remission without weight loss

In the format provided by the authors and unedited

Supplementary Table 1

Biomarkers of Inflammation - Absolute Values

Biomarker	Unit	Baseline					12 months					
		**Responder**	SD	**Non-Responder**	SD	p	**Responder**	SD	**Non-Responder**	SD	p	p group over time
CX3CL1	[pg/mL]	32.75	12.15	46.18	22.83	0.0065	34.94	10.64	40.19	12.84	0.23	0.44
ICAM	[pg/mL]	28440.03	11679	33537.33	12620.96	0.21	34334.09	6583.7	38309.69	9711.68	0.19	0.78
IL17	[pg/mL]	6.14	4.48	6.67	4.89	0.78	6.41	4.93	6.15	4.67	0.87	0.78
IL18	[pg/mL]	40.57	22.26	54.16	30.94	0.10	45.81	17.93	55.2	29.76	0.23	0.66
IL8	[pg/mL]	6.29	4.03	9.44	7.07	0.049	7.09	3.19	8.85	4.59	0.22	0.66
IP10	[pg/mL]	296.8	186.16	557.77	432.42	0.0030	339.43	187.45	476.86	261.17	0.12	0.44
MCP1	[pg/mL]	17.02	6.41	20.16	10.3	0.21	15.22	6.2	17.68	8.79	0.33	0.69
MIG	[pg/mL]	110.66	49.87	214.24	159.87	0.00056	117.63	49.41	198.05	117.39	0.00068	0.66
MIP1a	[pg/mL]	0.98	0.57	1.6	0.93	0.0056	1.33	0.58	1.69	0.83	0.19	0.52
MIP1b	[pg/mL]	341.31	154.22	337.95	143.31	0.99	397.19	169.88	354.9	150	0.52	0.44
Osteopontin	[pg/mL]	7294.7	5133.4	11866.64	9199.56	0.027	9901.36	5875.1	9537.21	6661.09	0.87	0.32
RANTES	[pg/mL]	6025.52	4919.5	6719.7	5688.62	0.76	6848.47	5216.5	6616.02	5548.11	0.87	0.66
sTNFR1	[pg/mL]	1176.88	654.95	1967.5	1252.24	0.0044	1295.14	670.53	1351.14	812.12	0.87	0.32
sTNFR2	[pg/mL]	180.25	135.52	370.32	250.97	0.0014	214.48	134.26	277.25	209.05	0.27	0.32
TNFa	[pg/mL]	218.2	139.54	217.76	127.27	0.99	254.02	146.45	231.93	138.37	0.74	0.69
TNFb	[pg/mL]	6.15	4.62	7.46	10.15	0.62	5.9	3.66	6.7	5.91	0.68	0.99
VCAM	[pg/mL]	37953.81	15960	41193.06	16267.7	0.62	44308.71	7540.6	47237.8	11969.2	0.35	0.97

Supplementary Table 2

Biomarkers of Inflammation - Relative Values

Biomarker	Unit	Change (per person)				
		**Responder**	SD	**Non-Responder**	SD	p
CX3CL1	%	15.29	45.63	-2.32	35.85	0.48
ICAM	%	35.5	52.05	22.03	29.09	0.76
IL17	%	-5.62	67.88	27.97	126.35	0.64
IL18	%	15.09	31.04	7.09	30.45	0.64
IL8	%	53.31	112.97	18.58	71.37	0.76
IP10	%	20.52	38.52	8.55	76.93	0.27
MCP1	%	-2.73	44.15	-9.81	34.31	0.76
MIG	%	10.36	37.72	13.15	58.18	0.76
MIP1a	%	49.76	65.26	21.54	47.97	0.27
MIP1b	%	9.5	9	4.55	15.23	0.27
Osteopontin	%	87.95	106.31	55.37	172.44	0.27
RANTES	%	3.57	37.05	0.01	33.81	0.64
sTNFR1	%	27.72	66.66	-5.64	63.04	0.27
sTNFR2	%	61.48	104.31	10.21	86.48	0.27
TNFa	%	7.42	12.19	5.09	16.35	0.77
TNFb	%	30.44	165.25	337.16	2137.96	0.36
VCAM	%	28.57	40.82	23.36	30.51	0.85

Supplementary Table 3:
Baseline Characteristics in DPP

	Non-Responder (N=469)	Responder (N=25)	p-value
**Treatment**			
Lifestyle	85 (18.1%)	4 (16.0%)	0.998
Placebo	384 (81.9%)	21 (84.0%)	
**Age [years]**			
Mean [SD]	50.0 [10.6]	45.6 [9.68]	0.0335
Median [Q1, Q3]	49.0 [42.8, 57.0]	44.2 [40.3, 51.4]	
**Sex**			0.256
Female	325 (69.3%)	20 (80.0%)	
Male	144 (30.7%)	5 (20.0%)	
**BMI [kg/m^2]**			
Mean [SD]	34.0 [6.97]	31.5 [3.79]	0.169
Median [Q1, Q3]	32.4 [28.9, 37.4]	31.1 [28.8, 33.6]	
**Waist to hip ratio**			
Mean [SD]	0.923 [0.0831]	0.901 [0.0745]	0.189
Median [Q1, Q3]	0.921 [0.861, 0.985]	0.893 [0.859, 0.944]	
**Visceral fat region L2/L3 [cm^2]**			
Mean [SD]	201 [90.7]	155 [62.2]	0.184
Median [Q1, Q3]	181 [138, 251]	139 [127, 177]	
**Visceral fat region L4/L5 [cm^2]**			
Mean [SD]	159 [67.2]	122 [28.0]	0.117
Median [Q1, Q3]	143 [108, 194]	128 [96.6, 143]	
**Glucose AUC**			
Mean [SD]	1060 [98.8]	1010 [118]	0.0329
Median [Q1, Q3]	1050 [989, 1120]	993 [938, 1100]	
**Fasting glucose [mmol/L]**			
Mean [SD]	5.90 [0.370]	5.64 [0.186]	<0.001
Median [Q1, Q3]	5.83 [5.56, 6.11]	5.56 [5.50, 5.67]	
**30-minute glucose [mmol/L]**			
Mean [SD]	9.36 [1.32]	8.90 [1.52]	0.144
Median [Q1, Q3]	9.28 [8.44, 10.1]	9.00 [7.61, 9.89]	
**120-minute glucose [mmol/L]**			
Mean [SD]	9.07 [0.941]	8.80 [0.961]	0.119
Median [Q1, Q3]	8.89 [8.28, 9.78]	8.61 [7.94, 9.28]	
**HbA1c [%]**			
Mean [SD]	5.79 [0.411]	5.33 [0.535]	<0.001
Median [Q1, Q3]	5.90 [5.50, 6.10]	5.30 [5.20, 5.60]	
**Triglycerides [mmol/L]**			
Mean [SD]	1.82 [1.00]	1.47 [0.645]	0.111
Median [Q1, Q3]	1.61 [1.15, 2.24]	1.50 [1.04, 1.86]	
**ISI_0**			
Mean [SD]	0.201 [0.138]	0.236 [0.144]	0.176
Median [Q1, Q3]	0.164 [0.119, 0.244]	0.221 [0.132, 0.273]	
**CIR_{30}**			
Mean [SD]	0.613 [0.363]	0.708 [0.561]	0.684
Median [Q1, Q3]	0.550 [0.348, 0.765]	0.577 [0.376, 1.06]	

Supplementary Legends

Supplemetary Table 1:

Absolute values of biomarkers of inflammation at baseline and5.91 after intervention in PLIS. Chemokine (C-X3-C motif) ligand 1 (CX3CL1), intercellular adhesion molecule 1 (ICAM), interleukin 17 (IL17), interleukin 18 (IL18), interleukin 8 (IL8), interferon gamma-induced protein 10 (IP10), monocyte chemoattractant protein 1 (MCP1), monokine induced by gamma interferon (MIG), macrophage inflammatory protein 1-alpha (MIP1a), macrophage inflammatory protein 1beta (MIP1b), regulated on activation, normal T-cell expressed and secreted (RANTES), soluble tumor necrosis factor receptor 1 (sTNFR1), soluble tumor necrosis factor receptor 2 (sTNFR2), tumor necrosis factor alpha (TNFa), tumor necrosis factor beta (TNFb), vascular cell adhesion protein 1 (VCAM). P values are obtained from two-sided Wilcoxon rank sum tests with adjustment for multiple comparisons via the Benjamini-Hochberg procedure. P group over time indicates the interaction term of R and NR and time from mixed effects models. n = 36 R vs 132 NR.

Supplementary Table 2:

Percent change from baseline of biomarkers of inflammation in PLIS. P values are obtained from linear mixed effects models adjusting for age, sex, BMI, risk group assignment and intervention intensity. Adjustment for multiple comparisons was done via the Benjamini-Hochberg procedure. n = 36 R vs 132 NR.

Supplementary Table 3:

DPP baseline characteristics. Values in parentheses represent standard deviation or interquartile range. P values are derived from two-sided Wilcoxon rank sum tests or chi-squared tests. For visceral adipose tissue in region L2/L3 and region L4/L5 N=8 R vs 160 NR.

1. EXECUTIVE SUMMARY 1-1

1. EXECUTIVE SUMMARY

Background

Non-insulin-dependent diabetes mellitus (NIDDM) is rapidly becoming the most common chronic disease in the United States, with more than 7% of the adult population affected and 600,000 new cases per year. NIDDM is even more common in the elderly and in minority populations including African Americans, Hispanic Americans, Asian and Pacific Island Americans, and Native Americans. In these populations, NIDDM may be present in 10% to as much as 50% of the adult population. Diabetes is accompanied by a multitude of severe long-term complications that ultimately cause more adult cases of blindness, renal failure, and amputations than any other disease in the United States (U.S.). In addition, persons with NIDDM have a 2 to 4 fold increased risk for cardiovascular disease and stroke. Owing largely to the high costs of caring for NIDDM and its attendant long-term complications, total health care expenditure for diabetes has been estimated at approximately 100 billion dollars per year, or 12% of total U.S. health care expenditures.

The enormous human and financial costs that accompany NIDDM, and the difficulty in treating it effectively once it has developed, make it an appropriate target for prevention. In 1993, the National Institute of Diabetes and Digestive and Kidney Diseases, issued a request for proposals for a study with the objective of preventing the development of NIDDM in adults, with a major emphasis on the minority communities in which NIDDM is so prevalent. Clinical centers were selected to participate in recruiting a target cohort comprising approximately 50% minorities from the populations mentioned above.

The investigators of the Diabetes Prevention Program (DPP) designed the following consensus protocol between August, 1994 and November, 1995.

Objective

The principal objective of the DPP is to prevent or delay the development of NIDDM in those persons who are at high risk for its development by virtue of having impaired glucose tolerance. Impaired glucose tolerance represents a less severe stage of blood glucose abnormalities that often precedes NIDDM.

Study Populations

Volunteers will be recruited from populations known to be at particularly high risk for impaired glucose tolerance and NIDDM including the following: persons with a family history of NIDDM, the elderly, overweight individuals, women with a history of diabetes during pregnancy ("gestational diabetes"), and minority populations including African Americans, Hispanic Americans, Asian and Pacific Island Americans, and Native Americans. In order to be eligible, persons who are older than 25 years will have to demonstrate impaired glucose tolerance with plasma glucose levels 95-125 mg/dL (5.3-6.9 mmol/L) fasting and 140- 199 mg/dL (7.8 - 11.0 mmol/L) two hours after a 75 gram oral glucose tolerance test. The study-wide goal is that approximately 50% of the study population be composed of minorities and approximately 20% be 65 years of age or older.

Study Interventions

Three interventions were selected based on their potential efficacy in ameliorating abnormal glucose metabolism in IGT and on their safety and tolerable profile of side-effects. The interventions include an intensive lifestyle intervention and two pharmacological intervention. The intensive lifestyle intervention focuses on a healthy diet to achieve and maintain at least a 7% loss of body weight and to achieve at least 150 min/week of moderate intensity exercise, and maintain this level of physical activity throughout the DPP. Attempts to modify diet and exercise are flexible and sensitive to cultural differences and acceptable to the specific communities in which they are implemented. Standard lifestyle recommendations, which include conventional instructions regarding diet and exercise, will be provided to all participants, including a placebo treated group which will serve as the control group for the study. The pharmacological interventions include the biguanide metformin and the thiazolidinedione troglitazone.

Randomization to the troglitazone pharmacological intervention was suspended on May 27, 1998, and discontinued by the NIDDK on June 3, 1998. The NIDDK, with input from the DPP Data Monitoring Board, decided to discontinue use of troglitazone in the DPP based on liver toxicity with hepatic failure (see section 5.3.1).

Outcomes

The primary outcome is the development of diabetes according to American Diabetes Association criteria (fasting plasma glucose level ≥ 126 mg/dL [7.0 mmol/L] or 2-hour plasma glucose ≥ 200 mg/dL [11.1 mmol/L], after a 75 gram OGTT, and confirmed with a repeat test). Secondary outcomes will focus on cardiovascular disease and its risk factors and changes in glycemia, insulin secretion and sensitivity, obesity, physical activity and nutrient intake, quality of life, and the occurrence of adverse events.

Design and Power

The study is a randomized clinical trial. Eligible volunteers will be stratified according to center and assigned to one of three treatment groups during a two and two-thirds year recruitment period. The pharmacological interventions are double blinded and placebo controlled. After randomization, participants have quarterly clinical evaluations and have, in addition, a fasting plasma glucose at semi-annual visits and a 75 gram oral glucose tolerance test at annual visits. All participants will be followed for three and one-third years after the study-wide closing date for recruitment, resulting in 3 1/3 to 6 years of participant follow-up.

Three thousand participants (one thousand per group) will provide 90% power to detect a 33% reduction in the progression to diabetes, assuming an annual rate of progression to diabetes in the control group of at least 6.5% and a level of significance of 5% (two-sided) with adjustment for pairwise comparisons of the three treatment groups.

Analyses

For the primary outcome, time to development of diabetes, product-limit life-table distributions of each intervention group and the control group will be compared using the log-rank test statistic. The primary analyses will include all participants in their randomly assigned treatment group, regardless of adherence to the assigned treatment (intention-to-treat principle).

2. OBJECTIVES *2-1*
2.1 Primary Research Question 2-1
2.2 Secondary Research Questions 2-1
2.3 Subgroup Research Questions 2-1
2.3.1 Mechanism of Metformin 2-1

2. OBJECTIVES

2.1 Primary Research Question

The objective of the Diabetes Prevention Program (DPP) is to prevent or delay the onset of diabetes among persons at high risk using interventions designed to improve abnormal glucose metabolism. The primary research question of the DPP is to determine the safety and efficacy of two interventions (intensive lifestyle and metformin) relative to a control group (standard lifestyle recommendations) in preventing or delaying the confirmed development of diabetes.

2.2 Secondary Research Questions

Secondary research questions of the DPP include assessing differences over time between the three treatment groups with regard to the following:

- Development of diagnostic levels of fasting hyperglycemia ($\geq$ 140 mg/dL [7.8 mmol/L]).
- Reversal of IGT to a state of normal glucose tolerance.
- Change in hyperglycemia as measured by glycohemoglobin and fasting and 2 hr. plasma glucose.
- Change in insulin secretion and sensitivity.
- Change in body composition.
- Change in health related quality of life.
- Change in renal function.
- Occurrence and magnitude of risk factors for cardiovascular disease.
- Change in atherosclerosis measured by carotid ultrasound.
- Occurrence of cardiovascular morbidity and mortality.
- Occurrence and magnitude of major adverse events.
- Costs and cost-effectiveness of the interventions.

2.3 Subgroup Research Questions

Other research questions of the DPP include assessing subgroups of participants with regard to the following:

- Consistency of the effects of the interventions across the ethnic, age, and other selected subgroups.

Demographic, clinical, biochemical, and psychosocial parameters that predict response to the interventions.

2.3.1 Mechanism of Metformin

To clarify the mechanism of metformin's salutary effect on diabetes development during the DPP (acute pharmacological vs. long-term effect on metabolism) by performing a short-term washout study during which medications will be held and subsequent development of diabetes assessed.

3. BACKGROUND OF THE DPP *3-1*
3.1 Prevalence of NIDDM and IGT 3-1
3.2 Morbidity and Mortality 3-1
3.3 Risk Factors For NIDDM and IGT 3-2
3.4 Progression of IGT to NIDDM 3-3
3.5 Etiology 3-3
3.6 IGT and Macrovascular Disease 3-4
3.7 Interventions That May Decrease Progression from IGT to NIDDM 3-4
3.7.1 Pharmacological Intervention Studies 3-4
3.7.2 Behavioral Intervention Studies-Diet and Exercise 3-5
3.8 Rationale for the Study 3-5
3.9 Rationale for Medication Washout 3-6

3. BACKGROUND OF THE DPP

3.1 Prevalence of NIDDM and IGT

An estimated 12-15 million people in the United States have diabetes mellitus, greater than 90% of whom have non-insulin-dependent diabetes mellitus (NIDDM) (Harris, et al., 1987). One-half of the NIDDM cases are undiagnosed. Diabetes prevalence increased dramatically in the United States during the 1960s and 1970s by approximately 50% every 7 years. This large increase was probably due to a shift in the age of the population and more accurate ascertainment. In the past decade, incidence in the U.S. has stabilized (4% increase since 1980), and is presently 600,000 new cases per year. However, the incidence worldwide, and especially in developing countries, is steadily rising. The World Health Organization has concluded that "an apparent epidemic of diabetes has occurred . . . which is strongly related to lifestyle and economic change" (King, et al., 1991).

Diagnosed NIDDM is only the tip of the iceberg of an epidemic of glucose intolerance. Impaired glucose tolerance (IGT) is even more prevalent than NIDDM. IGT is characterized by glycemia between normal and overtly diabetic levels (NDDG and WHO criteria). In addition to being a major risk factor for the development of NIDDM, IGT is associated with an increased risk for macrovascular disease. IGT is not, however, associated with the diabetes-specific complications of retinopathy, nephropathy or neuropathy (Eriksson, et al., 1994). Among American adults 20-74 years of age, approximately 18% have some form of glucose intolerance (7% with NIDDM and 11% with IGT, Harris, et al., 1987, from NHANES) according to WHO criteria (WHO, 1980). Other population-based studies in the United States (Meigs, et al., 1995) and Europe (Garancini, et al., 1995) have revealed almost identical prevalence of IGT and NIDDM as shown in the National Health and Nutrition Examination Survey (NHANES). The major impact of aging is evidenced by the 42% prevalence of IGT or NIDDM in the population between 65 and 74 (Harris, et al., 1987; Harris, 1989). The risk of abnormal glucose tolerance is greatly increased in minority populations such as African Americans, Hispanic Americans, Native Americans, and Asian and Pacific Islander Americans (Harris, et al., 1987; Lee, et al., 1995; Haffner et al., 1991; Zimmet, et al., 1982; Curb, et al., 1991; Mau, et al., 1995; Fujimoto, 1995). On the basis of the continued growth of the fraction of the U.S. population older than age 65, and of ethnic and racial groups at particularly high risk to develop NIDDM and IGT, the overall prevalence of NIDDM and IGT will continue to increase in the next decade.

3.2 Morbidity and Mortality

Diabetes mellitus causes long-term, diabetes-specific complications, including retinopathy, nephropathy, and neuropathy, and is associated with a 2-7 fold increase in risk for cardiac, peripheral, and cerebral vascular disease (Nathan, 1993). The clinical characteristics of the diabetes-specific complications are similar among all clinical forms of diabetes. The prevalence and incidence of these complications are somewhat less in NIDDM than IDDM; however, because of the much larger size of the NIDDM population, it contributes the majority of cases of diabetic retinopathy, nephropathy, and neuropathy. Diabetes is the major cause of new onset blindness in adults (12,000 to 24,000 cases per year), and the most common cause of end-stage renal disease (19,800 per year) and lower extremity amputation (54,000 per year) (NIDDK, 1995). With regard to the nonspecific macrovascular complications, women are disproportionately affected. Whereas men with NIDDM have a 2-3 fold increased risk for cardiovascular disease compared with nondiabetic men, women with NIDDM have a 4-5 fold increased risk for cardiovascular disease compared with nondiabetic women (Kannel, et al., 1979; Barrett-Connor, et al., 1991).

Current data from the Centers for Disease Control and Prevention (Geiss, et al., 1993) and the National Center for Health Statistics (Kovar, et al., 1987) reveal the staggering impact of diabetes and its complications in the U.S. Diabetes is among the 10 leading causes of death, the underlying cause in over 40,000 deaths per year, a contributing cause in perhaps another 300,000 deaths, and a named cause in over 150,000. The majority of adults with NIDDM die from cardiovascular, cerebrovascular or peripheral vascular disease. Among deaths related to diabetes, cardiovascular disease is the cause in more than 80,000 cases per year. Diabetes is the primary diagnosis in almost 500,000 hospital discharges per year and a secondary diagnosis in almost 3 million.

The annual direct cost of medical care for diabetes mellitus, much of which goes to treating the long-term complications and their sequelae, was estimated at $20 billion in 1987. The total cost, including lost productivity, exceeded $40 billion per year. More recent estimates, based on the total health care expenditures for diabetic

persons, have more than doubled the cost of diabetes to approximately $100 billion per year, or 12% of total U.S. health care expenditure (Rubin, et al., 1994).

In the United States, a disproportionate fraction of morbidity and mortality associated with glucose intolerance, especially with regard to nephropathy, is borne by African Americans and other racial minorities, especially Native Americans. The estimated prevalence of end-stage renal disease in persons with diabetes is 160% greater in African Americans than in Caucasians (Cowie, et al., 1989).

Among diabetic African Americans with end-stage renal disease, 77% have NIDDM. Among Caucasians, in whom insulin-dependent diabetes mellitus is relatively more prevalent than in African Americans, 42% of the diabetics with end-stage renal disease have NIDDM (Cowie, et al., 1989). End-stage renal disease is especially common among Zuni (Hoy, et al., 1987), Navajo (Megill, et al., 1988), and Pima (Nelson, et al., 1988) Indians. Among diabetic Pima Indians, the incidence rates of end-stage renal disease are at least as high as in Caucasians with IDDM of the same duration of diabetes (Nelson, et al., 1988).

3.3 Risk Factors For NIDDM and IGT

Among the most important factors associated with increased risk of developing NIDDM are obesity, advanced age, a history of IGT or of gestational diabetes mellitus (GDM), a positive family history of NIDDM, minority race, and lipid abnormalities (American Diabetes Association, 1990; Barrett-Connor, 1989; Jarrett, 1989; Everhart, et al., 1985; West, 1978; O'Sullivan and Mahan, 1965; Burchfiel, 1995; among many). Although many of these were originally shown to be associated with a risk of NIDDM, it is now generally accepted that these factors are associated with lower degrees of glucose intolerance, such as IGT, and the risk of progression along the continuum culminating in NIDDM.

West (1978) stressed the importance of obesity as the common, dominant characteristic of NIDDM. In virtually all populations, obesity is associated with increased risk of NIDDM, of IGT, and of progression from IGT to NIDDM. Earlier investigations assessed obesity as percent of ideal body weight (e.g. IBW>120%), or as body mass index (BMI). However, increased centrally deposited fat, as conveniently measured by an increased waist-to-hip ratio (WHR), is in some studies a stronger risk factor than obesity (McKeigue, et al., 1992; Haffner, et al., 1987; Ohlson, et al., 1985; among many).

Race is also strongly associated with increased risk of glucose intolerance. Based on the National Health and Examination Survey II (NHANES II, 1976-80), the prevalence of NIDDM in all age groups is 50 to 70% higher among African Americans than among Caucasians (Harris, 1990; Cowie, et al., 1993), and is even higher for several other racial minorities (Flegal, et al., 1991; Harris, 1991). Cuban Americans have approximately 60% greater prevalence, and Mexican Americans and Puerto Rican Americans approximately 120% greater prevalence of NIDDM, compared to the Caucasian population (Harris, 1991). The prevalence among Native Americans, especially the Pima Indians of the southwest, is approximately 8 times greater (800%) than in Caucasians (King, et al., 1993; Knowler, et al., 1978).

The relationships between risk factors for NIDDM are complex. For example, among African Americans there is a 50% excess risk at 125% of ideal body weight. Although the prevalence of obesity and associated risk factors for NIDDM is higher among African Americans than Caucasians, African Americans still have an excess risk of glucose intolerance after adjustment for these factors (Cowie, et al., 1993; Harris, 1990). Overall, the age and sex-adjusted prevalence of NIDDM is estimated to be 60% higher among African Americans than Caucasians and approximately 50% higher after adjustment for other factors including obesity (Cowie, et al., 1993).

A history of NIDDM in first degree relatives is also strongly predictive of glucose intolerance. In the adult population of the United States, a positive family history imparts a 2.7 fold risk of NIDDM after adjustment for other risk factors (Cowie, et al., 1993). Based on NHANES II, the risk of NIDDM among Caucasians was 1.8 fold higher in those with one family member with history of NIDDM, and 3.6 fold higher for those with two or more family members compared to persons with no known family history of NIDDM. Similar relative risks were found for African Americans (Harris, 1990) and Pima Indians (Knowler, et al., 1981). When the offspring of lower weight persons with NIDDM are compared with offspring of more obese persons with NIDDM, family history of NIDDM plays a relatively greater role as a risk factor for NIDDM, at least in the Pimas (Hanson, et al., 1995). This suggests a relatively stronger familial, presumably genetic, influence on the development of NIDDM when obesity is not present.

Some (West, 1978), but not all (King, et al., 1993) studies have suggested that female gender is a risk factor for NIDDM. Based on the NHANES II data, females have approximately 50% greater risk of NIDDM after adjustment for race and body mass (Cowie, et al., 1993). The effect of race and obesity interact with gender and

age. Among African Americans, males have the highest risk up to age 64, females thereafter (Harris, 1989). Also, the risk gradient for obesity is greatest among African American females (Cowie, et al., 1993). Other associated risk factors for NIDDM and IGT include a sedentary lifestyle, low levels of physical activity, and low education and socioeconomic status (Haffner, et al., 1991; Mitchell, et al., 1993; Manson, et al., 1992; Perry, et al., 1995; Monterrosa, et al., 1995; Helmrich, et al., 1991; Dowse, et al., 1991; Annuzzi, et al., 1985). Hypertension (e.g. Salomaa, 1991; Burchfiel, 1995; McPhillips, et al., 1990) and dyslipidemia (Perry, et al., 1995; Burchfiel, et al., 1995) are also associated with the development of NIDDM.

Gestational diabetes mellitus (GDM) represents a state of glucose intolerance that occurs during pregnancy. The mild nature and transience of the glucose intolerance, which almost always improves shortly after delivery, does not generally result in long-term complications for the mother. However, untreated GDM may result in adverse events for the fetus, including macrosomia and perinatal death. In addition, approximately 30-50% of women with a history of GDM progress to NIDDM within 5 to 10 years of their affected pregnancy (O'Sullivan and Mahan, 1965, O'Sullivan, 1991), making GDM a major risk factor for NIDDM. As with persons with IGT, specific factors have been identified in women with GDM that increase the risk for development of post-partum NIDDM. These include higher pre-pregnancy BMI and antepartum fasting glucose level, requirement for insulin during pregnancy, low insulin secretion during pregnancy, hyperglycemic post-partum fasting and OGTT glucose levels, and recurrence of GDM with subsequent pregnancies (Metzger, et al., 1985; Grant, et al., 1986; Lam, et al., 1991; Coustan, et al., 1993; Damm, et al. 1995; Kjos, et al., 1995).

3.4 Progression of IGT to NIDDM

Approximately 30 to 40% of persons identified as having IGT eventually develop NIDDM. The rate of progression from IGT to NIDDM is 1-5% per year, depending on the specific population. (NDDG, 1979; O'Sullivan, et al., 1968; Birmingham Diabetes Survey, 1976; Jarrett, et al., 1979; Beaty, et al., 1982; Saad, et al., 1988). Risk factors that increase the frequency and perhaps rate of progression from IGT to NIDDM include: higher levels of fasting or post-challenge hyperglycemia (O'Sullivan, 1968; Birmingham, 1976; Jarrett, 1979; Sartor, 1980; Kadowaki, 1984); family history of NIDDM in some (Beaty, 1982; Savage, 1979), but not all (O'Sullivan, 1968) studies; greater levels of obesity (O'Sullivan, 1968; Colditz, 1990; Kadowaki, 1984); lower stimulated insulin levels in some studies (Kadowaki, 1984); and ethnicity (Zimmet, 1982).

3.5 Etiology

Clearly, many of the risk factors are interrelated through the pathophysiologic mechanisms that result in glucose intolerance and NIDDM. In the nondiabetic individual, glucose homeostasis is maintained by a balance of glucose uptake, predominately by muscle, and glucose influx from the gastrointestinal tract and the liver (DeFronzo, 1988). The balance is mediated by insulin, which suppresses hepatic glucose output and increases peripheral uptake and glucagon, which opposes insulin action on the liver. In IGT, which generally precedes NIDDM, peripheral glucose uptake is decreased because of decreased sensitivity to insulin action, so called insulin resistance. Insulin resistance is associated in part with obesity, but other genetic factors which have not been identified may predominate as the cause of insulin resistance in NIDDM. In any case, although increased insulin secretion, resulting in hyperinsulinemia, develops to compensate for the insulin resistance this is not completely sufficient, resulting in post-challenge glycemia. IGT, manifested by increased post-challenge glucose levels, but relatively normal fasting glycemia, occurs months to years prior to the development of NIDDM. Further decompensation, with increasing levels of fasting glycemia and development of NIDDM, results as insulin secretion deteriorates even further (Brunzell, et al., 1976; Saad, et al., 1988 and 1991).

The genetic basis of insulin resistance has been demonstrated in several populations (Warram, et al., 1990; Saad, 1991; Sicree, et al., 1987; Haffner, et al., 1990; Bergstrom, et al., 1990). Beta cell decompensation may also be inherited (O'Rahilly, et al., 1988; Eriksson, et al., 1989) or may be acquired, presumably through a glucotoxic effect on the beta cell (Leahy, 1990). Glucose intolerance progresses through stages of increasingly higher levels of post-challenge glycemia, mediated through decreased peripheral uptake, to frank diabetes with fasting hyperglycemia. This final stage is mediated by increased hepatic glucose output secondary to decreased insulin secretion and/or increased substrate delivery from the peripheral tissues.

As with many biological processes, however, there is substantial heterogeneity among populations (Chaiken et al., 1993; Banerji, et al., 1993; Banerji and Lebovitz, 1991). In lean patients with NIDDM, a beta-cell secretory defect may be the principal cause of their glucose intolerance. Recently described mutations in the glucokinase gene underlie some cases of an autosomally inherited form of NIDDM, called maturity onset diabetes

of the young (MODY). Glucokinase is the putative glucose sensor in the beta-cell; the mutations result in decreased insulin secretion.

Regardless of the pathogenic mechanisms that underlie the development of NIDDM, it is clear that obesity and inactivity, in addition to other factors, are *associated* with the risk of IGT and NIDDM in various populations. Therefore, the question addressed by the DPP is whether the risk associated with these factors is *reversible* through intervention. To the extent that pharmacological agents can ameliorate specific pathophysiologic mechanisms of NIDDM such as insulin resistance or decreased insulin secretion, they too may prevent progression from IGT to NIDDM.

3.6 IGT and Macrovascular Disease

Glucose intolerance is also associated with risk factors for macrovascular disease including hypertension, reduced high density lipoproteins (HDL), elevated triglycerides and, less consistently, with elevated low-density lipoproteins (LDL). The presence of many of these risk factors has been linked to the hyperinsulinemia that is associated with the prolonged period of insulin resistance preceding the development of NIDDM. Haffner et al. (1992) have shown that increasing fasting insulin levels at baseline and at follow-up are associated with higher risk of decompensation to NIDDM and hypertension with increased triglycerides and decreased HDL. In a retrospective analysis, Haffner et al. (1990) conclude that pre-diabetics have an atherogenic risk profile, possibly present for years, prior to the onset of glucose intolerance. Harris (1989), based on the NHANES II, McPhillips et al. (1990) based on the prospective Rancho Bernardo follow-up in the Lipid Research Clinic program, and Haffner et al. (1992), based on the San Antonio Heart Study, have all noted that individuals with IGT have a cardiovascular risk profile that is between that of individuals with NIDDM and those with normal glucose tolerance. In the Rancho Bernardo study, 23% of newly diagnosed IGT and 41% of newly diagnosed NIDDM patients had cardiovascular disease (Wingard, et al., 1993).

3.7 Interventions That May Decrease Progression from IGT to NIDDM

Despite the putative role for genetic factors in its development, onset of NIDDM may be prevented or delayed. Changes in diet and an increasingly sedentary lifestyle, with consequent increased body mass, have been associated with the development of NIDDM in recently industrialized populations (King, et al., 1984) and in migrating populations (Kawate, et al., 1979; Fujimoto, et al., 1987) that previously had a low prevalence of NIDDM. Moreover, studies in the U.S. population demonstrate the adverse consequences of decreased physical activity (Helmrich, et al., 1991; Manson, et al., 1991; Manson et al., 1992) and obesity (West, et al., 1971; Knowler, et al., 1981; Colditz et al., 1990) on the risk for developing NIDDM. These observations support the role of potentially reversible factors in the development of diabetes. Several risk factors for the progression from IGT to NIDDM have also been identified, including obesity (O'Sullivan and Mahan, 1968; Modan, et al., 1986; Kadowaki, et al., 1984), higher glucose levels within the IGT range (O'Sullivan and Mahan, 1968; Birmingham Diabetes Study, 1976; Sharp, et al., 1964; Jarrett, et al., 1979), increased fasting insulin concentrations (Saad, et al., 1991; Sicree, et al., 1987), and decreased insulin secretion (Brunzell, et al., 1976; Saad, et al., 1988).

Unfortunately, relatively few controlled clinical trials provide any direct information on the effect of manipulating these risk factors on the development of NIDDM. Moreover, the definitions of IGT and NIDDM have varied between trials, making projections of the results to current day definitions problematic. Several programs directed at achieving weight loss and/or improving cardiovascular conditioning have been examined to determine their effects on glucose metabolism and the development of NIDDM. In addition, a small number of prospective trials have studied drugs generally used to treat NIDDM to prevent its development from IGT. The summary below reviews the preliminary data that exist regarding the effects of drugs and behavioral interventions on the development of NIDDM.

3.7.1 Pharmacological Intervention Studies

The limited number of studies examining the impact of hypoglycemic agents in IGT have been of short duration and generally statistically underpowered. An exception to the short duration studies was one by Sartor which randomly assigned patients to placebo or tolbutamide and examined the development of NIDDM during the next 10 years (Sartor, et al., 1980). Although tolbutamide (500 mg t.i.d.) decreased the development of NIDDM, the credibility of the results was undermined by the failure to follow intention-to-treat analysis. The Bedford study did not demonstrate a benefit in decreasing progression to NIDDM with tolbutamide at a dose of 500 mg b.i.d. (Keen, 1982). Phenformin was tested in the Whitehall Study, in a 2 x 2 factorial design with diet; no benefit was

noted over five years of study, although the study was probably underpowered (Jarrett, 1979). The French "BIGPRO" study is currently examining the effects of metformin on insulin resistance, glucose intolerance, and cardiovascular risk factors in participants selected on the basis of increased waist-to-hip ratio as a surrogate for increased insulin resistance. Preliminary results reveal decreased weight and insulin levels with metformin (850 mg twice per day), but no significant difference in lipids or glycemia, between placebo and metformin treatment groups (Rudnichi, et al., 1994). Of note, fasting plasma glucose was significantly decreased in the subset of participants with IGT (Eschwege, personal communication). Recent studies of NIDDM document metformin's efficacy in lowering glycemia without weight gain or significant hypoglycemia (DeFronzo, 1995).

Other pharmacological agents that improve glucose tolerance, including alpha glycosidase inhibitors, thiazolidinediones, and insulin, have not been tested in clinical trials of sufficiently large size or long duration to determine whether they prevent or delay the development of NIDDM in persons with IGT. A preliminary, short-term controlled study of the alpha glycosidase inhibitor acarbose (Chiasson, et al., 1994) demonstrated amelioration of elevated glucose levels in IGT patients. The thiazolodinedione troglitazone has recently been demonstrated to increase insulin sensitivity and decrease fasting and post-challenge glucose levels in obese normoglycemic and IGT patients (Nolan, et al., 1994).

3.7.2 Behavioral Intervention Studies-Diet and Exercise

The observed associations between changed lifestyle and obesity with IGT and NIDDM and with the progression between IGT and NIDDM, noted above, suggest that interventions that ameliorate these risk factors may decrease the development of NIDDM. Dietary interventions, usually in the form of caloric or fat restriction, and programs to increase exercise and/or activity levels, have been demonstrated to improve glucose levels in persons with NIDDM (Doar, et al., 1975; Henry, et al., 1991).

Although, in concert, these data suggest that interventions focused on altering diet, decreasing body mass in obese individuals, and increasing exercise levels should ameliorate the progression from IGT to NIDDM, only a few intervention studies have directly tested this hypothesis. These studies have been of relatively short duration and not uniformly well controlled. One of the most dramatic examples of the effect of weight loss on progression from IGT to NIDDM studied morbidly obese persons with IGT who had gastric bypass surgery (Long, et al., 1994). The development of NIDDM occurred in only 0.15 cases per 100 person-years; however, while this approach reinforces the principle that weight reduction reduces risk, the practical, widespread application of this approach is problematic. The Malmo study demonstrated an approximately 50% reduction in the development of NIDDM in IGT patients who participated in an exercise program over six years (10.6% with NIDDM), compared with a non-randomized comparison group of IGT participants (28.6% with NIDDM) (Eriksson, et al., 1991). A recent uncontrolled study from New Zealand demonstrated that a program aimed at decreasing dietary fat and sugars, increasing fiber, decreasing weight and increasing exercise (one half hour three times per week), improved glucose tolerance. However, three of 22 IGT participants who completed the 2 year program developed NIDDM (6.8% per year) (Bourn, et al., 1994). A recent 6 year controlled clinical trial of exercise and diet revealed a decrease in risk for developing NIDDM in Chinese participants with IGT (Pan, et al., 1994).

Although these studies provide some support to the efficacy of lifestyle interventions to decrease the development of NIDDM, the practical application of such methods over time in populations at risk for NIDDM has not been established. Several studies of diet and weight loss have demonstrated sustained weight loss for as long as two years, but relatively few studies have followed participants for longer. A ten year follow-up of an exercise-based outpatient lifestyle modification program that was conducted in middle class volunteers with NIDDM noted poor compliance (10%) with the formal program at one year; however, activity levels remained higher after participation in the program whether participants continued in the formal program or not (Schneider, et al., 1992). Lifestyle intervention implemented as part of the treatment in hypertension studies has been moderately successful (Stamler , et al 1987; Trials of Hypertension Prevention Collaborative Research Group, 1992; Hypertension Prevention Trial Research Group, 1990), but resulted in lower rates of weight loss among African Americans than Caucasians (Kumanyika, et al., 1991).

3.8 Rationale for the Study

The enormous costs associated with NIDDM, including the morbidity, mortality, human suffering and economic burden of caring for NIDDM and its complications, compounded by the difficulty in treating NIDDM once it is established, make NIDDM a suitable target for prevention. The ability to identify persons who are at relatively high risk to develop NIDDM with a simple test (the oral glucose tolerance test) encourages the initiation

of such a study. Moreover, several therapies are available that have demonstrated efficacy in ameliorating the glucose intolerance of IGT and NIDDM (Nolan, et al., 1994; Valiquett, et al., 1995; UKPDS, 1995; DeFronzo, et al., 1995). Behavioral intervention with diet and exercise and treatment with the biguanide metformin and the thiazolidinedione troglitazone are treatments that are safe, have a low frequency of adverse side effects, and improve the cardiovascular risk profile associated with IGT and NIDDM, in addition to lowering glucose levels. Whether any or all of these interventions will be effective in preventing or delaying the development of NIDDM is unknown. Therefore, in order to maximize the chances of at least one therapy being effective, we have chosen three distinct experimental interventions.

Randomization to the troglitazone pharmacological intervention was suspended on May 27, 1998, and discontinued by the NIDDK on June 3, 1998. The NIDDK, with input from the DPP Data Monitoring Board, decided to discontinue use of troglitazone in the DPP based on liver toxicity with hepatic failure (see section 5.3.1).

Economic analyses examining the costs of the interventions will be performed as part of the DPP. Subsequently, the cost-benefit of preventing diabetes and its complications will be prospectively determined.

3.9 Rationale for Medication Washout

The primary outcome of the DPP is the development of diabetes, diagnosed by annual oral glucose tolerance tests (OGTTs) (Diabetes Prevention Program Research Group, 1999). Study treatments are only suspended briefly for outcome assessments. Specifically, the life-style intervention is not interrupted and medications are only held on the morning of the OGTT (usually for 12-15 hours from the dose taken the evening before). Since metformin proved to be effective in delaying the onset of diabetes, it is important to determine if the effects of metformin on the primary outcome were due simply to its acute pharmacologic actions.

The choice of metformin as one of the DPP interventions was predicated on its proven antihyperglycemic effect in both diabetic and non-diabetic individuals (Bailey, 1992; Strumvoll, et. al., 1995). Metformin is known to inhibit hepatic glucose production, predominantly by inhibiting gluconeogenesis (Bailey, et. al., 1996). There is also evidence that metformin may improve insulin action in muscle. (Bailey, et. al., 1996) A positive effect of metformin on the primary outcome in DPP, namely to decrease the number of participants who converted to diabetes, would presumably be interpreted to result from these biological actions. The possibility exists that the positive effect of metformin in DPP might, at least in part, have resulted from an acute effect of metformin to lower blood glucose, and hence would to that extent be acutely reversible. (No previous studies examining acute vs. chronic effects of metformin have been performed.) This possibility was discussed during the planning stage of DPP in relation to whether OGTT testing in DPP should be conducted on or off metformin treatment. However, since the participants receiving medications were randomly assigned to metformin, troglitazone or placebo, any decision to hold medications before the annual OGTT (or 6 monthly fasting glucose test) would have had to take into account the long half-life of troglitazone. Stopping medications for as long as two to four weeks was felt to be impractical and, therefore, medications were not formally "washed out" prior to OGTTs during the DPP.

During the closeout of the DPP, we will have the opportunity to test whether the acute withdrawal of metformin is associated with a rapid deterioration in glucose tolerance significant enough to abrogate the positive effect of the drug during DPP. Such a result would imply the presence of a cohort of participants receiving metformin treatment whose dysmetabolism is adequately controlled by this agent, but who rapidly deteriorate when treatment is abruptly withdrawn. Such a group might be found among those with IGT who are most susceptible to conversion to diabetes, and whose eventual conversion is delayed by metformin. Presumably, they have severe enough defects in insulin secretion and action such that they would express diabetic hyperglycemia in the absence of metformin administration. Withdrawal of metformin treatment would then lead those who are most susceptible to convert to diabetes acutely, and in a manner that potentially could be reversed by reinstituting metformin treatment. On the other hand, the persistence of a significant difference in the proportion of converters in the metformin group versus the placebo group after a metformin "washout" would imply that the efficacy of metformin in DPP extends beyond its acute antihyperglycemic action. In the latter case, there would be substantial interest in learning more about the durability and possible mechanisms of a chronic effect of metformin and clearly this would be an important topic for future studies.

4. DEFINITION OF OUTCOMES *4-1*
4.1 Primary 4-1
4.2 Secondary 4-1
4.2.1 Glucose and Insulin 4-1
4.2.2 Cardiovascular 4-1
4.2.3 Kidney Function 4-2
4.2.4 Physical Activity, Nutrition, and Body Composition 4-2
4.2.5 Health Related Quality of Life 4-2
4.2.6 Resource Utilization, Costs, Health Utilities, and Effectiveness of Treatments to Prevent NIDDM 4-2
4.2.7 Safety Tests 4-2
4.2.8 Stored Specimens 4-2

4. DEFINITION OF OUTCOMES

4.1 Primary

The DPP primary outcome is progression of oral glucose tolerance test (OGTT) results from impaired glucose tolerance (IGT) at baseline to confirmed diabetes, by ADA criteria. To assess progression to this outcome, an OGTT will be performed routinely on an annual basis under conditions described in the Manual of Operations. If the OGTT result meets ADA criteria for diabetes, the participant will be called back for a repeat OGTT within 6 weeks. In order to minimize the unmasking of participants and investigators to a positive but unconfirmed OGTT result, a subset of participants who do not have OGTT results positive for diabetes will be chosen by the Coordinating Center for repeat OGTT. If two sequential OGTTs performed within 6 weeks of each other are positive for diabetes, the clinic and the participant will be notified of the results and the participant will be considered as having reached the primary outcome. If the second test does not meet ADA criteria for diabetes (unconfirmed status), no such notification will be made and the participant will continue on the assigned treatment.

In addition, as a safety measure, participants will be monitored with a fasting plasma glucose (FPG) semi-annually or at any time symptoms suggestive of decompensated diabetes are noted. If this FPG is $\geq$ 126 mg/dL [7.0 mmol/L], the participant will be called back for a repeat FPG within 6 weeks. If the repeat is also $\geq$ 126 mg/dL [7.0 mmol/L], an OGTT will be performed for data collection purposes to assess insulin secretion and sensitivity, the participant will be considered as having reached the primary outcome, and the participant and treatment team will be informed (see section 7.5.6). Again, to maintain masking, the Coordinating Center will ask for a repeat FPG on a subset of participants with FPG < 126 mg/dL [7.0 mmol/L].

Finally, any participants who develop symptoms consistent with hyperglycemia will be encouraged to contact the clinic as soon as possible so that an FPG can be measured. If the FPG is $\geq$ 126 [7.0 mmol/L], the testing strategy outlined above will be followed.

4.2 Secondary

The DPP secondary outcomes were selected for their importance to the clinical and scientific interpretation of the study. They might help explain the mechanism of the primary outcome results, or shed light on how the interventions affect outcomes such as cardiovascular disease and its risk factors, which is at least as clinically meaningful as the primary outcome. Secondary outcomes may be assessed in the study population as a whole or in subsets of the study population, depending on feasibility, cost and the likelihood of deriving significant results from a subset. Clinical center staff and study participants are notified if a secondary outcome result falls outside a clinically acceptable range for that participant, constituting a concomitant condition. The timing of outcome assessments is described in Section 12.

Following is a brief summary of secondary outcomes that will be measured at specified intervals (see section 12.2) in all participants:

4.2.1 Glucose and Insulin

- **HbA$_{1c}$:** Hemoglobin A$_{1c}$ will be assessed to reflect recent average glycemia, to test its relationship to OGTT results and its utility as an indicator of glucose intolerance for the purposes of diabetes prevention.
- **Insulin and Glucose:** To assess insulin secretion, fasting and 30 minute plasma insulin and glucose and fasting plasma proinsulin will be collected during the OGTT. Fasting insulin will be used as surrogate for insulin sensitivity.

4.2.2 Cardiovascular

- **Electrocardiogram**
- **Cardiovascular Symptom Assessment**
- **Arm Blood Pressure**
- **Ankle/Arm Systolic Blood Pressure**
- **Carotid Ultrasound**

- **Fibrinolysis and Clotting Factors:** Fibrinogen, tissue plasminogen activator activity and C-reactive Protein.
- **Lipoproteins:** Lipid profile (total cholesterol, total triglyceride, HDL-cholesterol and derived LDL-cholesterol), or beta quantification in the setting of hypertriglyceridemia (specifically measuring LDL-cholesterol), LDL particle size, LDL-ApoB, and LDL-cholesterol.

4.2.3 Kidney Function

- **Albumin Excretion:** Urinary albumin and creatinine concentrations for albumin excretion, using a spot collection.

4.2.4 Physical Activity, Nutrition, and Body Composition

- **Physical Measurements:** Height, weight, waist and hip circumferences, sagittal diameter, and skin-fold thickness.
- **Physical Activity:** Two standardized questionnaire assessments will evaluate participants' level of physical activity.
- **Nutrient Intake:** A semi-quantitative food frequency questionnaire.

4.2.5 Health Related Quality of Life

- **Psychosocial**: Beck Anxiety and Depression Inventories and the MOS SF-36, to assess mood and general adjustment and health related quality of life.

4.2.6 Resource Utilization, Costs, Health Utilities, and Effectiveness of Treatments to Prevent NIDDM

- **Quality of Well-Being Scale:** A preference-based measure for overall health that may be used for quality-adjusted life years computations.

4.2.7 Safety Tests

- **Routine Blood Chemistry Testing:** Including complete blood count and liver function tests (only for pharmacological and placebo treated participants).
- **Kidney Function:** Serum creatinine (only for pharmacological and placebo treated participants).
- **Adverse Medical Events and Symptoms:** Queries for major adverse events and symptom histories. Medical records will be gathered in the case of significant intercurrent medical events. Serious adverse events, including death, will be reported immediately.
- **Pregnancy Testing:** As needed, based on symptoms and menstrual history.
- **Interval History**

4.2.8 Stored Specimens

- **Sample storage:** Samples of plasma and blood will be stored for possible future analyses, including genetic typing and other analyses related to NIDDM.

5. STUDY DESIGN *5-1*

5.1 Overall Design 5-1
5.1.1 Group Lifestyle Training 5-1

5.2 Participation Criteria 5-1
5.2.1 Principles Guiding Selection of Criteria 5-1
5.2.1.1 Principles Guiding Selection for the Washout Study 5-1
5.2.2 Inclusion Criteria 5-1
5.2.3 Exclusion Criteria 5-2

5.3 Principles Guiding Selection of Interventions 5-5
5.3.1 Discontinuation of the Troglitazone Pharmacological Intervention 5-5

5.4 Assignment to Treatment Groups 5-6
5.4.1 Stratification 5-6
5.4.2 Randomization Method 5-6

5.5 Timing and Conditions of Outcome Assessment 5-6
5.5.1 Primary Outcome 5-6
5.5.2 Secondary Outcomes 5-6
5.5.3 Washout Study Assessment 5-6

5.6 Level of Masking 5-7
5.6.1 Treatment Groups 5-7
5.6.1.1 Unmasking du to early termination of the masked treatment phase 5-7
5.6.2 Central Laboratory Outcomes 5-7
5.6.2.1 Central Laboratory Outcomes 5-7
5.6.3 Clinical Outcomes 5-7
5.6.3.1 Data Collectors 5-7
5.6.3.1.A Data Collectors - Bridge Period 5-7

5. STUDY DESIGN

5.1 Overall Design

The study is a randomized clinical trial to test the safety and efficacy of each of two interventions designed to prevent NIDDM. Participants with IGT and FPG values of 95 - 125 mg/dL [5.3 - 6.9 mmol/L] will be included; in addition we will emphasize recruitment of individuals with particularly high risk of development of NIDDM including those with obesity, the elderly, women with a history of gestational diabetes, and members of minority groups such as African Americans, Hispanic Americans, Asian and Pacific Island Americans, and Native Americans.

Eligible volunteers will be stratified according to center and assigned to one of the two intervention or control groups during a two and two-thirds year recruitment period. The pharmacological intervention will be double blind and placebo controlled. Masking intensive lifestyle intervention assignment to the participants is not possible and masking the investigators is not practical.

After randomization, participants will have quarterly clinical evaluations with a fasting plasma glucose at semi-annual visits and a 75 gram oral glucose tolerance test at annual visits. All participants will be followed for a minimum of three and one-third years after the close of recruitment resulting in 3 1/3 to 6 years of participant follow-up.

Three thousand participants (one thousand per group) will provide 90% power to detect a 33% reduction in the progression to diabetes, assuming an annual rate of progression to diabetes in the control group of at least 6.5% and a level of significance of 5% (two-sided) with adjustment for pairwise comparison of the three treatment groups.

5.1.1 Group Lifestyle Training

Following early termination of the masked treatment phase and debriefing of the DPP participants, all participants will be offered group implemented lifestyle training (Healthy Lifestyle Plan - HELP) with the same goals for weight-loss and activity as shown in DPP to be effective in reducing the risk of developing diabetes.

5.2 Participation Criteria

5.2.1 Principles Guiding Selection of Criteria

- Ensure selection of individuals with a high risk of progression to NIDDM to provide adequate statistical power within the planned duration of the DPP.
- Exclude individuals with conditions or treatments that would interfere with participation in or completion of the protocol, or that have a confounding effect on the measurement of the primary outcomes of the study.
- Exclude individuals who would be at high risk for adverse effects from the proposed interventions.
- Design criteria that can be measured accurately and efficiently in the setting of a multicenter trial that includes heterogeneous populations.

Detailed criteria and methods are described in the Manual of Operations.

5.2.1.1 Principles Guiding Selection for the Washout Study

All DPP participants who have not developed diabetes and who are randomly assigned to medication therapy (placebo or metformin) will be eligible and invited to join the washout study. Medication treated subjects will be eligible regardless of their level of compliance. The only exclusion criteria will be if potential volunteers have some condition that precludes performance of an OGTT according to the original DPP protocol, e.g. treatment with glucocorticoids.

5.2.2 Inclusion Criteria

A. Ethnicity: All ethnic groups

B. Gender: Men and Women
C. Age
   1. Lower age limit: 25 years
      Recommend focus on ages 35 years and older, except for Native Americans, women with a history of gestational diabetes, or women with polycystic ovary syndrome.
   2. Upper age limit: NONE
      Will only exclude for cause (e.g. diseases, functional limitations detailed below).
D. Impaired glucose tolerance with elevated fasting plasma glucose based on a single 75 gm OGTT
   1. Fasting plasma glucose 95-125 mg/dL (5.3 - 6.9 mmol/L),
      AND
   2. 2-hr plasma glucose 140-199 mg/dL (7.8 - 11.0 mmol/L)
      Individuals who meet only one of these glucose criteria may be rescreened after 6 months. Because of the relative higher rate of progression from impaired glucose tolerance to diabetes in Native Americans and the small size of the population, the glucose requirement for eligibility in the Southwest American Indian Center will be fasting glucose <126 mg/dL and 2-hr. plasma glucose 140-199 mg/dL.
E. Body-mass Index
   1. Lower BMI limit: 24 kg/m^2
      Because Asian-Americans develop diabetes and IGT at a BMI that is lower than the BMI of the general U.S. population with IGT and NIDDM, the BMI criteria for eligibility for Asian Americans will be 22 kg/m^2 or greater.
   2. Upper BMI limit: NONE

5.2.3 Exclusion Criteria

A. Exclusion for underlying disease likely to limit life span and/or increase risk of interventions
   1. Cancer requiring treatment in the past 5 years, with the exception of cancers which have been cured or, in the opinion of the investigator, carry a good prognosis.
      For example, non-melanoma skin cancer, papillary thyroid carcinoma, and cervical carcinoma in situ.
   2. Infectious diseases
      a. Self-reported HIV positivity
         No serologic testing
      b. Active tuberculosis
   3. Cardiovascular disease
      a. Hospitalization for treatment of heart disease in the past 6 months
         Includes therapeutic procedures (e.g. CABG, PTCA), not diagnostic procedures (e.g. coronary angiogram).
      b. New York Heart Association Functional Class > 2
      c. Left bundle branch block on ECG
      d. Third degree atrioventricular block on ECG
      e. Uncontrolled hypertension: SBP >180 mmHg or DBP >105 mmHg on treatment
         Milder degrees of hypertension would prompt referral to primary care provider for treatment (with DPP compatible agent) but would not exclude.
      f. Stroke or transient ischemic attack in the past 6 months
      g. New York Heart Association Functional Class 2 in persons who are currently treated with a loop diuretic or digitalis preparation
   4. Gastrointestinal disease
      a. Self-reported chronic hepatitis or cirrhosis, or serum AST or ALT elevated by the following criteria:
         serum AST ≥ 66 U/L
         serum ALT ≥ 58 U/L if over 47 years
         serum ALT ≥ 118 U/L if male ≤ 47 years
         serum ALT ≥ 46 U/L if female ≤ 47 years
      b. Episode of alcoholic hepatitis or alcoholic pancreatitis ever
      c. Inflammatory bowel disease requiring treatment in the past year
      d. Recent or significant abdominal surgery (e.g. gastrectomy)

Would not exclude surgery for conditions with no long-term adverse effects (e.g. appendectomy, cholecystectomy, polypectomy).

5. Renal disease
   a. Serum creatinine ≥1.4 mg/dL (124 μmol/L) for men; ≥1.3 mg/dL (115 μmol/L) for women
   b. Urine protein ≥2+ on one occasion (dipstick), in the absence of infection or vaginal contamination
   c. In individuals who are or will become 80 years of age during the study, a direct measure of creatinine clearance, based on a 24 hour urine collection, will be required. Creatinine clearance levels ≥ 75 mL/min will be required in order for these individuals to be eligible.
6. Lung disease
   a. Chronic obstructive airways disease or asthma requiring daily therapy
   b. New York Heart Association Functional Class > 2
   c. Use of oxygen at home
7. Electrolyte abnormality
   Serum Potassium <3.2 or >5.5 mmol/L
   Serum sodium and bicarbonate levels are reported to the clinical center during screening to alert investigators to participants with electrolyte or acid-base levels outside expected ranges.
8. Anemia
   Hematocrit < 36.0% in men or <33.0% in women
9. Other chronic disease or condition likely to limit life span to < 6 years.
10. Conditions not specifically mentioned above may serve as criteria for exclusion at the discretion of the clinic site.

B. Exclusion for conditions or behaviors likely to effect the conduct of the DPP
1. Unable or unwilling to give informed consent
2. Unable to communicate with the pertinent clinic staff
3. Another household member is a participant or staff member in DPP
4. Unwilling to accept treatment assignment by randomization
5. Current or anticipated participation in another intervention research project that would interfere with any of the interventions offered in DPP
6. Weight loss of > 10% in past 6 months for any reason except post-partum weight loss
7. Likely to move away from participating clinics in next 5 years
8. Unable to walk 0.25 mile in 10 minutes
9. Unable to complete remainder of DPP run-in tasks
10. Pregnancy and childbearing
    None of these criteria are necessarily permanent. Women may be considered potentially eligible after sufficient time has elapsed or after other relevant conditions have changed.
    a. Currently pregnant or less than 3 months post-partum
    b. Currently nursing or within 6 weeks of having completed nursing
    c. Pregnancy anticipated during study
       Recognizing the difficulty of foreseeing events this far in the future, women who answer "probably not" would be included. Women with definite plans to become pregnant would be excluded.
    d. Unwilling to undergo pregnancy testing or to report possible or confirmed pregnancies promptly during the course of the DPP
    e. Unwilling to take adequate contraceptive measures, if potentially fertile
       Clinic sites would determine how far to probe regarding type of method, conduct of method, and likelihood of fertility (medical history, level and type of sexual activity).
11. Major psychiatric disorder which, in opinion of clinic staff, would impede conduct of the DPP
    Concern regarding such a disorder would be prompted initially either by clinic staff perceptions or by self-report of psychiatric hospitalization. Confirmatory evidence could include scores on standardized instruments (e.g. Brief Symptom Inventory or Mini-mental status examination) or clinical evaluation.
12. Excessive alcohol intake, either acute or chronic
    Defined as any one of the following: 1) average consumption of 3 or more alcohol containing beverages daily; 2) consumption of 7 or more alcoholic beverages within a 24 hr period in the past 12 months; or 3) clinical assessment of alcohol dependence based on two or more positive responses to the CAGE questionnaire (if confirmed by further probing) or on other evidence available to clinic staff. If any of these exclusion criteria are met, the subject may still be considered eligible if, after an explanation of

the importance of limiting alcohol intake during the DPP, the clinic staff believes that the volunteer can and will limit future alcohol intake to acceptable levels.

13. Other condition which, in opinion of clinic staff, would effect the conduct of the DPP

C. Exclusions related to metabolism
   1. Diabetes at baseline evaluation evidenced by any of the following:
      a. Fasting plasma glucose ≥ 126 mg/dL [7.0 mmol/L]
      b. 2-hour plasma glucose ≥ 200 mg/dL [11.1 mmol/L] based on 75 gm OGTT
      c. Diabetes diagnosed by a physician and confirmed by other clinical data (e.g. documentation of a fasting plasma glucose ≥ 126 mg/dL [7.0 mmol/L] or a positive OGTT)

      Persons who report having been diagnosed with diabetes but who have neither taken hypoglycemic medication nor have confirmatory clinical evidence of diabetes may undergo OGTT at the discretion of the clinic site.
      d. Ever used hypoglycemic medication (oral agents or insulin; except during GDM)
   2. Disease associated with disordered glucose metabolism
      a. Cushing's Syndrome
      b. Acromegaly
      c. Pheochromocytoma currently under treatment (i.e. not surgically cured)
      d. Chronic pancreatitis
   3. Thyroid disease, suboptimally treated

      Suboptimal treatment is defined as a sensitive TSH assay outside the clinically acceptable range (except for low TSH levels in patients being treated with suppressive doses because of a history of thyroid nodules or thyroid cancer). TSH would be assessed in these settings: 1) reported use of anti-thyroid medication, 2) reported use of thyroid hormone.
   4. Fasting plasma triglyceride level > 600 mg/dL (6.77 mmol/L) on one occasion despite treatment
   5. Conditions not specifically mentioned above may serve as criteria for exclusion at the discretion of the clinic site

D. Exclusions related to medications
   1. Antihypertensives
      a. Thiazide diuretics
      b. Beta-blockers
   2. Lipid-lowering agents - Niacin only

      Statins, bile acid sequestrants, and gemfibrozil would not be excluded.
   3. Glucocorticoids other than topical, ophthalmic, and inhaled preparations
   4. Antibiotics
      a. HIV-related agents, e.g. AZT, DDI, pentamadine
      b. Antituberculous agents, e.g. INH, ethambutol (except INH alone as prophylaxis)
   5. Antineoplastic agents
   6. Psychoactive agents
      a. Antipsychotic agents

         These include, but are not limited to: Haldol, Loxitane, Mellaril, Navane, Prolixin, Stelazine, Thorazine, and Trilafon.
      b. Fluoxetine (Prozac) at doses exceeding 20 mg daily, or other selective serotonin reuptake inhibitors (SSRI) at comparable doses.

         Low dose fluoxetine and other antidepressant medications will not be excluded.
   7. Bronchodilators
      a. Aminophylline, if used daily
      b. Inhaled beta-agonists, if used daily
   8. Other medications
      a. Phenytoin
      b. Amphetamines
      c. Prescription weight-loss drugs
   9. Medications not specifically mentioned above may serve as criteria for exclusion at the discretion of the clinic site

5.3 Principles Guiding Selection of Interventions

The interventions considered for the DPP included all available therapies that had demonstrated efficacy in improving the abnormalities in glucose metabolism that characterize IGT and NIDDM by decreasing fasting and/or post-challenge glucose levels. The available modalities were scrutinized with regard to the following additional criteria: strength and consistency of data supporting efficacy, safety, and tolerability including side-effect profile at the doses required for efficacy; potential acceptance by participants; potential for long-term adherence; ability to translate to a non-research setting; other desirable effects such as ability to improve cardiovascular risk factors; and ability to mask the intervention (availability of placebo). No single intervention fulfilled all of the criteria above. However, three interventions were considered to have the requisite qualities necessary for the DPP.

An intensive lifestyle intervention, focusing on dietary changes to promote weight loss and on increased physical activity, was selected because of the recognized short-term salutary effects of such a regimen on glucose metabolism, dyslipidemia, and other cardiovascular risk factors. The scientific literature (reviewed previously) provides support for the hypothesis that decreases in body weight and increases in physical activity ameliorate hyperglycemia and may prevent or delay the development of NIDDM. In addition, decreasing waist-to-hip ratio may decrease the risk of NIDDM; however, modification of waist-to-hip ratio is best accomplished by losing weight and increasing physical activity. In addition, the safety profile and low frequency of adverse side effects complemented the appeal of a nonpharmacological intervention. Although other features of intensive lifestyle intervention, such as the inability to mask participants and investigators from knowing treatment assignment, did not fulfill the criteria, the positive attributes were considered to outweigh any shortcomings.

Two pharmacological interventions were selected. The biguanide metformin was selected on the basis of its demonstrated efficacy to improve glucose intolerance (UKPDS, 1995; DeFronzo, et al., 1995) and the expectation that it would prevent or delay progression of IGT to NIDDM. In addition, use of metformin was supported by its safety profile, the low frequency of adverse effects at the doses necessary to obtain the desired metabolic effect, the strength and consistency of data supporting efficacy, potential for long-term adherence, ability to translate in the non-research setting, other desirable effects such as the ability to improve cardiovascular risk factors, and the availability of placebo. Troglitazone, a member of a new class of thiazolidinedione drugs termed insulin sensitizers, was selected on the basis of its potent effect to lower glycemia in NIDDM patients without an increase in insulin levels, its ability to improve lipid levels, the absence of significant side-effects or adverse events in short-term (1-2 year) human studies, the potential for translation in the non-research setting, and the availability of placebo (Nolan, et al., 1994; Valiquett, et al., 1995). Sulfonylureas, even in the relatively low doses that have been used in past studies of prevention, are associated with hypoglycemia. Because of this potential side-effect, sulfonylureas were not considered further for use in the DPP.

5.3.1 Discontinuation of the Troglitazone Pharmacological Intervention

Randomization to the troglitazone pharmacological intervention was suspended on May 27, 1998, and discontinued by the NIDDK on June 3, 1998. The NIDDK, with input from the DPP Data Monitoring Board, decided to discontinue use of troglitazone in the DPP based on the following: "Associated with troglitazone use in the DPP, there is an increased risk of liver toxicity resulting in serum ALT levels greater than or equal to 8 times the upper limit of normal. In the DPP, there has been one case of hepatic failure requiring liver transplantation. Within the context of this research trial, safety monitoring, even if intensified, is not likely to eliminate the risk. It is also too early in the trial to estimate reliably and compare the absolute risk or benefit of continuing troglitazone in this study population. We are not willing to continue to study a drug for the purpose and benefit of preventing progression from impaired glucose tolerance to diabetes when the drug has demonstrated liver toxicity with hepatic failure."

Participants randomized to troglitazone prior to May 27, 1998, were unmasked to their intervention assignment, monitored to ensure that no liver toxicity developed after discontinuing the troglitazone medication and continue follow-up by the DPP Research Group (see the DPP substudy protocol "Follow-up of DPP Participants Randomized to Troglitazone"). Pharmacological participants randomized to metformin or double-placebo, discontinued their troglitazone-placebo, continue their coded metformin medication and remained masked to their pharmacological assignment (metformin or metformin-placebo).

5.4 Assignment to Treatment Groups

5.4.1 Stratification

Randomization will be stratified by clinical center. This will ensure balance between the three treatment groups with respect to anticipated differences in the participant populations and possible differences in participant management.

5.4.2 Randomization Method

There are several alternative methods to assign the participants randomly within clinical center (e.g., simple randomization or permuted block designs). The urn method of randomization provides a high probability of balance in treatment assignments, is unpredictable in unmasked studies, and allows an explicit randomization analysis to be conducted with relative ease (Wei and Lachin, 1988). For these reasons, the urn method will be used to randomly assign participants to the three treatment groups.

A sequence of randomization numbers within a clinical center will be constructed of the form XXYZZZ, where XX is the clinical center number, Y is a number that indicates assignment to either the intensive lifestyle intervention or pharmacological treatment, and ZZZ is a three digit sequence number within each XXY combination. The DPP Coordinating Center will prepare the master randomization list with assignments to the three treatment groups within a clinical center using the standard urn design.

The sequence of pharmacological randomization numbers within a clinical center with the specific pharmacological treatment assignment (i.e., metformin or placebo) will be forwarded, in confidence, to the drug distribution center for drug labeling and distribution. Pharmacological treatment assignment to the sequence of pharmacological randomization numbers will be known only by the staff of the DPP Coordinating Center and the drug distribution center.

5.5 Timing and Conditions of Outcome Assessment

5.5.1 Primary Outcome

The primary outcome, progression from impaired glucose tolerance (IGT) to diabetes, is assessed by OGTT testing annually, by fasting plasma glucose (FPG) every six months, and at the time that symptoms consistent with hyperglycemia occur. Conditions for the OGTT are specified in the Manual of Operations. The annual OGTT and 6 month FPG will be postponed for up to six weeks if a temporary concomitant condition exists that would affect glucose tolerance. An OGTT that is positive for diabetes, or a six-monthly FPG that is ≥ 126 mg/dL [7.0 mmol/L], will be repeated for confirmation before the participant is considered to have reached the primary outcome. When a participant has been in a "time-out" (other than pregnancy), such as for a concomitant disease known to affect glucose tolerance, the primary outcome will be assessed at the time of the next regularly scheduled six-monthly FPG or annual OGTT after that time-out ends.

5.5.2 Secondary Outcomes

Secondary outcomes will be assessed according to the Section 12 schedule.

5.5.3 Washout Study Assessment

With the announcement of the DPP results in August, 2001, each DPP site will communicate with their participants at a general meeting, and by telephone and through the mail in order to initiate the debriefing process. Medication participants who agree to participate in the washout study will be asked to continue their medications until approximately 7 days prior to a post-washout OGTT to be performed within approximately the next 6 weeks. The nature and purpose of the washout study will be explained to them and informed consent will be obtained. DPP medication group subjects who agree to participate in the washout project will agree not to be told of their previous DPP assignment (metformin vs. placebo) until after completion of the washout OGTTs. Participants found to have converted to diabetes will require a confirmatory OGTT (still off study medication), as was performed during the DPP.

5.6 Level of Masking

5.6.1 Treatment Groups

Pharmacological treatment assignment (metformin or placebo) will be double masked. Masking participants to the intensive lifestyle intervention versus pharmacological treatment is not possible and masking the investigators is not practical. At no time will the code of the specific pharmacological treatment assignment be broken without the express knowledge and consent of the clinical center's principal investigator. The exception to this policy is that if a woman becomes pregnant while on study medication, the medication will be discontinued and she, her PI, and her Primary Care Provider will be unmasked. Information on the potential teratogenicity of the medication will be provided both to the participant and her care providers.

5.6.1.1 Unmasking du to early termination of the masked treatment phase

Pharmacologic treatment assignment, previously double masked, will be unmasked starting August 2001. Participants previously assigned to placebo will discontinue their study medications.

5.6.2 Central Laboratory Outcomes

Primary outcome data (OGTT and FPG results) measured centrally will remain masked to the investigators and to the participants until confirmed progression from IGT to diabetes. Plasma lipid levels and HbA_{1c} measured centrally will remain masked to the investigators and to the participants during the study. Two exceptions to this policy are: 1) if an individual result falls outside a pre-determined, clinically acceptable range representing a significant risk to the participant, it will be confirmed. If the result remains abnormal, the investigator and the participant will be notified and appropriate clinical steps will be taken. 2) If an individual participant insists on knowing specific secondary outcome results, such as plasma lipid values, and their continued participation in DPP is considered by the investigator to be in jeopardy, the investigator may request an exception from the Coordinating Center.

5.6.2.1 Central Laboratory Outcomes

Outcome tests will not be masked during the bridge period, with the exception of temporary masking of oral glucose tolerance and fasting test results that require a repeat test for confirmation of diabetes, and a sample of normal tests to be repeated to minimize the amount of behavior change that occurs between tests.

5.6.3 Clinical Outcomes

Some of the secondary outcome data, such as blood pressure, weight, and waist circumference will be measured at the clinical center. It is not feasible to keep these data masked from the participants, if they want to know their result. Therefore, participants will be unmasked to measurements of body size and blood pressure.

5.6.3.1 Data Collectors

In order to promote objectivity of data collection and to minimize the opportunity for bias, the intent is to separate outcome measurement from the intensive lifestyle intervention case managers. This is particularly important for dietary intake data, blood pressure, interview questionnaires, and anthropomorphic measures, where the potential exists for subjectivity. Intensive lifestyle intervention case managers must not perform these outcome measures for participants with whom they are intervening.

5.6.3.1.A Data Collectors - Bridge Period

Since the masked treatment phase of DPP is completed and all subjects and staff are unmasked, clinics may decide, based on staffing requirements, the proper certified person(s) to do outcome assessments.

6. ENROLLMENT OF PARTICIPANTS ... 6-1

6.1 Recruitment: Goals and Strategy ... 6-1

6.1.1 Publicity and Participant Contacts ... 6-1

6.2 Informed Consent and Staged Screening ... 6-1

6.2.1 Informed Consent Policy ... 6-1

6.2.2 Staged Screening Policy ... 6-1

6.2.3 Screening and Informed Consent Procedures ... 6-2

6. ENROLLMENT OF PARTICIPANTS

6.1 Recruitment: Goals and Strategy

The target cohort for DPP is approximately 50% minorities (African Americans, Hispanic Americans, Native Americans, and Asian and Pacific Island Americans) and at least 50% women. In addition, approximately 20% of the target cohort is older Americans, of age 65 years and older.

The Recruitment Coordinator, with approval of the Program Coordinator and the Principal Investigator, will develop a clinic specific recruitment strategy. All centers will be represented at an initial 4-5 day series of training sessions before recruitment is initiated. Additional training sessions will be held during the three years of participant recruitment. In addition to education about sophisticated technical and interpersonal skills necessary for recruitment, the goal of these sessions is for the Recruitment Coordinators to form a dynamic system of support for problem solving and development of DPP-specific recruitment techniques.

6.1.1 Publicity and Participant Contacts

Centrally prepared publicity and recruitment procedures and tools that address the target cohort in a culturally sensitive manner include print and radio public service announcements, participant contact brochures, flyers, fact sheets, and posters. Publicity and recruitment efforts are primarily the responsibility of the local Recruitment Coordinators. Where appropriate, clinics are urged to form Advisory Boards and hold community meetings to support and assist in formulating the message and methods for publicity and participant recruitment. A variety of methods will be used to contact community members including, for example, work-site mailings and publicity, hospital record reviews, mass mailings, and PSAs.

A national press conference and kickoff for recruitment will support the credibility of local initiatives. Statements of support will be sought from national interest groups such as the ADA and organizations that represent minority interests. These will provide a framework for clinic recruitment staff to contact the local affiliates of these organizations for similar endorsements.

6.2 Informed Consent and Staged Screening

6.2.1 Informed Consent Policy

The DPP informed consent process is staged, in order to maximize potential volunteer's understanding of information required for an informed decision regarding participation, including their personal risks and benefits, and to promote efficiency.

This process is designed to meet the ethical obligations to the participant and improve retention by fostering a progressively increasing understanding of the DPP by the participant as well as the development of a positive relationship between the volunteer and clinic staff. It is an interactive and conversational process, the ultimate goal being maximum understanding of DPP and its impact upon the volunteer's life. This understanding includes what the responsibility of the participant is to the DPP and the responsibility of the investigators to the participant. It is anticipated that one result of this process is maximized retention of participants in DPP. This informed consent process is integrated with the screening and enrollment process.

Each stage in the Informed Consent process includes both verbal and written descriptions of relevant information, and discussion including opportunities for questions to be addressed. Each stage allows the participant to make a decision whether to proceed to the next screening step. Early steps include a description of the Informed Consent and Screening Process and the ultimate goal of the process. The participant views a video presentation during which DPP investigators are present to answer any questions. After the presentation and discussion of this information the participant is asked to sign the consent form relevant to that stage. Prior to presentation of the final two consent forms, a "volunteer understanding questionnaire" is answered by the participant in order to assure that the participant understands DPP.

6.2.2 Staged Screening Policy

The staged screening process is intended to accomplish the following:

- Identify potentially eligible volunteers for the DPP;
- Verify eligibility of potential participants;

- Accomplish the objectives of the informed consent process;
- Refer persons diagnosed with NIDDM during screening to clinical care;
- Complete baseline procedures;
- Randomize volunteers into the DPP.

Overview

The staged screening process is a series of five steps that will identify high risk volunteers with impaired glucose tolerance (IGT) who are potentially eligible for the DPP. Each sequential step will identify a subset of high-risk individuals who meet increasingly restrictive eligibility criteria. During the final steps, participants will be tested for remaining eligibility criteria, complete a run-in period to explore their ability to participate, and sign a final informed consent for randomization. The intent is to allow participating centers some flexibility in approach while maintaining a standard set of baseline procedure entry criteria. This flexibility includes the possibility to combine certain steps, as noted below. Those that cannot be combined are: Steps 2 and 3 because of the delay until the central laboratory OGTT values are available, and Steps 3 and 4 because of the mandatory 3-week Run-in interval. Complete definitions and procedures can be found in the Manual of Operations.

6.2.3 Screening and Informed Consent Procedures

A recruitment strategy based upon local needs and resources is established by each clinical center. Section 6.1 describes goals, strategy, and publicity that usually provides the first information about the study to potential participants.

Initial Contact (Step 1)

Interested persons contact the clinical center by phone or in person, and are given basic information about the study. If they appear eligible based on a brief medical history, they are invited to have a screening glucose level drawn after informed consent is obtained.

Clinical centers have the option of conducting the screening glucose step in several ways: a capillary test without regard to meals (random glucose), a fasting capillary or plasma test, or both in sequence, depending on logistics at the center. The results of tests are evaluated using appropriate criteria to determine if they are in the target range. Persons with results in the target range are given more information about the DPP and those who give informed consent will continue through the screening process and are scheduled for an oral glucose tolerance test (OGTT).

Oral Glucose Tolerance Test - OGTT (Step 2)

Fasting laboratory tests and a two-hour 75 gram OGTT is performed to diagnose IGT utilizing the criteria developed by the American Diabetes Association (ADA Study Group, 1997). This criteria has been modified to include a fasting plasma glucose between 95-125 mg/dL in addition to a two hour value of 140-199 mg/dL in order to identify participants who are at higher risk for progression to NIDDM. A video presentation provides additional information about the study.

OGTT samples are centrally analyzed by the DPP Central Biochemistry Laboratory (CBL) for eligibility criteria. Fasting glucose readings will be analyzed locally and compared to appropriate criteria to determine if the glucose tolerance test should be continued. Centers have the option of stopping the OGTT when the fasting sample does not meet the criteria, or completing the test to screen for non-eligible disorders of glucose tolerance (i.e., diabetes, non-eligible IGT). Participants are informed of their results within ranges: normal (not eligible), elevated (not eligible and either: IGT but fasting level below criteria; or requiring further workup for possible diabetes), and eligible. Specific glucose values are furnished only to those who are ineligible in order to maintain the masking of outcomes. Persons with IGT who do not meet DPP criteria will be notified and are eligible for rescreening in 6 months.

Those who meet OGTT eligibility criteria are invited to continue the screening process. The participant's family is encouraged to participate in the decision by attending (at least) the next step. Additional screening and baseline bloods may be drawn at this visit, or may be drawn at the next visit, at clinic discretion.

Initiate Run-in (Step 3 - Start)

Prior to the run-in, the participant receives additional information about DPP, including further reviews of the research interventions, randomization, masking, test procedures, risks and benefits, and informed consent is

obtained. The 3 week run-in period is designed to allow participants to make an informed decision about participation in the study, based on completing tasks similar to those required of participants (taking placebo pills on the correct schedule, completing diet and activity diaries and keeping appointments). It will also allow staff to determine a participant's suitability for the DPP. During this time, additional information about the DPP is provided and a volunteer understanding questionnaire is administered to confirm understanding. Additional eligibility screening and baseline tests, including a history and physical examination, are completed during this time.

Run-in Follow-up (Step 3 - End)

At this visit performance of the run-in period is evaluated. Eligibility requires satisfactory completion of the placebo pill taking, and other assigned tasks. If participants do not successfully complete the run-in, but the investigator and staff at the center feel that a second run-in period would prove useful, the run-in may be repeated.

Following successful completion of the run-in phase, all eligibility criteria are reviewed and baseline procedures are completed. Participants will be scheduled for carotid ultrasound evaluation before or at the randomization visit (Step 4, prior to actual randomization).

Randomization Visit (Step 4)

Final informed consent will be reviewed in detail. If the participant is eligible and signs the informed consent, baseline laboratory samples are collected and the participant is randomly assigned to a treatment group at this visit and begins meetings with appropriate DPP staff.

7. PARTICIPANT MANAGEMENT PROTOCOLS *7-2*

7.1 Schedule of Follow-up Visits 7-2
7.1.1 Interim Visits 7-2
7.1.2 Suspension of Follow-up Visits 7-2
7.1.3 Schedule of Follow-up visits during the Bridge Period 7-2

7.2 Standard Lifestyle Recommendations 7-2

7.3 Intensive Lifestyle Intervention 7-3
7.3.1 Description of Program 7-3
7.3.1.1 Changes to the Intensive Lifestyle Program during the Bridge Period 7-5
7.3.2 Indices of Adherence 7-5
7.3.3 Healthy Lifestyle Plan - HELP 7-5

7.4 Pharmacological Treatment 7-5
7.4.1 Description 7-5
7.4.1.1 Metformin 7-6
7.4.2 Dosing Schedule 7-6
7.4.2.1 Pre-randomization 7-6
7.4.2.2 Post-Randomization - Steps I and II 7-6
7.4.2.3 Unmasking and open-label follow-up 7-7
7.4.2.4 Metformin Unmasking and Open Label Follow-up During the Bridge Period 7-7
7.4.3 Restarts and Titration Due to Potential Non-Gastrointestinal Side Effects 7-7
7.4.4 Safety Monitoring and Measures to Reduce and Manage Potentially Drug Related Side Effects 7-7
7.4.4.1.A Laboratory Safety Monitoring During the Bridge Period 7-10
7.4.5 Indices of Adherence 7-10
7.4.5.1 Indices of Adherence for the Bridge Period 7-10

7.5 Definition and Management of Concomitant Conditions 7-11
7.5.1 Hypertension 7-11
7.5.2 Lipids 7-12
7.5.2.1 Dietary Treatment 7-12
7.5.2.2 Drug Treatment 7-12
7.5.2.3 Treatment Protocols 7-13
7.5.3 Psychological Diseases and Use of Psychoactive Agents 7-13
7.5.4 Pregnancy 7-13
7.5.4.1 Indications During Pregnancy 7-14
7.5.4.2 Safety Monitoring 7-14
7.4.5.2.A Pregnancy Monitoring During the Bridge Period 7-14
7.5.4.3 DPP Interventions During Pregnancy 7-14
7.5.4.4 DPP Interventions During Breast Feeding 7-14
7.5.4.5 Outcome Assessment Following Pregnancy 7-15
7.5.5 Smoking 7-15
7.5.6 NIDDM 7-15
7.5.6.1 Diabetes Discovered At Screening 7-15
7.5.6.2 Interim Visits For Symptoms 7-15
7.5.6.3 Intervention and Follow-up for Participants with Diabetes and Fasting Glucose <140 mg/dL 7-16
7.5.6.3.A Intervention and Follow-up for the Participants with Diabetes and Fasting Glucose < 140mg/dL 7-16
7.5.6.4 Intervention and Follow-up for Participants with Diabetes and Fasting Glucose ≥ 140 mg/dL 7-16
7.5.6.4.A Intervention and Follow-up for Participants with Diabetes and Fasting Glucose ≥ 140 mg/dL.. 7-16
7.5.7 Cardiovascular Disease 7-17

7.6 Retention Monitoring and Recovery of Inactive Participants 7-18

7.6.1 Steps to Maximize Adherence and Retention 7-19
7.6.1.1 Social Support 7-19
7.6.1.2 Removal of Barriers to Participation 7-19
7.6.1.3 Honoraria 7-19
7.6.2 Monitoring Retention 7-19
7.6.2.1 Visit Monitoring 7-20
7.6.2.2 Measures of Stress and Social Support 7-20

7. PARTICIPANT MANAGEMENT PROTOCOLS

Each participant is randomized to one of three treatment groups:

1. Intensive lifestyle intervention.
1. Metformin with standard lifestyle recommendations.
1. A metformin placebo with standard lifestyle recommendations.

7.1 Schedule of Follow-up Visits

Follow-up visits will be scheduled at 3 month intervals throughout the duration of the DPP. Participants assigned to the intensive lifestyle intervention will have more frequent scheduled follow-up visits to implement the program of weight reduction and increased calorie expenditure. Participants assigned to the two pharmacological treatments (metformin or placebo) will be scheduled for one visit one month after randomization for dose titration. All randomized participants will continue their scheduled follow-up visits for the duration of DPP regardless of their level of compliance with the assigned treatment. Outcome and safety assessments will be conducted according to the schedule in section 12.2.

7.1.1 Interim Visits

An interim visit refers to all visits other than scheduled follow-up visits (i.e., standard or major follow-up visits). Interim visits may be required for the monitoring or management of an emerging or existing condition, or to repeat procedures which, at a previous visit, were found to be deficient. Such visits may be held as frequently as deemed necessary.

7.1.2 Suspension of Follow-up Visits

The occurrence or presence of the following will constitute inactive follow-up and suspension of the scheduled follow-up protocol:

- Voluntary withdrawal by the participant, or
- Condition which, in the opinion of the principal investigator, makes it unsafe for the participant to continue.

Efforts to return participants to an active status will be made regularly, as appropriate.

7.1.3 Schedule of Follow-up visits during the Bridge Period

Mid-year and annual individual visits will take place in person, and quarterly follow-up visits can be completed in person or by phone. All participants are offered group lifestyle sessions as part of HELP.

7.2 Standard Lifestyle Recommendations

A. After randomization, all participants will receive the following standard lifestyle recommendations.
At the first visit, the staff will spend approximately 20 - 30 minutes with each participant individually, reviewing the importance of a healthy lifestyle for the prevention of NIDDM, and for prevention and treatment of other diseases (such as cardiovascular disease and hypertension). Materials, provided by the Lifestyle Resource Core, will be given to each participant and the staff will review the basic information in the materials with the participant. The materials will include information or recommendations on:

- Healthy Eating - The Food Pyramid is used as the basis of discussion. Participants are encouraged to consume the equivalent of a NCEP Step I diet.
- Healthy Weight - The materials include a weight chart, a description of how to measure their own waist, and guidelines for appropriate waist circumferences. The materials include discussion of the benefits of modest weight reduction (5-10% of initial weight) for those who are overweight and describe general behavior changes that can be made to produce weight loss. Changes in both dietary intake and exercise are recommended for weight loss and maintenance.
- Physical Activity - Participants are encouraged to adopt and maintain a more active lifestyle. The materials suggest ways to increase general activity (such as using stairs instead of elevators) and provide a chart with information regarding the number of calories used in 10 minutes of various forms of exercise. Participants are encouraged to increase their activity gradually and to try to reach the goal of at least 30 minutes of physical activity (such as walking or biking) on 5 days each week.

- Smoking - All participants who smoke are strongly encouraged to stop smoking. The materials include general information about the health risks associated with smoking, the benefits and specific strategies for stopping smoking, and a list of programs available to participants to help them stop smoking. Participants are encouraged to read the materials carefully, to bring any questions they have to the next visit, and to try to gradually work toward a healthier lifestyle.
- Alcohol Intake - Participants, especially those in the pharmacological treatment groups due to the nature of the medication used in the DPP, are informed of the need to avoid excessive alcohol intake (see section 5.2.3) and binge drinking.

B. At the end-month 3 follow-up visit, the staff briefly review the materials with the participant. The staff answer any questions regarding the information provided.

C. At yearly intervals (soon after each annual assessment), the staff meet with each participant to review the information on diet, exercise, weight and smoking.

7.3 Intensive Lifestyle Intervention

The goals of therapy for participants randomly assigned to the intensive lifestyle intervention are two:

- Achieve a weight reduction of at least 7% of initial weight and maintain this weight reduction throughout the DPP.
- Achieve at least 150 min/week of moderate intensity exercise (such as walking and bicycling), and maintain this level of physical activity throughout the DPP.

Recognizing that it is very difficult to produce long term changes in eating and exercise behaviors and in body weight, the intensive lifestyle intervention is designed to maximize the chances of achieving and maintaining these goals. The intensive lifestyle intervention is based on the premise that long-term changes in diet and exercise and sustained motivation to maintain behavior changes are most likely to occur in an intensive intervention that includes the following:

- Training in diet, exercise, and behavior modification skills
- On-going contact with participants, and continued, frequent (no less than monthly) support for behavior change
- Diet and exercise interventions that are flexible and sensitive to cultural differences and acceptable in the specific communities in which they are implemented
- A combination of individual and group intervention
- A combination of a structured protocol (in which all participants receive certain common information) and the flexibility to tailor strategies individually to help a specific participant achieve and maintain the study goals
- Emphasis on self-esteem, empowerment, and social support

7.3.1 Description of Program

A. *Lifestyle Resource Core.*
The Lifestyle Resource Core, located at the University of Pittsburgh, developed the materials for the intensive lifestyle intervention and will provide on-going training and support for the case managers.

B. *Staff for Intensive Lifestyle Intervention.*
Case managers at each clinical center will carry out the intensive lifestyle intervention. Case managers will be individuals with experience in nutrition, exercise, and/or behavior modification and will receive further training from the Lifestyle Resource Core. The case managers are supported by staff at each center with additional expertise in nutrition, exercise, or behavior modification. Each participant randomized to the intensive lifestyle intervention is assigned a case manager who will work with him or her throughout the DPP (changing case managers for a specific participant may be necessary to help retain participants in the DPP or to help participants achieve the weight and exercise goals).
Other staff (e.g. peer counselors and exercise leaders) are employed as appropriate at each center.

C. *Format of Intervention.*
1. 16-Session Core Curriculum.
Participants randomized to the intensive lifestyle intervention will participate in a 16-session core curriculum. This initial intervention will commence as soon as possible after randomization, and must begin within 2-3 months after randomization. These 16 sessions are to be held over 24 weeks. These initial 16 sessions will be individual sessions involving the participant and case manager; however, small

groups may be formed if all members of a small group start and complete the 16 sessions together (closed group format). In addition, a spouse or other key family member may be invited to attend. The first 8 sessions and four of the following 8 sessions must be with the case manager; the other 4 or more sessions may be with the case manager, a peer-counselor, home visitor, or the equivalent. Topics and lesson materials to be utilized in this 16 session core curriculum have been developed by the Lifestyle Resource Core and are in the Lifestyle Intervention Manual. Topics included in this 16 sessions core curriculum include:

a. Lifestyle change as a way to prevent diabetes: the importance of healthier eating and exercise behavior.
b. Looking at what you eat - self-monitoring of eating behavior.
c. Reducing fat intake - identifying high fat foods and appropriate lower fat alternatives.
d. Looking at your physical activity - self-monitoring of activity.
e. Increasing your physical activity - identifying activities that can be maintained long term.
f. Changing the environment to promote lower fat and lower calorie intake (stimulus control techniques and shopping skills).
g. Strategies for increasing exercise and reducing the barriers to exercise.
h. Eating at home: food preparation and recipe modification skills to lower fat and calorie intake.
i. Eating away from home: strategies for dieting in restaurants and other social eating situations.
j. Problem solving.

The schedule of visits represents the minimum frequency of contact and can be adjusted for individual participants due to personal constraints such as illness, vacation, and travel. Participants will be taught to record their diet and exercise throughout the initial core curriculum phase of the program. The emphasis of the first 24 weeks will be on achieving the 7% weight loss goal and to achieve at least 150 min/week of moderate intensity exercise, and maintain this level of physical activity throughout the DPP. If participants are having difficulty achieving either or both of these goals, a “tool box” approach will be used to add new strategies to help individual participants achieve the weight and exercise goals. The Lifestyle Resource Core will be responsible for providing an initial list of tool box strategies, and individual case managers can propose additional strategies to the Core for their review. Tool box strategies to increase physical activity would include approaches such as provision of home exercise equipment to the participant or enrolling the participant in a local health club. Tool box strategies for weight loss would include strategies such as increased clinic visits, home visits, and provision of low calorie meals or frozen entrees to the participant.

2. Maintenance Phase.

Following the initial phase, all participants in the intensive lifestyle intervention will receive, at a minimum, monthly contacts for the remainder of the DPP. At least one contact every 2 months must be in person, and must be done by the case manager. The other contact can be by phone. In addition, starting in the second half of year 01 of intensive lifestyle intervention, “short courses” will be offered quarterly for those intensive lifestyle participants who wish to attend. Each course will last 4 - 6 weeks and will focus on a specific topic. Exercise courses will include such topics as resistance training or step aerobics. Nutrition topics might include recipe swap, holiday entertaining or stir fry cooking. Behavioral topics might include “keeping lapses from becoming relapses”, “emotions and eating” or “getting my family involved”. All participants who have completed the 16 session core curriculum will be invited to attend these classes and those participants having problems in specific areas (e.g. not getting enough exercise) will be strongly encouraged to join the appropriate class. The number of participants in these classes and the group make-up will thus differ for each topic. Individual centers will be able to propose ideas for “classes” and some flexibility will be given to centers in terms of which classes to offer (i.e. not all centers will have to offer the same classes), although all centers may need to offer one class related to diet, one to exercise, and one to a behavioral topic each year. Curricula for the 4 - 6 week classes will be developed by the Lifestyle Resource Core, with input from the individual centers. New classes will be introduced each year.

3. Exercise Sessions.

Each center will offer 2 supervised exercise sessions per week. These group exercise sessions may be held at churches, health centers, outdoors, etc. Any type of low intensity activity may be used (such as walking or aerobic dancing). These sessions are provided as an option for participants to increase their exercise. Centers can choose specific staff members to offer these sessions which will be offered

throughout the DPP; however, if sufficient numbers of participants are not utilizing these sessions, this aspect of the protocol may be modified.

7.3.1.1 Changes to the Intensive Lifestyle Program during the Bridge Period

Participants in the intensive lifestyle (ILS) program of the DPP will be seen in person individually by their lifestyle coaches, at a minimum of once every tow months. They will also be offered participation in the HELP program (section 7.3.3) as a refresher or restart. Lifestyle participants will also be called on the alternate months and/or seen more frequently if they are having trouble meeting their weight and activity goals or if desired by the participant. This will ensure that the original treatment plan is maintained as close to plan as possible and will maximize the duration of the lifestyle changes over the longest period possible. Centrally developed motivational programs/campaigns will be used on an as needed basis to encourage maintenance of activity and weight loss. Depending on bridge resources, ILS may be stopped during the bridge period or continued into the follow-up period.

7.3.2 Indices of Adherence

Adherence to the intensive lifestyle intervention is assessed in the following manner:

- Data are recorded on all face to face contacts and phone contacts to ensure that all intensive lifestyle intervention participants receive the 16 core curriculum sessions and monthly contacts throughout the study. Attendance at supervised exercise sessions and group classes is documented (although these are not required of participants).
- Adherence to exercise protocol is determined from self-report questionnaires that assess physical activity, completed at screening step 3, end-months 6 and 12, and then annually, thereafter, in all DPP participants and with intensive lifestyle intervention diaries.
- Adherence to the weight loss goal is determined from the 6 month assessments of body weight.

7.3.3 Healthy Lifestyle Plan - HELP

Overview: Between January and June 2002, all participants (intensive lifestyle (ILS), metformin, placebo, and troglitazone, including those who have converted to diabetes) will be offered a 16-session group implemented lifestyle change program with the same goals used in DPP and similar in content to the core curriculum delivered to the original ILS participants. These will be offered in two eight-session blocks, with the opportunity to enroll for the second set of sessions given at the completion of the first block. The group sessions will also be offered to the former ILS participants as an opportunity for review and restart. The plan will be conducted in a group format, rather than individually. Centers may offer groups at different times of the day and the week, with the goal of completing all sessions by the end of June 2002. Local scheduling decisions may require that some centers extend beyond this date due to the numbers of participants, staffing, etc.

Content: Participants will focus on weight loss first, rather than choosing between weight loss and physical activity as done previously. The sessions will follow this order: 1) Welcome, Getting Started Losing Weight; 2) Be a Fat Detective; 3) Three Ways to Eat Less Fat; 4) Healthy Eating; 5) Move those Muscles; 6) Being Active; 7) Tip the Calorie Balance; 8) Take Charge of What's Around You; 9) Problem Solving; 10) Four Keys to Healthy Eating Out; 11) Talk Back to Negative Thoughts; 12) The Slippery Slope of Lifestyle Change; 13) Jump Start Your Activity Plan; 14) Make Social Cues Work for You; 15) You Can Manage Stress; and 16) Ways to Stay Motivated. Each of these sessions is described in the Lifestyle Manual of Operations. Supervised activity sessions, offered at or by a DPP Center, will be optional. Data will be collected to document attendance at each session, as well as weight and activity minutes throughout the sessions.

All participants who wish to take part in the group lifestyle training will be required to obtain approval from their physician before beginning exercise, with the exception of those who were already participating in an exercise program as part of the ILS. If the physician does not give approval, the participant will not be able to take part in the exercise component of the training.

7.4 Pharmacological Treatment

7.4.1 Description

In addition to the intensive lifestyle intervention, there is one pharmacological intervention and a placebo control group. Metformin 850 mg bid is the pharmacological intervention. A corresponding placebo is used to achieve a randomized, placebo controlled, double masked research design. The following codes are used in this section: MP-metformin placebo and MA-metformin active (850 mg). Metformin is an investigational drug for the treatment of IGT and will be used under an Investigative New Drug (IND) application with the Food and Drug Administration (FDA).

7.4.1.1 Metformin

Metformin is an antihyperglycemic drug of the biguanide class used in the management of non insulin dependent diabetes (NIDDM) in over 90 countries for over 30 years. It was approved for use in the U.S. in 1995 and is distributed by Bristol Myers-Squibb under the trade name Glucophage and manufactured by Lipha, a French pharmaceutical firm.

Metformin reduces the excess hepatic glucose production that characterizes NIDDM without increasing insulin secretion (see section 3.7.1). With reduced hyperglycemia, glucose uptake by muscle and other insulin sensitive tissues is enhanced while insulin levels remain stable or decline. In addition to its antihyperglycemic action, metformin also has antihyperlipidemic effects, particularly the lowering of serum triglyceride levels and is sometimes associated with weight loss.

Metformin is related to phenformin, an agent used in the U.S. in the late 60's and early 70's but withdrawn from use because of the occurrence of severe, often fatal, lactic acidosis. Metformin has been found to cause lactic acidosis rarely (about 0.03 cases per 1,000 person years) and then only when used in persons with renal or hepatic insufficiency or during episodes of hypoxia or circulatory failure.

Before its 1995 release in the U.S., and after review of extensive metformin use in Canada, Europe and other parts of the world, Bristol-Myers Squibb issued an FDA approved package insert providing detailed contraindications, precautions and safety monitoring recommendations for its use in NIDDM. During the DPP all of these recommendations (including periodic assessment of serum creatinine) are strictly adhered to and the maximum dosage used (1.7 gm/day) is less than the maximum recommended (2.55 gm/day).

Metformin is not currently approved for use as a preventive for the development of NIDDM. However, some studies suggest that metformin may be effective in persons with impaired glucose tolerance, similar to those in the DPP, by reducing hepatic glucose release, enhancing insulin sensitivity, or through other mechanisms such as weight loss.

The most common side effects associated with metformin are gastrointestinal. As many as 30% of persons report diarrhea, nausea, metallic taste, abdominal bloating, flatulence or anorexia. These symptoms are generally transient, resolve spontaneously and can be avoided by gradual escalation of dosage. Metformin is not associated with hypoglycemia unless used in conjunction with other glucose lowering medications (sulfonylurea or insulin). About 4% of participants were unable to continue metformin in U.S. clinical trials.

About 6-9% of participants on metformin develop reduced vitamin B12 levels. However, megaloblastic anemia is rare and metformin use has not been reported to cause peripheral neuropathy.

7.4.2 Dosing Schedule

The administration of coded medication, either active or placebo, takes place in three phases: run-in; and post-randomization steps I and II. The use of metformin at the dose of 850 mg twice daily is associated with gastrointestinal side effects at the onset of treatment. These side effects are reduced if the medication is taken with food and the dose titrated from once daily to twice daily over several weeks. Thus, metformin will be administered with food and the dose (MP or MA) will be increased in two steps during the DPP.

7.4.2.1 Pre-randomization

All DPP candidates participate in a three week run-in phase during eligibility screening in which participants take MP (metformin-placebo) twice daily, in the morning and evening with food (see section 6.2.3). Participants will be told that the pills are inactive during the run-in.

7.4.2.2 Post-Randomization - Steps I and II

Volunteers who successfully complete the run-in phase and are otherwise eligible are randomized to one of the three DPP treatments: intensive lifestyle intervention group, placebo control group or metformin group.

Volunteers randomized to one of the two pharmacological treatments then enter Step I of the pharmacological treatment regimen.

Step I

After randomization, pharmacological participants initially take one tablet with the morning meal. This is MP in the placebo control group and MA in the metformin group. This phase will last four weeks.

Step II

After four weeks in Step I (once daily dosage), participants are advanced to Step II (twice daily dosage). This is MP BID in the placebo control group and MA BID in the metformin group.

Table 7-1 Summary of Coded Medication Administration

Step	Week	Time*	Placebo**	Metformin**
Run-In	-3 to 0	AM	MP	MP
		PM	MP	MP
Step - I	0 to 4	AM	MP	MA
		PM	None	None
Step - II	>4	AM	MP	MA
		PM	MP	MA

* AM - Usually before breakfast; may be before first meal of the day
PM - Usually before evening meal; may be taken before bedtime snack.

** M - metformin, A - active, P – placebo

7.4.2.3 Unmasking and open-label follow-up

Participants who were randomized to DPP study medications will be unmasked starting in August, 2001. Those persons without contraindications, who are willing to continue to take it, will be offered Metformin unmasked, in open label format, following amendment of the study IND #49,782 from the FDA. Safety monitoring described below will continue without revision.

7.4.2.4 Metformin Unmasking and Open Label Follow-up During the Bridge Period

Participants originally assigned to metformin will continue to receive it through the bridge period in an unmasked fashion as in protocol version 4.4. This will allow the longest possible period of continued exposure to metformin to determine its longer-term effects. The Steering Committee will design the follow-up study, if funded, during the bridge period. Depending on the bridge resources, metformin may be stopped during the bridge period or continued into the follow-up period.

7.4.3 Restarts and Titration Due to Potential Non-Gastrointestinal Side Effects

If non-gastrointestinal side effects considered likely to be due to coded medications occur and require cessation of coded medications during Step I, all coded medications will be stopped for four weeks. If the non-gastrointestinal symptoms disappear, a second attempt to introduce coded medications is made after four weeks. If symptoms re-occur, the coded medications will again be discontinued. A third try may be attempted after six months and a fourth try after another six months.

Potential non-gastrointestinal side effects include, but are not limited to: headache, mild edema, leg cramps, arthralgia, myalgia, dizziness, mild rashes, and dysmenorrhea. If non-gastrointestinal side effects occur that are considered likely due to coded medications during Step II, the participant will drop back to the Step I dosing schedule for four weeks. A second attempt to restart Step II is made after four weeks. If symptoms re-occur during this second attempt to implement Step II, the participant is restarted at Step I for six months. A third attempt to restart Step II is made after six months. If this third attempt fails, the participant will be maintained at Step I for the remainder of the DPP. Gastrointestinal side effects are handled as indicated below.

7.4.4 Safety Monitoring and Measures to Reduce and Manage Potentially Drug Related Side Effects

A. *Laboratory Safety Monitoring*

During the DPP all participants assigned to one of the two pharmacological treatment groups will have periodic laboratory tests to assess possible toxic effects on the hepatic, hematopoietic and renal systems. These include:

- ALT and AST (liver enzymes) measured at end-months 3 and 6, and then every 6 months.
- A CBC with differential count at end-months 6 and 12, and then yearly.
- A serum creatinine every 6 months during the DPP.

In addition, women of childbearing age will have pregnancy tests when necessary based on symptoms and menstrual history.

B. *Discontinuation of Coded Medication Use During Hospitalizations*

Metformin should not be used in patients with hypoxia or circulatory failure and should be discontinued before the administration of contrast dyes and surgery requiring general anesthesia. To avoid having metformin administered inadvertently to hospitalized DPP participants in whom it may be contraindicated, coded medication will be discontinued during hospitalizations. DPP testing of glucose tolerance will be delayed until coded medication has been resumed for at least two weeks. However, glucose tolerance testing will be performed within 6 weeks even if coded medication has not been restarted, assuming that they do not have a concomitant condition that substantially interferes with glucose tolerance. However, if the participant has a serious condition (e.g. recovering from major surgery, on high doses of steroids, ongoing fibrile illnesses) known to affect the glucose tolerance adversely, the testing will be postponed until the next regularly scheduled testing of glucose tolerance.

C. *Gastrointestinal Symptoms*

These include diarrhea, abdominal pain, vomiting, nausea, a metallic taste, bloating, flatulence and anorexia. They are more likely to occur with metformin but may occur with placebo. If these symptoms are mild and tolerable, coded medications will be continued.

If they are moderate or difficult to tolerate, they will be presumed initially to be due to metformin and the protocol for reductions from Step II to Step I and Step I to temporary cessation will be implemented only for the coded metformin. If, after one week, these gastrointestinal symptoms are not alleviated by reducing or stopping MP or MA, all coded medications will be stopped. Coded medication may be restarted after 4 weeks at Step I. If coded medications cannot be tolerated on this second attempt, a third restart can be attempted at six months. A fourth and final restart can be implemented after another six months.

In the event that diarrhea, abdominal pain or vomiting becomes severe enough to cause dehydration or volume depletion, coded medication will be discontinued immediately and the participant will be evaluated and treated appropriately. Once dehydration or severe pain has resolved, coded medication can be restarted at the previous dose.

D. *Renal Insufficiency*

Serum creatinine safety measurements will be made every six months in all participants in the pharmacological treatment groups of the DPP. Metformin is not known to cause renal insufficiency. However, it is associated with an increased risk for lactic acidosis if used in persons whose glomerular filtration or creatinine clearance rates are below 60 mL/min (per 1.73 m^2 surface area). Thus, metformin use is contraindicated with serum creatinine ≥ 1.5 mg/dL [133 μmol/L] in men and ≥ 1.4 mg/dL [124 μmol/L] in women. If either condition occurs during the study, coded metformin (MP or MA) will be discontinued and serum creatinine rechecked in two weeks. Coded metformin will be restarted if the repeat serum creatinine is < 1.5 mg/dL in men or < 1.4 mg/dL in women. If the serum creatinine is again ≥ 1.5 mg/dL [133 μmol/L] in men or ≥ 1.4 mg/dL [124 μmol/L] in women, regardless of the cause, coded metformin will be stopped permanently and participants will be referred to their health care providers for an evaluation of potential causes of elevated creatinine. Coded metformin will also be discontinued in individuals who have a post-randomization creatinine clearance (based on a 24 hour urine collection) level < 75 mL/min. For participants who are permanently off study medication, elevations in serum creatinine do not require confirmation, but will be reported to the health care provider. A creatinine clearance is only performed post-randomization if the participant turns 80 years old during the DPP and did not receive a creatinine clearance for eligibility.

E. *Anemia*

A CBC will be determined for safety reasons at end-months 6 and 12, and at yearly intervals, thereafter, in all participants in the pharmacological treatment groups. If anemia (defined as a hematocrit < 36.0% in men and < 33.0% in women) or significant macrocytosis develop, or if the hematocrit decreases by 4 or more points from the level at study entry (e.g., from 44% to 40%) the CBC and differential will be repeated within one

month. This evaluation will include measurement of serum B12 levels and exploration of other causes of anemia or macrocytosis, as indicated. Coded medication may be continued if the cause of the anemia is identified and treated. This includes the administration of vitamin B-12 when indicated.

F. *Hepatotoxicity*

Hepatic enzymes (ALT and AST) will be measured at end-months 3 and 6, and then every 6 months, in all participants in the pharmacological treatment groups of the DPP. Hepatic enzyme elevations are rare with metformin and may be no more frequent than with placebo. Nevertheless, metformin should not be used in persons with known active liver disease or hepatic insufficiency.

1. If either ALT or AST level is 1.8 - 3.0 times the upper limit of normal:
   a. Continue coded medication and
   b. Repeat liver function tests at CBL within two weeks.
   c. If repeat ALT or AST remains 1.8 - 3.0 times the upper limit of normal (ULN), continue coded medication at discretion of investigator, with continued monitoring of ALT and AST levels at the CBL monthly until the level is < 1.8 ULN, at which time continue the protocol schedule of liver function testing every 6 months.
   d. If medication is stopped, monthly monitoring is not required; resume the usual schedule of monitoring every 6 months.
   e. If repeat ALT and AST are < 1.8 times ULN, continue coded medication and the protocol schedule of liver function testing every 6 months.
2. If either ALT or AST is > 3.0 times the upper limit of normal:
   a. Stop coded medication immediately and
   b. Repeat liver function tests at CBL within 2 weeks.
   c. If repeat ALT or AST decreases to 1.8 to 3.0 times ULN, re-challenge with the coded medication at discretion of the investigator (since metformin is not associated with liver disease) and continue monitoring as in 1.c., above.
   d. If repeat ALT or AST is > 3.0 times ULN, refer participant to his or her local health care provider for evaluation. LFTs will be repeated in 6 months.
   e. If repeat ALT and AST are < 1.8 times ULN, continue coded medication and the protocol schedule of liver function testing every 6 months.

Participants are instructed that in the event they develop malaise, nausea, vomiting, dark urine, jaundice or right upper quadrant abdominal discomfort, they should stop coded medication and report the symptoms immediately to their DPP clinical center. Upon notification, the clinical center staff must obtain a blood sample as soon as possible (within 1 week) for liver function tests at the CBL. If both ALT and AST are less than 1.8 times the upper limit of normal, the participant may be re-challenged with the coded medication. If either value is > 1.8 times the upper limit of normal then follow testing procedures outlined above.

G. Pregnancy and Nursing

If a woman plans to become pregnant or becomes pregnant, coded medication will be discontinued. Following the pregnancy and nursing, the coded medication will be restarted at the Step I dosage level and then progressed to Step II as outlined above. See the Pregnancy Protocol, Section 7.5.4, for more details about coded medication unmasking and use following pregnancy.

H. Radiological Studies Using Contrast Dyes

Because of the potential danger of contrast induced renal insufficiency and lactic acidosis associated with metformin, under these conditions, the last dose of coded medication will be administered on the day prior to administration of contrast dyes. Serum creatinine level will be checked 48 hours or more after dye administration. Coded medication will be re-started if the serum creatinine levels are in the acceptable range (< 1.5 mg/dL (133 μmol/L) for men and < 1.4 mg/dL (124 μmol/L) for women). A wallet ID will be given to all participants and a warning letter will be sent to all primary care providers to alert them to the fact that participants may be taking metformin and that coded medication needs to be discontinued prior to any radiological studies involving contrast dyes.

I. Lactic Acidosis

Metformin may rarely be associated with the development of lactic acidosis, defined as a metabolic acidosis with lactate ≥ 5.0 mM. If hospitalization or an unexplained metabolic acidosis occurs, coded medication will be discontinued immediately and not restarted. The participant will be treated appropriately.

J. *Hypoxic States - Congestive Heart Failure*

States of hypoxia or hypoperfusion, including acute congestive heart failure and acute myocardial infarction, may lead to lactic acidosis and require discontinuation of coded medication and treatment of the underlying condition. If the underlying hypoxic state is corrected or if CHF is transient (for example, after an acute MI), reinstitution of coded medication may be considered. Medication arm participants who develop CHF (NYHA Functional Class > 2) during the study should have their coded medication stopped. Medication arm participants who develop NYHA Functional Class 2 and require a loop diuretic or digitalis preparation should have their coded medication stopped.

K. *Surgical Procedures*

Because of the risk of metabolic acidosis during general anesthesia and major surgical procedures, coded medication will be suspended prior to such anticipated surgical procedures, with the last dose administered on the day prior to surgery. Coded medication will obviously be held while participants are NPO for procedures. Serum creatinine should be checked after such procedures and coded medication will be restarted if the serum creatinine levels are in the acceptable range (< 1.5 mg/dL (133 µmol/L) for men and < 1.4 mg/dL (124 µmol/L) for women). If an outpatient procedure is scheduled, a letter will be sent to surgeons to alert them to the fact that coded medication must be discontinued prior to surgery.

L. *Dermatological Reactions*

In the event of major dermatological reactions such as generalized urticaria, bullous rashes, exfoliative dermatitis or Stevens Johnson Syndrome, coded medication will be discontinued immediately and not restarted. For localized skin reactions, coded medication may be discontinued if the skin reactions are potentially drug related. If the rashes clear, coded medication may be restarted after four weeks, starting at the Step I dosage level and then progressing to Step II after another four weeks. If localized skin reactions recur with restarting the coded medication, coded medication should be discontinued.

M. *Headaches*

Metformin has sometimes been associated with transient headaches, although not more frequently than placebo. However, headache is not a reason to decrease or discontinue the coded medication unless severe and no other causes are found.

7.4.4.1.A Laboratory Safety Monitoring During the Bridge Period

Participants assigned to metformin who continue to take it will have safety monitoring laboratory tests at annual and semi-annual visits per Section 7.1 and Table 12.2. Participants who are not taking metformin will not have safety laboratory tests drawn. If there are medical reasons why metformin was stopped, appropriate safety testing will be continued as clinically indicated. Participants who were assigned placebo will not have safety laboratory tests drawn.

7.4.5 Indices of Adherence

The goal of the pharmacological treatment is to optimize adherence to the pharmacological regimen, while maximizing retention of participants in the DPP. Assessment of adherence to prescribed coded medication will provide clinic staff a means to identify participants having problems with adherence.

Adherence to the pharmacological treatment will be assessed by the following:

- By visual inspection of participant's returned pill containers, the case manager will estimate percent of prescribed coded medication taken.
- By conducting a brief, structured interview, the case manager will assist participants to (a) identify problems in adherence to the pharmacological treatment and (b) estimate adherence to the prescribed protocol since their previous visit.

Results of the case managers' adherence estimate and the interview with participant will guide the consideration of strategies to improve adherence.

7.4.5.1 Indices of Adherence for the Bridge Period

Participants continuing to take metformin will have the same measures of adherence collected as specified in protocol version 4.4, however, they will be collected at six monthly intervals. Participants who were previously assigned placebo will not have adherence measures collected.

7.5 Definition and Management of Concomitant Conditions

Clinical centers are neither sufficiently staffed nor funded to provide all primary care or ancillary care to participants involved in DPP. Whenever possible and acceptable to the referring primary care provider, conditions significantly affecting either the primary or secondary outcomes of the DPP should be cared for as specified by protocol within the context of the DPP in order to protect the integrity of the DPP research questions. In addition, treatments for concomitant conditions which could potentially affect either the primary or secondary outcomes should be avoided. The following sections provide guidelines for therapy which should be vigorously pursued. However, investigators and staff must be sensitive and at times flexible with regard to the prerogative and needs of the primary care providers who have a participant enrolled in DPP. If the following conditions arise during the course of DPP, the investigator will contact the primary care provider by letter, informing him/her of the condition and provide a copy of the treatment options developed for DPP participants. Discussions with the primary care provider should be conducted in the spirit of negotiation and collegiality, with the intent of including the referring primary care provider in DPP operations. Treatment of all other conditions not directly related to DPP outcomes will be treated by referring primary care providers, community resources, or the clinical center as determined by referral patterns. Participants with concomitant conditions who otherwise meet eligibility criteria will remain on their pre-enrollment therapy.

7.5.1 Hypertension

There is a strong association between NIDDM and hypertension, apparently independent of age and obesity (Modan, et al., 1985; Yudkin, 1991). Based on available cross-sectional data, a cohort of participants in the likely age range for the DPP will include a high proportion (~35%) who will have hypertension. Following are recommendations for treatment:

A. Goals of Therapy:

1. Therapy should aim at maintaining BP <140/90 mm Hg. However, in participants over age 60, the initial goal of therapy should be to lower SBP to <160 mm Hg for those participants with SBP >180 mm Hg and to lower blood pressure by 20 mm Hg for those with SBP between 160 and 179 mm Hg. If this is well-tolerated, blood pressure should be lowered to target levels noted above.
2. At SBP levels of 140 to 160 mm Hg in participants with isolated systolic hypertension, life-style modifications may be sufficient to lower blood pressure. Antihypertensive drug therapy should be carried out more cautiously in older participants. Blood pressure measurements may be required in the standing as well as seated positions, and antihypertensive treatment should be initiated with smaller doses than usual.

B. Therapy:

1. Diet: Non-pharmacologic therapy should be employed initially, consisting of a prudent diet aimed at weight reduction if necessary and moderate sodium restriction (< 2.3 g of sodium), limitation of alcohol intake, and encouragement of physical activity. The participant and primary care provider will receive a standard printed set of instructions outlining the goals. Participants will be advised to lose weight if overweight, limit alcohol intake to < 1 oz per day of ethanol, to exercise aerobically regularly, to maintain adequate potassium, calcium and magnesium intake, to stop smoking, and to reduce dietary saturated fat and cholesterol intake. These guidelines will reinforce the standard lifestyle recommendations already in place for DPP. If the blood pressure does not fall into an acceptable range after 4-6 weeks, then pharmacologic therapy should be recommended.
2. Drug therapy should be based on specific needs of the participants, potential side effects of therapy, and consideration of other factors such as cost and availability. The following classes of agents can be employed as first-line agents; substitution and/or addition of drugs should be considered if blood pressure targets are not attained.
    a. Angiotensin converting enzyme inhibitors.
    b. Calcium channel antagonists.
    c. Alpha adrenergic blockers.
    d. Centrally-acting $alpha_2$-agonists.
3. Because of their potential to worsen glucose intolerance, diuretic agents should be strongly discouraged for the treatment of hypertension in DPP participants (Bengtsson, et al., 1984; Skarfors, et al., 1989; MRC Working Party, 1981; Saxman, et al., 1994). When indicated for the treatment of edematous states or congestive heart failure, diuretics may be used with caution, and will be noted in the subsequent data analysis as a confounding variable. This applies to all diuretic therapy including

thiazide and loop diuretics; there should be careful monitoring of serum potassium to maintain values above 4.0 mEq/L.

4. Beta-blockers should be strongly discouraged for the treatment of hypertension in DPP participants. When indicated for the treatment of CAD or tachyarrhythmias, beta-blockers should be used with caution and will be noted in the subsequent data analysis as a confounding variable. This applies to labetalol (combined alpha-beta blocker) as well as both selective and nonselective beta-blockers.

7.5.2 Lipids

7.5.2.1 Dietary Treatment

Although the available data suggests that IGT is associated with an increased risk of atherosclerosis, IGT is not currently designated by the National Cholesterol Education Program (NCEP) report (National Cholesterol Education Program, 1993) as one of the cardiovascular risk factors; therefore, for the purposes of diagnosis and treatment of dyslipidemia in DPP, IGT will not be considered a cardiovascular risk factor.

The recommended initial dietary treatment for dyslipidemia (NCEP Step I) coincides exactly with the American Diabetes Association dietary recommendations for management of diabetes regarding the intake of saturated fat and cholesterol and the approach to excess body weight management (American Diabetes Association Consensus Statement, 1993). Thus the standard lifestyle recommendations for all DPP participants, independent of their lipid status, provides adequate initial dietary treatment for those with hyperlipidemia. Dietary treatment in DPP will be introduced at the time the DPP treatment is initiated and will be maintained by clinical centers. Primary care providers will be informed of these efforts. All participants will receive the same instruction by means of customized handouts that will contain dietary information based on the American Diabetes Association/NCEP Step I dietary recommendations. This will meet acceptable standards of care for both IGT and dyslipidemia while achieving a standardized level of dietary instruction.

Therefore in dyslipidemic participants:

A. At 3 months, dietary recommendations will again be provided for dyslipidemic participants who were identified at baseline. A lipid profile will not be repeated at 3 months to assess dietary treatment, except in those participants with CVD.

B. A lipid profile will be repeated at 6 months to identify participants who may qualify for lipid lowering drug therapy.

7.5.2.2 Drug Treatment

The cutpoints for initiating drug therapy for hypercholesterolemia have been recommended by the NCEP to be at an LDL-cholesterol of >220 mg/dL for premenopausal women (or men <35 years of age), at >190 mg/dL for low-risk diet-resistant participants, at >160 mg/dL for high risk participants and at >130 mg/dL (or less depending on the primary care provider’s judgment) for participants with CVD. Participants with borderline-high (200-400 mg/dL) or high (400-1000 mg/dL) triglyceride levels are not routinely recommended for drug therapy unless there is concomitant CVD, a family history of premature CVD, concomitant hypercholesterolemia and low HDL-cholesterol, familial dysbetalipoproteinemia or familial combined hyperlipidemia, or a history of pancreatitis. Pharmacotherapy should be prescribed for participants with triglyceride levels in the region of 1000 mg/dL or greater. Lastly the NCEP recommends reducing LDL-cholesterol to <130 mg/dL in individuals with low HDL-cholesterol (35 mg/dL) and one other CVD risk factor. DPP participants will follow NCEP recommendations for drug treatment of dyslipidemia.

Dyslipidemia will be diagnosed according to the proposals discussed above and are summarized in the table below. IGT will not be considered to be a cardiovascular risk factor. As a secondary outcome, specific lipid levels measured during DPP will be masked (see section 5.6.3). However, hyperlipidemia participants will be unmasked to lipid results at 6 months if they qualify for drug therapy (at 3 months for CVD participants). Cardiovascular risk factor status will be updated whenever a lipid profile is repeated. Conversion to diabetes will add one risk factor.

Table 7-2

	Diagnosis	Diet therapy Cutpoint and Target for Therapy	Drug therapy Cutpoint
LDL-cholesterol with <2 risk factors	160 mg/dL (4.15 mmol/L)	160 mg/dL (4.15 mmol/L)	190 mg/dL (4.90 mmol/L)
LDL-cholesterol with ≥2 risk factors	130 mg/dL (3.35 mmol/L)	130 mg/dL (3.35 mmol/L)	160 mg/dL (4.15 mmol/L)
LDL-cholesterol with CVD	100 mg/dL (2.60 mmol/L)	100 mg/dL (2.60 mmol/L)	130 mg/dL (3.35 mmol/L)
Triglyceride	400 mg/dL (4.52 mmol/L)	200 mg/dL (2.26 mmol/L)	(400 mg/dL (4.52 mmol/L))*

* based on the primary care provider's judgment and according to NCEP guidelines

7.5.2.3 Treatment Protocols

A. *Diet treatment*

All participants independent of lipid status will be instructed in the equivalent of an NCEP Step I diet on entering DPP. The diet recommendations will be provided by the centers and primary care providers will be informed of this. Participants in the standard lifestyle recommendation groups will receive instruction by means of customized handouts and those in the intensive lifestyle intervention group will receive specific instructions according to the dietary design that has been developed for this group. Participants will receive repeat dietary instruction using the same methods described above.

B. *Pharmacotherapy*

Participants whose lipid levels qualify them for drug therapy at 6 months will have reached a secondary outcome and will be unmasked to lipid levels so that a decision can be made regarding hypolipidemic drug selection. Primary care providers will be informed. Thereafter actual lipid levels will be reported on these participants. Participants with CVD will have a 3-month lipid profile performed; if their levels qualify them for pharmacotherapy they will be unmasked to lipid levels for drug selection and early initiation of drug treatment.

C. Drug Treatment Options

1. Pure hypercholesterolemia:
   - HMG-CoA Reductase Inhibitor
   - Sequestrants
2. Pure hypertriglyceridemia:
   - Gemfibrozil (no alternative. Addition of sequestrant may be necessary if LDL cholesterol rises)
3. Combined hyperlipidemia:
   - Gemfibrozil plus a sequestrant
   - HMG-CoA Reductase Inhibitor
   - HMG-CoA Reductase Inhibitor plus gemfibrozil (in selected cases)

7.5.3 Psychological Diseases and Use of Psychoactive Agents

Severe psychiatric disorders can interfere with the compliance of participants with research protocols, and some psychoactive drugs are known to alter glucose tolerance and/or induce weight changes. Due to the high prevalence of psychiatric disorders in the general population, it is very likely that some participants will develop them after enrollment in the DPP.

Several psychoactive agents have been reported to interfere with appetite and/or glucose tolerance (Gray, et al., 1992; Levine, et al., 1989). The different selective serotonin reuptake inhibitors are considered to be a single class. The use of fluoxetine up to 20 mg/day (or equivalent doses of other SSRI) is permissible. The use of fluoxetine (or equivalent doses of other SSRI) at doses higher that 20 mg/day is an exclusionary criteria for the DPP and use subsequent to randomization will be noted in the data analysis as a confounding variable.

7.5.4 Pregnancy

Women with a history of gestational diabetes (GDM) are one of the potential groups of interest for the DPP. These women, together with many other participants in DPP, may be of childbearing potential during the course of the DPP. Data from available cohorts suggest that about 6 percent of women of childbearing age may be expected to get pregnant in a given year.

7.5.4.1 Indications During Pregnancy

Metformin is contra-indicated in pregnancy although data on adverse effects on the fetus or the mother are scant. The embryotoxic effects of biguanides have recently been evaluated in the *in vitro* cultured mouse embryo model (Denno, et al., 1994). Because of the lack of teratogenicity of metformin in the few available studies, this drug is classified by the FDA as pregnancy category B (no evidence of risk in humans, animal findings negative).

There is no general contraindication for women continuing the exercise intervention during pregnancy (American Academy of Pediatrics, American College of Obstetricians and Gynecologists, 1988). The 150 min/wk target for women in the intensive lifestyle intervention should not require monitoring, but because of the lack of data on ketosis with vigorous exercising, regimens that exceed 1000 kcal per week may require monitoring of ketosis. Dieting for weight loss during pregnancy can be dangerous therefore guidelines for calorie intake (American Academy of Pediatrics, American College of Obstetricians and Gynecologists, 1988) will be recommended.

Women of childbearing age who are fertile, meet other eligibility requirements and wish to participate in the DPP will be informed of the potential risks to a pregnancy conceived while on any DPP pharmacological treatment. Women who consent to participate will be asked to practice reliable birth control including systemic hormones, intrauterine devices and barrier methods (diaphragm, male or female condom, cervical cap) with concomitant intravaginal spermicide.

7.5.4.2 Safety Monitoring

Women in the intensive lifestyle intervention group will be asked to obtain pregnancy tests if pregnancy is suspected. Women in the pharmacological treatment group will be asked to complete monthly menstrual diaries and to get immediate pregnancy testing if their menstrual cycles are more than one week overdue or they otherwise suspect they are pregnant.

7.4.5.2.A Pregnancy Monitoring During the Bridge Period

Women continuing to take metformin will continue pregnancy monitoring as in protocol version 4.4. Women in the former ILS and placebo groups will no longer monitor or be tested for suspected pregnancy.

7.5.4.3 DPP Interventions During Pregnancy

Women randomized to the pharmacological treatment who are found to be pregnant while taking coded medication will have their coded medication discontinued and be immediately unmasked to the pharmacological treatment assignment. Information on the potential teratogenicity of metformin will be provided to both the DPP participant as well as her provider(s) of obstetrical care in order to facilitate an informed decision on further handling of the pregnancy.

Recommendations that exercise should continue with a target of 150 min/wk per week will be forwarded to the providers of obstetrical care for DPP participants in the intensive lifestyle intervention who get pregnant during the course of the DPP. These principles will also apply to those women randomized to standard lifestyle recommendations.

Pregnancy will require modifying the diet intervention to accommodate the increase in calories recommended for a healthy pregnancy, and for lactation (American Academy of Pediatrics, American College of Obstetricians and Gynecologists, 1988). The recommended average daily caloric intake for pregnant women is 30-35 kcal/kg IBW. These recommendations should be forwarded to the provider(s) of obstetrical care for DPP participants who get pregnant during the course of the DPP, regardless of the DPP treatment group. DPP monitoring and visit schedules will be determined by the date of randomization.

7.5.4.4 DPP Interventions During Breast Feeding

The intensive lifestyle intervention, with caloric requirements adjusted to account for breast feeding, can be introduced as soon after delivery as is feasible; we recommend within the first month. Women who choose not to breast feed should return to their pre-pregnancy lifestyle intervention, including dietary targets, within the first month following delivery. For women who choose not to breast feed, weight targets should return to pre-pregnancy levels regardless of extra weight that may have been gained during pregnancy. For women who breast feed, weight targets should be suspended until lactation is finished and then should be re-established at pre-pregnancy levels. Coded medication will be suspended for the duration of breast feeding.

7.5.4.5 Outcome Assessment Following Pregnancy

DPP participants who become pregnant during the DPP are likely to develop gestational diabetes, and many of these women will require insulin. The standard of care for follow-up after gestational diabetes is to assess glucose tolerance at six to eight weeks post-partum. Women who become pregnant during the DPP will have outcome assessment suspended until 6-8 weeks following delivery. This outcome measure following pregnancy will always be an OGTT. They will then attend the next regularly scheduled outcome assessment visit based on their original DPP follow-up schedule. Women meeting ADA criteria for diabetes will have reached the DPP primary outcome.

For those DPP participants who require insulin during pregnancy, assessing the ongoing need for insulin should begin in the hospital immediately post-partum. Women discharged on insulin should be evaluated with home glucose monitoring, followed by their providers of obstetrical care, to determine the ongoing need for insulin. Based upon post-partum monitoring, some women may remain on insulin or be started on oral hypoglycemic agents by their obstetrical/primary care provider(s). Participants treated with insulin or oral agents will not be re-started on their coded medication, unless and until their need for therapy resolves.

There may be some women who are still being treated by their primary care provider(s) with insulin or oral agents at the time of their first outcome assessment following pregnancy (i.e., 6-8 weeks following delivery). To insure standardized assessment of outcomes, therapy must be stopped for the OGTT. If cessation of therapy is not possible, two elevated fasting blood glucose determinations may be used to define an outcome of diabetes in place of the OGTT.

7.5.5 Smoking

The prevalence of smoking among people with impaired glucose tolerance is estimated to have declined in the past decade to about 20-25%, compared to the NHANES II data of 30-35%, and is consistent with U.S. smoking reduction overall (Harris, 1989). Thus, 800-1000 DPP participants are expected to be current smokers. DPP must follow the established public health policy to reduce the prevalence of smoking by discussing smoking as a compounding risk factor for CVD and emphasizing the overall benefits to health for those who quit.

Recommendations from the National Cancer Institute for practicing physicians include asking about smoking, advising smokers to quit, and assisting those who want to stop smoking by providing follow-up (Glynn, et al., 1989). These strategies will be employed by DPP personnel at the annual visits for participants, with distribution of educational materials to those expressing an interest in smoking cessation. Simultaneous referral back to their primary care providers and planned programs can also be offered.

7.5.6 NIDDM

7.5.6.1 Diabetes Discovered At Screening

Given the high risk characteristics of the volunteers undergoing screening for participation in DPP, it is anticipated that approximately 4000 individuals will be found to have previously unrecognized diabetes. Those individuals who have diabetes mellitus by ADA criteria will receive an informational letter indicating that they may have diabetes mellitus and should be seen by their primary care provider or available community resources for evaluation to confirm the diagnosis of diabetes and to initiate appropriate therapy. Participants will be given copies of their laboratory studies for their primary care providers, but DPP personnel will not participate in any further evaluation or treatment of these participants. Volunteers who do not have a primary care provider will be referred to local health care facilities.

7.5.6.2 Interim Visits For Symptoms

Following enrollment and randomization in the DPP, participants will be seen on a quarterly basis for assessment of adverse events. If a participant develops symptoms consistent with uncontrolled hyperglycemia, he or she will be instructed to come to the clinical center for assessment of an adverse event and undergo a fasting blood glucose determination. This test may be performed locally if needed for safety reasons, however a sample must be sent to the CBL for outcome assessment. If the centrally read fasting glucose is ≥ 126 mg/dL, a repeat test will be performed within 6 weeks to confirm the diagnosis. The participant will have reached the DPP primary outcome if fasting glucose ≥ 126 mg/dL persists.

7.5.6.3 Intervention and Follow-up for Participants with Diabetes and Fasting Glucose <140 mg/dL

Assuming a 7.5% annual conversion rate in the control group and a 33% reduction in hazard rate in the intervention groups, 571 participants are anticipated to meet the DPP primary outcome during the course of the DPP. Participants, investigators, and primary care providers will be unmasked to the diagnosis of diabetes. In addition, all subsequent glucose and hemoglobin A_{1c} determinations will be unmasked to participants and investigators. The pharmacological treatment group assignment will remain masked.

After informing the participant and the primary care provider of the diagnosis and explaining the significance, the investigator in conjunction with the primary care provider will endeavor to maintain the participant on their assigned treatment. Further DPP intervention will include intensification of any lifestyle intervention to which the participant has been randomized. For participants assigned to the intensive lifestyle intervention, intensification of diet and exercise with the aim of greater adherence and improved response will be implemented. For participants randomized to pharmacological treatment, the standard lifestyle recommendations (which are consistent with ADA Guidelines) will be reinforced as soon after the diagnosis as possible and the participant will be seen one month and three months later (ADA Clinic Practice Recommendations, 1998). Subsequent reinforcement of standard lifestyle recommendation will occur at the scheduled three month interval visits. In addition, all participants developing diabetes will be offered self monitoring of blood glucose (SMBG) with the option of monitoring fasting glucose levels two to three times weekly as well as during any acute illness or in the event of symptoms such as polydipsia, polyuria or polyphagia. SMBG results may be reviewed at quarterly visits and the participants will be instructed to inform the clinical sites if fasting glucose determinations exceed 140 mg/dL.

DPP participants will continue to be seen at quarterly intervals for clinical assessment. Secondary outcome measurements will continue to be performed. In addition, fasting glucose and hemoglobin A_{1c} determinations will be obtained at quarterly visits.

7.5.6.3.A Intervention and Follow-up for the Participants with Diabetes and Fasting Glucose < 140mg/dL

Participants who converted to diabetes <u>prior to</u> the start of the bridge period will be invited to attend the HELP program. They will continue to receive education, supplies, visits, and other protocol defined activities as in protocol version 4.4. If participants developed diabetes <u>during</u> the bridge period, and they were not already attending the HELP sessions, they may be invited to attend, if the timing and content are deemed appropriate by clinic staff. If they were attending the HELP sessions, staff may combine required diabetes visits with HELP visits, if possible and convenient for staff to do so.

Participants who develop diabetes while on open-label metformin will be asked to maintain their medication as in protocol version 4.4 as long as their fasting blood glucose remains < 140 mg/dL.

7.5.6.4 Intervention and Follow-up for Participants with Diabetes and Fasting Glucose ≥ 140 mg/dL

In the event that participants progress to fasting glucose in excess of 140 mg/dL on two occasions, coded medication will be discontinued. Participants, investigators, and primary care providers will remain masked to the pharmacological treatment assignment until the end of the DPP. A stepped care protocol for treatment of diabetes mellitus as recommended by the American Diabetes Association will be recommended to the primary care provider. The protocol will include increased SMBG frequency and therapy with pharmacologic agents with the goal of achieving pre-prandial capillary glucose measurement 80 - 120 mg/dL (bedtime values of 100 - 140 mg/dL)and hemoglobin A_{1c} determinations < 7%. Additionally, participants will be followed by their primary care providers for the development of any diabetes-related complications such as retinopathy or neuropathy. All DPP-related glucose tolerance testing for insulin secretion or insulin sensitivity studies will be terminated at this time and replaced with unmasked fasting plasma glucose and hemoglobin A_{1c} values. However, participants will continue to be followed at scheduled intervals to collect other outcome data including measurements of cardiovascular outcomes.

7.5.6.4.A Intervention and Follow-up for Participants with Diabetes and Fasting Glucose ≥ 140 mg/dL

Participants who converted <u>prior to</u> the start of the bridge period will be invited to attend the HELP program. They will continue to receive education, supplies, visits, and other protocol defined activities as in protocol version 4.4. If participants developed diabetes <u>during</u> the bridge period, and they were not already attending the HELP sessions, they may be invited to attend, if the timing and content are deemed appropriate by

clinic staff. If they were attending the HELP sessions, staff may combine required diabetes visits with HELP visits, if possible and convenient for staff to do so.

Participants who develop diabetes prior to or during the bridge period will be referred for care and treatment as determined by their primary care physician.

7.5.7 Cardiovascular Disease

Cardiovascular events have been chosen to be one of the outcomes in DPP. The incidence of CVD is increased in participants with newly diagnosed NIDDM, and the risk of deaths in non-diabetic participants with impaired glucose tolerance is two times higher than in participants with normal glucose metabolism. The upper range of postload glucose distribution in a non-diabetic population is associated with a two- to three-fold increased mortality in middle-aged men and women (Stamler, et al. 1979; Fuller, et al. 1980; Eschwege, et al., 1985). On the other hand, many of the cardiovascular diseases themselves, or their treatments, have an effect on proposed treatments or outcomes of the DPP, or vice versa.

Cardiovascular diseases (CVD) in participants recruited to the DPP are significant for three reasons:

- CVD may have an effect on prognosis of the participants recruited,
- The symptoms of CVD may have an effect on the capability of participants to follow the guidelines of treatment especially in the intensive lifestyle intervention group, and
- The conversion rate from IGT to NIDDM may be higher in participants with CVD than in participants without CVD.

Recommendations

A. Exercise Testing

1. Participants randomized to the intensive lifestyle intervention group must have had a symptom-limited maximal exercise tolerance test within 6 months prior to initiation of any exercise program, or will have a symptom-limited maximal exercise tolerance test prior to initiation of any exercise program, if they have:
    - previous hospital verified myocardial infarction; or
    - self reported history of “heart attack”; or
    - abnormal Q-waves in their ECG > 0.03 seconds in duration.
2. Sub-maximal exercise tolerance testing with simultaneous ECG and blood pressure monitoring will be performed for men older than 40 years and post-menopausal women not on hormone replacement therapy who have at least two of the following risk factors:
    - hypercholesterolemia, S-Chol > 240 mg/dL
    - hypertension, systolic BP > 160 and/or diastolic BP > 90 on two separate measurements or on medication for hypertension
    - current smoking
    - family history of CVD at the age less than 55 years

No exclusions will be made based on exercise test results, but participants who are classified by the results to have high risk of cardiovascular complications during the exercise should not be allowed to participate in the exercise program until receiving definitive therapy. High risk groups based on the results of exercise tests are those who have:

- angina pectoris at the low exercise level (< 6 METs); or
- ischemic ST segment depression > 2 mm at any level of exercise; or
- decline in systolic blood pressure > 15 mm Hg; or
- ventricular arrhythmia induced by exercise.

All other participants who have symptoms or signs of CVD during the exercise test are eligible to participate in the exercise program, but the level of the program must be individually adjusted. These participants must be taught to measure their heart rate during exercise and to keep exercise at a safe level (i.e. no symptoms of angina pectoris or dyspnea on exertion and maintenance of heart rate at least 10 BPM lower than that level at which symptoms appeared during the exercise tolerance test) (Fletcher, et al., 1990).

3. CVD management and exercise testing for the bridge period
   All participants who wish to take part in the group lifestyle training will be required to obtain approval from their physician before beginning exercise, with the exception of those who were already participating in an exercise program as part of the ILS. If the physician does not give approval, the participant will not be able to take part in the exercise component of the training.

B. Myocardial infarction or unstable angina during the DPP

Participants who have myocardial infarction or unstable angina during the DPP should be treated according to the community standards. All participants in the pharmacological treatment groups will be eligible to continue to follow the DPP protocol after myocardial infarction without any major interruptions, unless specific contraindications arise. According to the American Heart Association Guidelines a submaximal exercise test should be performed within three weeks after an acute MI (Fletcher, et al., 1990). A maximal exercise test should be done more than 3 weeks after myocardial infarction, when the participant is ready to resume full activities. The participants in the intensive lifestyle intervention group should discontinue the exercise program after myocardial infarction until the risk stratification by exercise testing is done 6-8 weeks post-MI. The decision whether these participants are allowed to continue the exercise program or whether their exercise program should be modified is based on the exercise tolerance test.

C. New angina pectoris

Participants who have new symptoms suggesting angina pectoris during the DPP should be treated by their primary care providers according to the community standards and cardiological evaluation and exercise tolerance testing may be recommended to them. Participants in the intensive lifestyle intervention group will have their exercise program discontinued until the cardiological evaluation has been performed and their eligibility to continue the exercise protocol should be reconsidered after the results of the evaluation are available. Participants assigned to the pharmacological treatment can continue the DPP protocol without any major interruptions.

D. PTCA or coronary by-pass (CABG)

According to the American Heart Association Guidelines, exercise tolerance testing should be performed in the routine follow-up of participants who have undergone PTCA, and to every participant who complains of chest pain during exercise after these procedures. The participants of DPP who undergo PTCA or CABG during the DPP should be allowed to discontinue the exercise component of the DPP protocol for up to six months, if necessary. After this period their health status should be re-evaluated to determine their continuation in the exercise protocol with results of exercise tolerance testing playing a pivotal role. If these participants are in the intensive lifestyle intervention group, they will follow a modified exercise protocol for the rest of the DPP, if necessary.

E. Medical treatment of CVD

Beneficial effects of beta-blockers and ACE inhibitors on mortality and recurrent CVD events after myocardial infarctions are so clear that we cannot restrict their use after myocardial infarctions. The participants with heart failure should be treated according to the community standards and no restrictions on the use of any drugs will be made.

7.6 Retention Monitoring and Recovery of Inactive Participants

Key to both the power and generalizability of findings of DPP is the retention of a large portion of participants throughout the entire DPP; however, recruitment, randomization, and retention of participants for DPP is likely to be difficult. Some of this is due to the fact that participants, although they will have IGT, are asymptomatic. Furthermore, IGT is not presently designated as a disease. In addition, participants who are interested in preventing diabetes will have no choice about the treatment group to which they are assigned. It is anticipated that persons who contemplate participation because of an interest in lifestyle modification may be quite different from those who are willing to take medications. Additional difficulty is imposed by the fact that some test results are masked. However, the greatest problem will probably be due to the demands placed upon participants in DPP with respect to time commitment, transportation, parking, and child or elder care, which vary considerably among the target populations. Steps to maximize retention of participants to the DPP are based upon these considerations.

Since a decision to discontinue follow-up visits is a clear loss to DPP, such decisions are considered not irrevocable and recovery efforts are required. Thus, enrolled participants who make a decision to discontinue follow-up are told they are welcome to return to DPP should they change their minds. Reasons for deciding to discontinue follow-up are explored with the participant. Participants continue to be contacted on a progressively less frequent schedule, starting monthly and decreasing to semi-annually to remind them of the opportunity to re-enter DPP and to maintain contact with them for possible recruitment for the final assessment at the end of DPP.

7.6.A Retention Monitoring and Recovery of Inactive Participants for the Bridge Period

Participants who do not wish to participate in the HELP program will be encouraged to continue to participate in data collection visits until the bridge period is completed. Other aspects of retention monitoring will be maintained as resources allow.

7.6.1 Steps to Maximize Adherence and Retention

Adherence and retention in DPP is fostered by: a comprehensive array of participant education procedures which requires the interest, responsiveness, and continuous availability of professional staff; motivational programs, group activities, and rewards deployed according to the judgment of each participating center; plus removal of barriers to participation. Central training for the Program and Recruitment Coordinators in the techniques of "motivational interviewing" assists them in providing a framework based on this concept at each center. In addition to providing education regarding details of the interventions, much of staff contact with participants is of a general, supportive nature, addressing frustrations with DPP, problems in maintaining adherence, and even general life issues participants may face.

7.6.1.1 Social Support

The underlying philosophy of retention of participants is to encourage Program and Recruitment Coordinators, case managers, receptionists, and all other staff to interact with participants in a manner which enhances their sense of bonding to DPP. Additional tactics such as newsletters, outings, birthday and anniversary cards, and the like may be construed as facilitators of a sense of social connection with DPP, its staff, and fellow participants. It is likely that these connections and not the more tangible rewards themselves are the more important incentives to retention.

Because the kinds of activities and rewards that promote the social connections that bond participants to DPP will take very different shapes in different communities and groups, a high level of local development and design of these is desirable. This is facilitated by a menu of nationally available materials and strategies and frequent contact among centers to exchange suggestions such as:

- Quarterly newsletters, developed nationally but able to be tailored locally, including items of interest about participants and staff in different DPP sites to encourage a sense of community within DPP;
- Individual incentives that are less expensive when purchased in bulk and are ordered by Centers as desired from the Coordinating Center, such as mugs, T-shirts, and caps designed to raise a sense of identity with, and pride, in DPP;
- Locally purchased items such as movie tickets, bowling passes, sports tickets, anniversary cards, exercise video and audio tapes, and gift certificates to create a sense of identity with the local center;
- Refreshments (coffee, tea, sodas) for participants at clinic visits;
- Group social gatherings such as holiday parties, picnics, or sporting or cultural events;
- Additional inexpensive tokens such as key chains, pens, pencils, etc., that may be purchased in bulk if desired by several Centers.

7.6.1.2 Removal of Barriers to Participation

In addition to these incentives, resources for removing barriers to participation such as child or elder care, transportation, and parking expenses are important considerations. These resources are considered separate from the incentives, as necessary support, to enable participants to adhere to treatment regimens (compliance) and to attend outcomes data collection visits (retention). These resources, given as cash, transportation vouchers, or parking passes, may vary greatly among the Centers.

7.6.1.3 Honoraria

In addition, an honorarium will be paid to participants in recognition of the time and effort spent in the DPP. All participants receive this payment twice a year if participants have successfully completed scheduled visits and procedures.

7.6.2 Monitoring Retention

Recovery of patients on inactive follow-up uses protocols that are standardized according to each of the DPP treatment groups' attendance and adherence demands. These protocols include criteria for identifying participants whose level of adherence and/or attendance should trigger recovery efforts, as well as a graded hierarchy of recovery efforts. This hierarchy of efforts ranges from brief telephone discussion of missed meetings

to an individualized counseling session with the case manager or other appropriate staff member in which the participant can review reasons for staying in DPP, concerns regarding DPP, and decisions about remaining a participant.

7.6.2.1 Visit Monitoring

There is a computer-based monitoring system to record each visit with participants. One purpose of this system is to identify participants who are having problems with adherence to the protocol. Another purpose of the system is to identify participants who may be candidates for discontinuing follow-up of DPP and thus qualify for recovery efforts.

7.6.2.2 Measures of Stress and Social Support

The following self-administered questionnaires will be completed during the DPP (see Section 12 for frequency of administration) for purposes of predicting adherence and retention, and determining the positive or negative impact of DPP treatments.

- **Retention and Treatment Monitoring Measures (Form Q06):**
- - Life events index
- - Social provisions scale
- - Family household assessment
- **DPP-Specific Support Measures (Forms Q09 and Q10):**
- - Baseline Visit (Form Q09)
- - Follow-up Visits (Form Q10)

8. ADVERSE EVENT REPORTING *8-1*
8.1 Definitions 8-1
8.2 Eliciting and Recording Adverse Events 8-1
8.2.1 Expedited Reports 8-1

8. ADVERSE EVENT REPORTING

8.1 Definitions

An adverse event is defined as any medical problem experienced by a DPP participant that is not a benefit to the participant whether or not considered intervention-related by the clinical center staff. Serious adverse events have been defined to include any adverse experience occurring at any dose that results in any of the following outcomes:

- Death
- A life-threatening adverse experience
- Inpatient hospitalization or prolongation of existing hospitalization
- A persistent or significant disability/incapacity; or
- A congenital anomaly/birth defect
- Important medical events that may not result in death, be life-threatening or require hospitalization if, based on appropriate medical judgment, they may jeopardize the participant and may require medical or surgical intervention to prevent a serious adverse event.

Non-serious adverse events are all adverse events which do not meet the above criteria for "serious".

A treatment-emergent adverse event is defined as any event not present prior to the initiation of the treatments or any event already present that worsens in either intensity or frequency following exposure to the treatments. A baseline-emergent adverse event is defined as any event which occurs or worsens during the staged screening process (after informed consent) including the randomization visit.

8.2 Eliciting and Recording Adverse Events

Reporting of adverse events will be accomplished by collecting information on adverse experiences during the staged screening process and at Standard (Form F01), Major (Form F02) and Interim (Form F03) follow-up visits. In order to avoid bias in eliciting adverse events, participants will be asked, "Have you had any new symptoms, injuries, illness or side effects or worsening of pre-existing conditions?" All adverse events (serious and non-serious; treatment-emergent and baseline-emergent; pharmacological treatment groups and intensive lifestyle intervention group) must be recorded on the DPP Adverse Event Report (Form E01).

8.2.1 Expedited Reports

For participants assigned to the pharmacological treatment groups, the NIDDK-NIH must provide a written report of all serious and unexpected adverse events to the Food and Drug Administration (FDA) within fifteen calendar days. If the event is a death or life-threatening and unexpected, the FDA must be notified by phone or fax within seven calendar days, followed by the written report within fifteen days.

In order to facilitate timely reporting of serious adverse events to the FDA by the NIDDK-NIH, the clinical center staff must contact the Coordinating Center (CoC) at the George Washington University Biostatistics Center immediately (301-881-9260) and fax (301-881-8752) the completed Adverse Event Report (Form E01) and the "initial" Serious Adverse Event Report (Form E02) prior to the close of the following business day. The serious adverse events will be monitored by the CoC's medical consultant. It is important to note that all serious and unexpected adverse events must be reported to the CoC, regardless of the intervention-related assessment. For example, a patient struck by lightning requires a report, even though this is not likely to be an intervention-related event.

The clinical center staff will not unmask the pharmacological treatment assignments. If, however, there is a serious adverse event which is thought by the clinical center staff to be possibly or probably related to the coded medication, the clinical center staff, when necessary for the safety of the participant, will contact the drug distribution center for pharmacological treatment assignment unmasking upon conferring with the clinical center's principal investigator. In this event, the clinical center staff must promptly contact the CoC with an explanation of the need for unmasking the pharmacological treatment assignment. A detailed written report must also be submitted to the CoC within three working days of the initial CoC contact.

9. DATA PROCESSING 9-1

9.1 Data Forms 9-1

9.1.1 Screening Period 9-1

9.1.2 Follow-up Period 9-1

9.1.3 Other Forms 9-2

9.1.4 Forms Completion During the Bridge Period 9-3

9.2 Remote Data Management System 9-3

9.2.1 Clinical Centers 9-3

9.2.2 Central Biochemistry Laboratory 9-3

9.3 Centralized Data Management System 9-3

9.4 Performance Monitoring 9-3

9.4.1 Training Workshop and Site Visits 9-3

9.4.2 Periodic Performance Reports 9-3

9.4.2.1 Recruitment and Retention 9-4

9.4.2.2 DPP Data Form Completion 9-4

9.4.2.3 Baseline Data 9-4

9.4.2.4 Other Reports 9-4

9.5 Interim Statistical Reports 9-4

9. DATA PROCESSING

9.1 Data Forms

DPP data forms are completed to document the eligibility of potential participants, to document protocol performance and to collect participant data relevant to the DPP primary and secondary research questions. The sections that follow outline the data forms contained in the DPP master data base maintained by the Coordinating Center (CoC).

9.1.1 Screening Period

The following data forms are completed during screening steps 2 through 4:

- **Eligibility Checklist (Form S01):**
  Documents the inclusion and exclusion criteria.
- **Screening Step 2 Inventory (Form S03):**
  BMI, arm blood pressures, urinalysis, current medications, and pregnancy/diabetes information; OGTT qualification, progression and local results; demographics and complete blood count (CBC) results.
- **Screening Step 3 Inventory - Start (Form S05):**
  History on family, weight, smoking, aspirin use, cardiovascular and stroke/TIA, other diseases/symptoms, diet, and medical history for women; anthropometric and ankle/arm systolic blood pressure; dispensing of medication for run-in.
- **Screening Step 3 Inventory - End (Form S06):**
  Run-in compliance and adverse events assessment; personal and socioeconomic information.
- **Screening Step 4 Inventory - Randomization (Form S07):**
  Adverse event assessment, pregnancy test result and current prescription medications; final eligibility review, micro-computer randomization and dispensing of coded medication.

9.1.2 Follow-up Period

The following data forms are completed after the randomization visit (screening step 4):

- **Standard Follow-up Visit Inventory (Form F01):**
  Completed every 3 months (except for annual follow-up visits): adverse event assessment, pregnancy questions, and current concomitant prescription medications; for the pharmacological treatment participants, coded medication compliance and dispensing, and at the end-month 6 visit only, CBC results. At mid-year follow-up visits (end-months 6, 18, ...): weight and arm blood pressures.
- **Major Follow-up Visit Inventory (Form F02):**
  Completed during annual follow-up visits (end-year 1, end-year 2, ...), end of study and at the time of the primary outcome (confirmed development of diabetes): weight, waist circumference, hip girth, and arm blood pressures; adverse event assessment, pregnancy questions and current concomitant prescription medications; for the pharmacological treatment participants, coded medication compliance and dispensing, and CBC results. At end-year 1 and end of study: skin-fold thickness and sagittal diameter. At end-year 3: ankle/arm systolic blood pressure.
- **Interim Follow-up Visit Inventory (Form F03):**
  Completed during coded medication titration visits and follow-up visits when **Form F01** and **Form F02** are not required: reason for interim visit, adverse event assessment, and for the pharmacological treatment participants, coded medication compliance and dispensing.
- **Missed Follow-up Visit Report (Form F04):**
  Completed anytime a participant misses either a standard or a major scheduled follow-up visit: reason for missed visit and inactive follow-up status.
- **Medication Adherence Interview (Form F05):**
  Completed anytime medication adherence is assessed on the Standard (Form F01), Major (Form F02) or Interim (Form F03) Follow-up Visit Inventory (Month 1 only).

- **Home Visit (Form F06)**

Completed during mid-year or annual home visits for an inactive participants: adverse event assessment, current concomitant prescription medications.

For participants in the intensive lifestyle intervention group, the following data forms are completed:

- **Exercise Stress Test Result (Form L01):**
  Completed at or soon after randomization to document exercise stress test results prior to initiating the intensive lifestyle intervention. Includes qualification and exercise tolerance test (ETT) results.
- **Lifestyle Contact - Telephone (Form L02):**
  Completed for all telephone contacts that involve DPP staff other than a secretary/receptionist. Includes duration and primary purpose of contact.
- **Lifestyle Contact - In Person (Form L03):**
  Completed for all in-person contacts. Includes nature of session (type and duration), self-monitoring information, and physical activity and weight status.
- **Lifestyle Physical Activity Log (Form L04):**
  Completed for each supervised physical activity session. Includes type of class and participants.
- **Lifestyle Group Session Log (Form L05):**
  Completed for each group session. Includes type of session and participants.

9.1.3 Other Forms

The following questionnaires are completed during the screening period and the follow-up period (see Section 12 for frequency of administration):

- Beck Questionnaires (Form Q01)
- MOS SF-36 Health Survey Questionnaire (Form Q02)
- Modifiable Activity Questionnaire (Form Q03)
- Low Level Physical Activity Recall (Form Q04)
- NHANES III Physical Activity Scale (Form Q05)
- Retention and Treatment Monitoring Measures (Form Q06)
- Nutrition Interview (Form Q07)
- Interval History Questionnaire (Form Q08)
- DPP-Specific Support Measure - Baseline Visit (Form Q09)
- DPP-Specific Support Measure - Follow-up Visits (Form Q10)
- Quality of Well-Being Scale (Form Q11)
- Economic Evaluation Questionnaire (Form Q12)
- Urinary Incontinence Questionnaire (Form Q13)
- End of Study Questionnaire (Form Q14)

The following event data forms are completed as needed:

- Adverse Event Report (Form E01)
- Serious Adverse Event Report (Form E02)
- Diabetes Confirmation Report (Form E03)
- Pregnancy Confirmation Report (Form E04)
- Pregnancy Outcome Report (Form E05)
- Mortality Event Report (Form E06)

During the screening period and the follow-up period, the following worksheets are completed during the conduct of the respective procedure (see Section 12 for frequency of procedures):

- OGTT Procedure - Follow-up Visits (Form P01)
- ECG Procedure (Form P02)
- Carotid Ultrasound Procedure (Form P03)
- Adiposity Substudy Procedure (Form P04)
- Re-screening OGTT Procedure (Form P05)

During the screening period and the follow-up period, the following report data forms are completed as needed:

- CHD Risk Status Report (Form R04)

The DPP data forms are created centrally at the CoC and sent to the clinics. The program coordinator reviews completed data forms prior to data entry. Completed forms are edited as they are entered into the microcomputer-based remote data management system.

9.1.4 Forms Completion During the Bridge Period

Changes to forms completion during the bridge period are to be kept to a minimum, using versions of the data forms listed in the protocol.

9.2 Remote Data Management System

9.2.1 Clinical Centers

The microcomputer-based remote data management system consists of a network of microcomputers, one at each clinical center and one at the CoC. Hardware includes microcomputers, laser printers and auto-answer, auto-dial modems. Data entry software corresponding to the DPP data forms completed at a clinical center is developed and maintained by the staff at the CoC. Data is entered by clinical center staff and transmitted weekly via telecommunications link to the CoC.

9.2.2 Central Biochemistry Laboratory

A single hard disk drive is dedicated to DPP data storage and transmission within the Central Biochemistry Laboratory's (CBL) microcomputer system. Data capture software corresponding to the analyses performed at the CBL is developed and maintained by the staff at the CoC. Data are downloaded from the CBL local area network to the DPP disk by CBL staff and transmitted daily via direct telecommunications link to the CoC.

9.3 Centralized Data Management System

Data is transmitted weekly from the clinical centers and daily from the CBL to the CoC, uploaded to the CoC's computer system, and converted to SAS data sets. All new data is edited for unavailable, out of range, or inconsistent values. Monthly audit programs will produce more detailed edits across all forms for an individual participant. Summaries are prepared for reports to the Steering Committee.

9.4 Performance Monitoring

9.4.1 Training Workshop and Site Visits

The CoC and the Lifestyle Resource Core (LRC) will establish procedures to train and certify clinical investigators in the DPP protocol and data processing procedures. Prior to the initiation of recruitment, workshops will be held at which time the personnel from the clinical centers will be trained in the appropriate DPP procedures including the use of the DPP data forms and data processing systems. At that time, CBL personnel will instruct the program coordinators on proper packaging and mailing of specimens for analysis by the CBL. The CoC and the LRC will maintain close contact with the program coordinators and will provide additional training or review as needed.

In addition to the training workshops, each of the clinical centers will be visited by clinical monitors. Appropriate representatives from the CoC, the LRC, the CBL, the NIDDK, and other experts will also visit the clinical centers, as required. These site visits will review procedures with the program coordinators/technicians, assess proficiency in executing the DPP protocol, review deficiencies detected in monitoring the performance of the clinical centers, review the utilization of personnel relative to the amounts budgeted, and receive feedback on the adequacy of the centralized support operations.

9.4.2 Periodic Performance Reports

During the DPP, the CoC will monitor the performance of the clinical centers and produce periodic reports summarizing protocol performance.

9.4.2.1 Recruitment and Retention

The performance of the clinical centers in recruiting and retaining eligible DPP participants will be carefully monitored by the CoC and the Recruitment and Retention Subcommittee. Monthly recruitment summaries will be issued to the Steering Committee throughout the recruitment and randomization period showing the number of potential participants screened, number randomized, and reasons for ineligibility or refusal to participate by clinical center. The reasons for ineligibility will be compared among the clinical centers, and if large differences are found, explanations for these differences will be sought. If the randomization rate falls below the desired number of participants per month, data on reasons for ineligibility and refusal will be used to try to identify strategies that would increase the randomization rate. The CoC will also prepare monthly reports on participant compliance with the DPP protocol and participants on inactive follow-up.

9.4.2.2 DPP Data Form Completion

The CoC will prepare quarterly reports presenting tabulations for the number of forms received, the number of edit messages, and the number of overdue forms and responses to edits. Missing data, particularly on outcome variables, will effectively reduce the power of analyses. In fact, systematic patterns of missing data could bias the results of the DPP. Therefore, many of the procedural details outlined in the Manual of Operations are designed to minimize the amount of missing data. The item-specific missing value rate on each DPP data form will be monitored throughout the DPP.

9.4.2.3 Baseline Data

The CoC will prepare quarterly reports consisting of comparisons between the treatment groups and clinical centers in the characteristics of the participants. If differences are found among the clinical centers, procedures at each center will be reviewed to attempt to distinguish between differences in the participant population and differences caused by variation in the participant selection or measurement techniques.

9.4.2.4 Other Reports

Other reports will be developed, as needed, based on requests from the Steering Committee and associated subcommittees.

9.5 Interim Statistical Reports

Unmasked interim analysis of the master data base will be presented to the Data Monitoring Board (DMB) at periodic intervals during the DPP. These confidential reports present all data relevant to an assessment of the DPP progress including protocol performance, effectiveness or ineffectiveness of the interventions and participant safety.

10. STATISTICAL CONSIDERATIONS 10-1
10.1 Data Relevant to the Primary Outcome 10-1
10.2 Sample Size Requirement 10-1
10.3 Statistical Analysis Plan 10-1
10.3.1 Washout Analysis 10-2
10.4 Interim Analysis Methods 10-2

10. STATISTICAL CONSIDERATIONS

10.1 Data Relevant to the Primary Outcome

The primary outcome for the DPP is conversion from impaired glucose tolerance to diabetes. Several published studies have examined the rates of conversion from IGT to diabetes. Twenty-one studies were identified that allowed computation of the participant-years (PY) of exposure and the associated actuarial conversion rates from IGT to diabetes, that is the number converting to diabetes divided by the PY of exposure. Overall, the conversion rates ranged from 2.3 per 100 PY (2.3% per year) among Japanese populations, 3 per 100 PY for Caucasians and Mexican-Americans, 4.7 per 100 PY for Naurians, 4.0 per 100 PY for women with a history of gestational diabetes, and between 10 and 11 per 100 PY for Asian Indians and Pima Indians (Yudkin et al. 1990, O'Sullivan 1991).

In addition to the published data, datasets from six population-based cohorts were obtained by the DPP coordinating center in order to analyze conversion rates from IGT to diabetes. In particular, conversion rates were computed for varying levels of fasting glucose at the time of IGT (Edelstein et al, 1997). The overall conversion rate for the six studies combined was 5.84 per 100 person-years of follow-up for all participants with IGT, and was 8.24 per 100 person-years for the participants with fasting glucose above 100 mg/dL. To decrease the sample size required to exhibit an effective treatment to prevent the onset of diabetes, the criteria of IGT with an elevated fasting glucose of 95 - 125 mg/dL was chosen for eligibility in the DPP. As these datasets were heavily weighted by populations at high risk of NIDDM, with relatively few Caucasians included, we anticipate a lower conversion rate of approximately 7.5% per year overall in those assigned to the control group of the DPP.

10.2 Sample Size Requirement

The following assumptions were used to determine the sample size goal of the DPP:

- Primary outcome is time to the confirmed development of diabetes.
- Eligible participants are uniformly randomized to one of the three treatment groups during a two and two-thirds year period and all randomized participants are followed for an additional three and one-third years after the close of randomization.
- Type I error rate (α) of 0.05 (two-sided) with a Bonferroni adjustment (Miller, 1981) for three pairwise comparisons of the three treatment groups.
- Control group's time to the development of diabetes is exponentially distributed with an diabetes development hazard rate of at least 0.065 per year.
- For participants assigned to the intervention groups (i.e., intensive lifestyle or metformin), the diabetes development hazard rate is reduced by at least 33%.

With these assumptions, the total effective sample size necessary to achieve 90% statistical power is 2,279 participants (Lachin and Foulkes, 1986). Assuming that randomized participants prematurely discontinue their follow-up visits prior to confirmed development of diabetes with an exponential loss hazard rate of 0.10 per year or less, the randomization goal of the DPP is 3,000 participants (1,000 per treatment group).

10.3 Statistical Analysis Plan

The principal analyses of primary and secondary outcomes will employ the "intent-to-treat" approach (Peduzzi, Wittes, et al., 1993). The intent-to-treat analyses will include all randomized participants with all participants included in their randomly assigned treatment group; treatment group assignment will not be altered based on the participant's adherence to the assigned treatment regimen. All statistical tests will be two-sided. The overall significance level of the primary outcome will be $\alpha = 0.05$. However, because interim analyses will be conducted throughout the DPP, the significance levels used in the interim and final analyses of the primary outcome will be adjusted to account for the multiplicity of interim analyses.

Baseline Characteristics. Comparison of the baseline characteristics among the three treatment groups will use standard nonparametric statistical techniques, such as Fisher's exact test for categorical data (Agresti, 1990), and the Kruskal-Wallis test for ordinal or continuous data (Conover, 1980).

Primary Outcome. The principal analysis of the DPP will be a lifetable analysis of the time to confirmed development of diabetes. Separate product-limit life-table estimated cumulative incidence curves will be calculated for each treatment group and the groups compared using a logrank test (Kalbfleisch and Prentice, 1980). For the primary outcome analysis, participants will be considered "administratively censored" if they complete the full duration of the DPP without confirmed development of diabetes. Participants who prematurely discontinue their follow-up visits prior to confirmed development of diabetes will be "censored" as of their last follow-up visit.

Mortality prior to the development of diabetes may be a competing risk event for the primary outcome (Lagakos, Lim and Robins, 1990). To account for mortality as a competing risk event, the treatment groups will be compared on the composite event defined as confirmed development of diabetes or all-cause mortality, whichever occurs first, using the same methods described above for the primary outcome.

Secondary Outcomes. Secondary time to “event” outcomes (e.g., mortality, cardiovascular morbidity) will be analyzed using the same lifetable methods described above for the primary outcome. A proportional hazards regression model will be used to evaluate potential covariables that may modify the primary and secondary time to event outcomes (e.g., risk population defined by race/ethnicity and history of GDM, baseline fasting and 2 hour glucose, clinical site). Graphical procedures will be used to assess the proportionality assumption. If the proportionality assumption is found to be unreasonable then other models such as the accelerated failure time model (Wei, 1992) or the proportional odds model (McCullagh, 1984) will be used to evaluate the covariables.

A lifetable analysis assesses the risk of the first event in time. Some processes, however, involve recurrent events such as back and forth between IGT and normal glucose tolerance. For these recurrent events, the family of statistical models based on the theory of counting processes will be applied (Fleming and Harrington, 1991).

Longitudinal data analysis techniques will be used to analyze repeated measures data (e.g., glycemia, fasting lipids, blood pressure, physical activity, quality of life). These include analyses of the point prevalence of a discrete characteristic (e.g. hypertension) at successive repeated visits over time (Lachin and Wei, 1988); multivariate rank analyses of quantitative (2 hour OGTT) or ordinal (MOS-36 Health Survey score) measures over successive visits (Wei and Lachin, 1984); the parametric linear random effects model of Laird and Ware (1982) to compare participant slopes over time (e.g., rate of change in fasting glucose) under a linearity and normality assumption; and techniques developed by Liang and Zeger (1986) to compare participant slopes under a generalized linear models framework.

10.3.1 Washout Analysis

The washout OGTT will be performed under different conditions than the other OGTTs in DPP, i.e. with study drug withheld. Furthermore, its timing relative to randomization will differ among participants (ranging from approximately 2-5 years after randomization). Thus, it will not be possible to analyze the washout as simply one additional data point in the same time-to-event (survival) analysis to be used for the main study. Instead, the washout OGTT (combined with a repeat test to confirm a new diagnosis) will be used in an analysis of prevalence of diabetes from randomization to the time of washout. The prevalence of diabetes will thus be determined by counting as cases all those diagnosed under the usual DPP follow-up procedure plus those diagnosed by the washout OGTT. The prevalence will be expressed as a simple percentage of all subjects enrolled and will be compared between metformin and placebo treatment groups. The analysis will be stratified by DPP study year of randomization, and the significance and homogeneity of the treatment effect over these strata will be assessed by standard statistical methods for stratified proportions data, such as the Mantel-Haenszel summary statistic and the Breslow-Day test for homogeneity over strata.

As a supplement to this analysis, we will also conduct the same analysis on the last OGTT results prior to washout. This is the appropriate analysis for comparative purposes rather than the time-to-event analysis of the DPP primary outcome, diabetes conversion without metformin “washout”.

10.4 Interim Analysis Methods

The Lan-DeMets (1983) spending function approach will be used to adjust the probability of a Type I error for testing the primary outcome when interim "looks" of the data are taken by the Data

Monitoring Board. The spending function corresponding to an O'Brien and Fleming (1979) boundary will be used. The Lan-DeMets procedure is flexible, in that the number of looks does not have to be specified in advance and the time interval between looks does not have to be the same throughout the DPP.

The rate at which the Type I error is spent is a function of the fraction of total information available at the time of the interim analysis (i.e., information time). For an interim analysis using the logrank test (i.e., time to confirmed development of diabetes), the information time is the fraction of the total number of confirmed diabetes events to be accrued in the entire DPP. Since the total number of events to be accrued is unknown, an estimate of the information time will be based on the fraction of total participant exposure (Lan and Lachin, 1990).

11. STUDY ADMINISTRATION 11-1

11.1 Organizational Units 11-1
11.1.1 Clinical Centers 11-1
11.1.2 Coordinating Center 11-1
11.1.3 NIDDK Project Office 11-1

11.2 Funding Mechanism/Study Resources 11-1

11.3 Working Committees 11-1
11.3.1 Steering Committee and Subcommittees 11-1
11.3.2 Data Monitoring Board 11-2

11.4 Policies 11-2
11.4.1 Publications 11-2
11.4.2 Ancillary Studies 11-3

11. STUDY ADMINISTRATION

11.1 Organizational Units

11.1.1 Clinical Centers

Each of the participating clinical centers have agreed to implement the DPP Protocol. The clinical centers will recruit eligible participants; follow participants according to protocol specified criteria; assume responsibility for the management of each participant enrolled in the DPP; record participant data related to all of the above; review and enter information from data forms using the remote data management system; and respond to edit queries from the Coordinating Center (CoC). Each clinical center has a Principal Investigator, a Program Coordinator, a Recruitment Coordinator, and additional staff to carry out the protocol that may include dietitians, behaviorists, exercise physiologists, physicians, data collectors, and others.

11.1.2 Coordinating Center

The Coordinating Center (CoC) is responsible for all aspects of biostatistical design, analysis, and data processing of the DPP. In collaboration with the Steering Committee, the CoC is responsible for document processing of the protocol and Manual of Operations and data collection forms development and testing. The CoC monitors protocol performance, and conducts the interim and final statistical analyses. The CoC collaborates with the Steering Committee members in the preparation of publications based on DPP results.

Central resource units include the Central Biochemistry Laboratory (CBL), Nutrition Coding Center (NCC), ECG Grading Center (ECG), Carotid Ultrasound Reading Center (CURC) and Quality of Well Being Coding Center (QWB). These units function as subcontracts to the CoC. They establish and provide baseline and repeated measures of study outcomes as described in sections 5 and 12; the CBL provides eligibility measures as well. Quality control systems are established for these centrally performed assessments and reports will be furnished periodically to the research group. In addition, the units will lend expertise to help formulate the protocol and detailed procedures for participant preparation, specimen and record labeling, handling and shipping. Communication systems are maintained for data transfer to the Coordinating Center.

11.1.3 NIDDK Project Office

The DPP will be funded as cooperative agreements among the clinical centers and the CoC. The NIDDK program officer will provide program involvement as a participant in the scientific efforts of the DPP Research Group through development of protocols and assistance in the conduct of the DPP.

11.2 Funding Mechanism/Study Resources

Funding for the DPP is provided by the NIH through the National Institute of Diabetes and Digestive and Kidney Diseases, the Office of Research on Minority Health, The National Institute of Child Health and Human Development, and The National Institute of Aging. In addition, The Indian Health Service, the Centers for Disease Control and Prevention, The American Diabetes Association, and two private concerns, Bristol-Myers Squibb and Parke-Davis are contributing support for the DPP. All support to the clinical centers and the Coordinating Center will be provided through the NIDDK using the mechanism of the Cooperative Agreement.

11.3 Working Committees

11.3.1 Steering Committee and Subcommittees

The Steering Committee (SC) is the representative body of the DPP research group. The Committee consists of the Principal Investigator from each clinical center and the CoC, and the NIDDK project manager. This committee is the policy and decision making group, and will oversee the administrative aspects of the DPP Research Group. It provides overall scientific direction for the DPP through consideration of recommendations from the subcommittees and Planning Committee. The committee will approve the details of study design, all procedure manuals and participant management policies, and assure that sufficient numbers of eligible participants are being entered into the DPP during the screening phase. The SC will monitor protocol adherence at the clinical centers including proper data generation, recording and transmittal. Members unable to attend a meeting may designate an alternate to act on their behalf. The Study Chairperson is selected by the members of the Steering Committee.

Steering Committee recommendations for changes in the Protocol require prior consideration by the appropriate subcommittee and Planning Committee, and an affirmative vote by two-thirds of the Steering Committee members present and voting.

Although the Steering Committee is the decision and policy making group of the DPP, a smaller group has been appointed to direct day-to-day activities. This committee consists of the Chair of the Steering Committee, the NIDDK Project Manager and the Director of the Division of Diabetic Endocrinology and Metabolic Disease (DEEMED), and the Principal Investigator and Project Coordinator of the CoC. The committee meets by telephone conference, as necessary, and generally on a weekly basis.

Subcommittees comprise members of the research group. Their function is to develop detailed policies and procedures and make recommendations to the Steering Committee. The chairpersons of the subcommittees are members of the Planning Committee which serves as the forum where the work of the subcommittees is initially reviewed and coordinated. The following subcommittees are active during the Planning Phase to develop the Protocol and detailed study procedures. During patient recruitment and follow-up there will be a reformation of the subcommittees as necessary to address the different tasks of the new Phase.

Planning Phase Subcommittees:

- Ancillary Studies
- Concomitant Conditions
- Intervention
- Outcomes
- Publications and Presentations
- Program Coordinator
- Recruitment/Retention
- Screening/Eligibility

11.3.2 Data Monitoring Board

The Data Monitoring Board (DMB) members will serve as external reviewers and advisors to the NIDDK-NIH, and the Steering Committee. The DMB will consist of experts in relevant biomedical fields, biostatistics and medical ethics. Prior to the initiation of the DPP, the DMB members will review all study material to ensure the scientific validity of the study and safety of participants entered into the DPP. The DMB will also assess the performance of the CoC and clinical centers. Its principal responsibility will be to monitor the emerging results of the DPP to assess treatment effectiveness, or ineffectiveness, and participant safety. Based on these considerations, the DMB may recommend to the NIDDK that the protocol be modified or that the DPP be terminated.

11.4 Policies

11.4.1 Publications

The Publications and Presentations Subcommittee (PPS) will coordinate, monitor, review, and assume responsibility for arranging the preparation of all study-wide communications (press releases, interviews, presentations, and publications) relating to the scientific aspects of the DPP. During the course of the DPP, there will be no publication or presentation of study plans or results which have not been reviewed and approved by a majority of the PPS, and for some types of communications, a majority of the Steering Committee.

With respect to publications and presentations from the DPP, the goals of the PPS are to:

1. ensure accurate, uniform, timely, and high quality reporting of the DPP activities and results;
2. preserve the scientific integrity of the study;
3. safeguard the rights and confidentiality of participants;
4. assure that the timing of publications and presentations serves the right of the public to know the results of the program without jeopardizing its conduct.

The PPS will organize a writing group for each publication or presentation proposed by the DPP investigators. Members of the writing group will include volunteers from the DPP investigators at large, and will not be restricted to members of the PPS. The PPS will coordinate the efforts of the writing group, establish priorities for data analysis by the Coordinating Center, and help edit the manuscripts produced by the writing groups.

There will be several categories of publications and presentations, with different rules for authorship, ranging from publications of the main results of the study (with authorship by the entire research group) to other types of publications with named authors. The authorship rules balance the need to recognize the contributions of all DPP investigators and staff with the need to recognize individuals for specific contributions to certain types of publications and presentations.

11.4.2 Ancillary Studies

The Ancillary Studies Subcommittee will evaluate proposals for studies that involve DPP participants and that are not a part of the DPP protocol. These studies will in general, be done only on a subset of participants in the DPP. However, studies that include all DPP participants and studies that analyze DPP data in ways extracurricular to the Protocol must be submitted to the Ancillary Studies Subcommittee. Ancillary studies will have to obtain funding from outside the DPP.

Major factors in approval of ancillary studies will include:

- compatibility of goals with those of DPP.
- should not place undue burden on DPP subjects and staff.

Ancillary studies will receive a primary, secondary and statistical review. An outside reviewer may be used if there is no expertise within the DPP in a specific area. Reviews will be returned to the applicant and appeals from the decision of the Ancillary Studies Subcommittee may be made to the Steering Committee.

12. SCHEDULE OF PROCEDURES 12-1
12.1 Eligibility Screening and Baseline Measurements 12-1
12.2 Outcomes, Safety Testing, and Measures for Adherence 12-2
12.2.1 Changes due to early termination of the masked treatment phase 12-5

12. SCHEDULE OF PROCEDURES

12.1 Eligibility Screening and Baseline Measurements

STEP	TEST OR PROCEDURE	COMMENTS
1 - Initial screen	Informed consent #1	
	Screening Glucose	Random and/or fasting fingerstick capillary glucose, read locally
	Screening Questionnaire	Initial review of eligibility criteria
	Height, Weight, Blood pressure	Initial screening for eligibility
2 - OGTT	Informed Consent #2	
	75 gram oral glucose tolerance test (OGTT)	Fingerstick capillary glucose at 0, 120 minute, read locally to determine if subject is eligible
		0, 30, 120 minute samples for glucose
		0, 30 minute insulin, 0 minute proinsulin
	electrolytes*, TSH*, triglyceride*, serum creatinine, ALT/AST, urine dipstick for protein*, CBC	Laboratory eligibility criteria<br><br>CBC and urine dipstick for protein
	Medical history & physical	Eligibility evaluation
3 - Run-in start	Informed Consent #3	
	Medical history/physical examination, medications	Baseline evaluation
	Creatinine Clearance	Only for participants who are or will become 80 years of age during the DPP.
	ECG	Read locally for safety, sent to ECG reading center for later reading if eligible
	Nutrient and physical activity questionnaires	
	Placebo dispensed and Diet and physical activity diaries	Initiate run-in
3 - Run-in end	Pill counting, diary evaluation, and Behavioral questionnaires	Run-in evaluation
4 - Baseline/ Randomization	Informed consent #4	
	Derived lipid profile, full beta-quant, ICA, GAD, HbA_{1c}, urine albumin, urine creatinine, fibrinogen, TPA, DNA storage, long term storage, LDL particle size, LDL-ApoB, LDL-cholesterol, HCG*, CRP, and carotid ultrasound	Baseline evaluations, not used for eligibility<br><br>Full Beta Quant should be done only if TG > 400 mg/dL.<br><br>HCG determined locally before randomization<br>Carotid ultrasound to be done before randomization.

* Laboratory specimens collected at baseline for eligibility

12.2 Outcomes, Safety Testing, and Measures for Adherence

	Time Req. (min.)	Baseline and End of Study	Month												Primary Outcome +
			03	06	12	18	24	30	36	42	48	54	60	66	
**Glycemia**															
Fasting Glucose		X		X	X	X	X	X	X	X	X	X	X	X	M
30', 120' Glucose	120	X			X		X		X		X		X		M
HbA_{1c}		X		X	X		X		X		X		X		M
**Insulin Secretion and Sensitivity**															
Fasting, 30' Insulin		X			X		X		X		X		X		M
Fasting Proinsulin		X			X		X		X		X		X		M
**Fibrinolysis and Clotting Factors**															
Fibrinogen, TPA		X			X										
CRP		X		X	X										
**Lipids (Fasting)**															
Derived Beta Quant		X		X	X		X		X		X		X		
Full Beta Quant (If ↑ TG)		X		X	X		X		X		X		X		
LDL Particle Size		X		X	X		X		X		X		X		
LDL - ApoB		X		X	X		X		X		X		X		
LDL - CH		X		X	X		X		X		X		X		
**Serologic IDDM Evidence**															
ICA & GAD Antibodies		X													X
**Kidney Function**															
Urine Albumin & Creatinine Concentration	5	X													X
**Saved Specimens**															
		X		X	X		X		X		X		X		

	Time Req. (min.)	Baseline and End of Study	Month												Primary Outcome +
			03	06	12	18	24	30	36	42	48	54	60	66	
**Physical**															
Height	5	X													
Weight	5	X		X	X	X	X	X	X	X	X	X	X	X	
Waist/Hip Circumf.	10	X			X		X		X		X		X		
Sagittal Diameter.	5	X			X										
Skin-fold	10	X			X										
**Blood Pressure**															
Arm BP	10	X		X	X	X	X	X	X	X	X	X	X	X	
ABI	20	X			X				X						
**Quality of Life**															
Beck Anxiety & Depression Inventories	10	X			X		X		X		X		X		
MOS SF-36	10	X			X		X		X		X		X		
Quality of Well-Being Scale	10 - 15	X			X		X		X		X		X		
**Adherence Measures**															
Retention & Treatment Monitoring	20	X		X		X		X		X		X		X	
DPP-Specific Social Support	5	X			X		X		X		X		X		
Medication Adherence Interview*◆	15	X	X	X	X	X	X	X	X	X	X	X	X	X	
**Physical Activity & Nutrition**															
Modifiable Activity Questionnaire	10	X			X		X		X		X		X		
Low Level Physical Activity Recall	10	X		X	X		X		X		X		X		
Nutrient Intake	25	X			X										
**Cardiovascular**															
ECG	15	X			X		X		X		X		X		
Symptom History	10	X			X		X		X		X		X		
Carotid Ultrasound	45	X													

	Time Req. (min.)	Baseline and End of Study	Month												Primary Outcome +
			03	06	12	18	24	30	36	42	48	54	60	66	
**Other Outcomes**															
Interval History Questionnaire	15	X			X		X		X		X		X		
**Safety Testing**															
CBC (hemoglobin, hematocrit, platelets)[*]		X		X	X		X		X		X		X		
Blood Chemistries[**]		X	X	X	X	X	X	X	X	X	X	X	X	X	
Adverse Event Report[***]		X	X	X	X	X	X	X	X	X	X	X	X	X	X
Pregnancy #															

+ For the Primary Outcome Visit, items marked M need to be collected only if fasting plasma glucoses (i.e., no OGTTs) were collected to determine diabetes.

[*] In the pharmacological treatment groups.

◆ The Medication Adherence Interview will be completed at the month 1 visit and then at Standard (Form F01) and Major (Form F02) follow-up visits.

[**] In the pharmacological treatment groups, for liver function, liver enzymes (ALT-SGPT, AST-SGOT) and for kidney function, serum creatinine will be collected every 6 months. In addition, liver enzymes will be collected at the month 3 visit.

[***] Adverse events will be collected at Standard (Form F01), Major (Form F02), and Interim (F03) follow-up visits.

Pregnancy tests as needed based on symptoms and menstrual history.

Note: CBC and pregnancy tests are local measures.

12.2.1 Changes due to early termination of the masked treatment phase

Data will be collected as specified in protocol version 4.2 with the following modifications.

a. Scheduled “End of study visits” (as noted in Table 12.2) will not be conducted due to early termination of DPP.
b. A urine specimen to measure urine albumin and creatinine concentration will be collected.
c. Carotid ultrasound may be conducted at a visit to be scheduled.
d. Questions concerning urinary incontinence (Q13), and questions concerning barriers, support and medication assignment (Q14) in the DPP will be asked.

12.2.2 Schedule of procedures changed for bridge period

Pregnancy tests, CBC, liver enzymes and serum creatinine will not be collected for participants formerly assigned to placebo, troglitazone, ILS, or that no longer take metformin. Safety laboratory tests will be drawn on participants continuing to take metformin. The medication adherence interview will be completed at mid-year and annual visit only on persons taking metformin.

13. STUDY TIMETABLE.. 13-1

13. STUDY TIMETABLE

**Phase I**	July 1994 - June 1996	Protocol Development and Implementation
	July 1994 - December 1995	Protocol Development
	January 1996 - June 1996	Protocol Implementation
**Phase II**	July 1996 - June 2002	Participant Randomization and Follow-up
	July 1996 - June 1999	Recruitment and Follow-up
	July 1999 – December 2001	Participant Follow-up
**Phase III**	June 2001	Initiate Study Close-out and Data Analysis
**Phase IV**	January 1, 2002	Bridge Period Starts
	No later than December 31, 2002	Bridge Period Ends
**Study End**	December 31, 2002	Participant Contact Ends
	June 30, 2003	End of Final DPP Funding Cycle

14. BIBLIOGRAPHY.. 14-1
14.1 Section 14.1 Bibliography for Background to the Washout Study................................ 14-7

14. BIBLIOGRAPHY

Adverse reactions to bendrofluazide and propranolol for the treatment of mild hypertension. Report of Medical Research Council Working Party on Mild to Moderate Hypertension. Lancet, 2: 539-543, 1981.

Agresti A. Categorical Data Analysis, New York, John Wiley 1990.

American Diabetes Association. Detection and management of lipid disorders in diabetes. Diabetes Care, 16:828-834, 1993.

American Diabetes Association. Nutritional recommendations and principles for individuals with diabetes mellitus. Diabetes Care, 18 (Suppl 1): 16-19, 1995.

American Diabetes Association. Report of the expert committee on the diagnosis and classification of diabetes mellitus. Diabetes Care, 20 (7), 1997.

American Diabetes Association. Screening for diabetes. Diabetes Care, 13:7-9, 1990.

Annuzzi G, Vaccaro O, Caprio S, Di Bonito P, Caso P, Riccardi G, Rivellese A. Association between low habitual physical activity and impaired glucose tolerance. Clinical Physiology, 5:63-70, 1985.

Banerji MA, Lebovitz HE. Coronary heart disease risk factor profiles in black patients with non-insulin-dependent diabetes mellitus: paradoxic patterns. American Journal of Medicine, 91:51-59, 1991.

Banerji MA, Norin AJ, Chaiken RL, Lebovitz HE. HLA-DQ associations distinguish insulin-resistant and insulin-sensitive variants of NIDDM in black Americans. Diabetes Care, 16:429-433, 1993.

Barrett-Connor E. Epidemiology, obesity and non-insulin-dependent diabetes mellitus. Epidemiology Review, 11:172-181, 1989.

Barrett-Connor EL; Cohn BA; Wingard DL; Edelstein SL. Why is diabetes mellitus a stronger risk factor for fatal ischemic heart disease in women than in men? JAMA 1991 265:627-631, 1991.

Bengtsson C, Blohme G, Lapidus L, Lindquist O, Lundgren H, Nystrom E, Petersen K, Sigurdsson JA. Do antihypertensive drugs precipitate diabetes? British Medical Journal (Clin Res Ed), 289: 1495-1497, 1984.

Bergstrom RW, Newell-Morris LL, Leonetti DL, Shuman WP, Wahl PW, Fujimoto WY. Association of elevated fasting c-peptide level and increased intra-abdominal fat distribution with the development of NIDDM in Japanese-American men. Diabetes, 39:104-111, 1990.

Birmingham Diabetes Survey Working Party. Ten year follow-up report on Birmingham diabetes survey of 1961. British Medical Journal, 35-37, 1976.

Bourn DM, Mann JI, McSkimming BJ, Waldron MA, Wishart JD. Impaired glucose tolerance and NIDDM: does lifestyle intervention program have an effect. Diabetes Care, 17:1311-1319, 1994.

Brunzell JD, Robertson RP, Lerner RL, Hazzard, WR, Ensinck JW, Bierman EL, Porte D Jr. Relationships between fasting plasma glucose levels and insulin secretion during intravenous glucose tolerance tests. Journal of Clinical Endocrinology and Metabolism, 42:222-229, 1976.

Burchfiel CM, Curb JD, Rodriguez BL, Yano K, Hwang LJ, Fong KO, Marcus EB. Incidence and predictors of diabetes in Japanese-American men. The Honolulu Heart Program. Annals of Epidemiology, 5:33-43, 1995.

Chaiken RL, Banerji MA, Huey H, Lebovitz HE. Do blacks with NIDDM have an insulin-resistance syndrome. Diabetes, 42:444-449, 1993.

Chiasson JL, Josse RG, Hunt JA, Palmason C, Rodger NW, Ross SA, Ryan EA, Tan MH, Wolever, TM. The efficacy of acarbose in the treatment of patients with non-insulin-dependent diabetes mellitus. Annals of Internal Medicine, 121:928-935, 1994.

Colditz GA, Willett WC, Stampfer MJ, Manson JE, Hennekens CH, Arky RA, Speizer FE. Weight as a risk factor for clinical diabetes in women. American Journal of Epidemiology, 132:501-513, 1990.

Conover WJ. Practical Nonparametric Statistics, New York, Wiley, 1980.

Coustan DR, Carpenter MW, O'Sullivan PS, Carr SR. Gestational diabetes: predictors of subsequent disordered glucose metabolism. American Journal Obstetrics and Gynecology, 168:1139-1144, 1993.

Cowie CC, Harris MI, Silverman RE, Johnson EW, Rust KF. Effect of multiple risk factors on differences between blacks and whites in the prevalence of non-insulin-dependent diabetes mellitus in the United States. American Journal of Epidemiology, 137:719-732, 1993.

Cowie CC, Port FK, Wolfe RA, Savage PJ, Moll PP, Hawthorne VM. Disparities in incidence of diabetic end-stage renal disease according to race and type of diabetes. The New England Journal of Medicine, 321:1074-1079, 1989.

Curb JD, Aluli NE, Kautz JA, Petrovitch H, Knutsen SF, Knutsen R, O'Conner HK, O'Conner WE. Cardiovascular risk factor levels in ethnic Hawaiians. American Journal of Public Health, 81:164-167, 1991.

Damm P, Kuhl C, Hornnes P, Molsted-Perersen L. A longitudinal study of plasma insulin and glucose in women with previous gestational diabetes. Diabetes Care, 18:654-665, 1995.

DeFronzo RA, Goodman AM. Efficacy of metformin in patients with non-insulin-dependent diabetes mellitus. The Multicenter Metformin Study Group. New England Journal of Medicine, 333:541-549, 1995.

DeFronzo RA. The triumvirate: B-cell, muscle, liver: a collusion responsible for NIDDM. Diabetes, 37:667-687, 1988.

Denno KM, Sadler TW. Effects of the biguanide class of oral hypoglycemic agents on mouse embryogenesis. Teratology, 49:260-266, 1994.

Doar JWH, Wilde CE, Thompson ME, Sewell PFJ. Influence of treatment with diet alone on oral glucose-tolerance test and plasma sugar and insulin levels in patients with maturity-onset diabetes mellitus. Lancet, 1: 1263-6, 1975.

Dowse GK, Zimmet PZ, Gareeboo H, Alberti KG, Tuomilehto J, Finch CF, Chitson P, Tulsidas H. Abdominal obesity and physical inactivity as risk factors for NIDDM and impaired glucose tolerance in Indian, Creole, and Chinese Mauritians. Diabetes Care, 14:271-282, 1991.

Edelstein SL, Knowler WC, Bain RP, Andres R, Barrett-Connor EL, Dowse GK, Haffner SM, Pettitt DJ, Sorkin JD, Muller DC, Collins VR, and Hamman RF. Predictors of Progression from Impaired Glucose Tolerance to NIDDM: An Analysis of Six Prospective Studies. Diabetes, 46:701-710, 1997.

Eriksson J, Franssila-Kallunki A, Ekstrand A, Saloranta C, Widen E, Schalin C, Groop L. Early metabolic defects in persons at increased risk for non-insulin-dependent diabetes mellitus. The New England Journal Medicine, 321:337-43, 1989.

Eriksson KF, Lindgarde F. Prevention of Type II (non-insulin-dependent) diabetes mellitus by diet and physical exercise: the 6-year Malmo feasibility study. Diabetologia, 34:891-898, 1991.

Eriksson KF, Saltin B, Lindgarde F. Increased skeletal muscle capillary density precedes diabetes development in men with impaired glucose tolerance. Diabetes, 43:805-808, 1994.

Eschwege E, Richard JL, Thibult N, Ducimetiere P, Warnet JM, Claude JR, Rosselin GE. Coronary heart disease mortality in relation with diabetes, blood glucose and plasma insulin levels. The Paris Prospective Study, ten years later. Hormone and Metabolism Research, 15 (Suppl):41-46, 1985.

Everhart JE, Knowler WC, Bennett PH. Incidence and risk factors for non-insulin-dependent diabetes. In: National Diabetes Data Group. Diabetes in America: Washington, D.C.: US GPO, 1985(NIH publication no 85-1468).

Flegal KM, Ezzati TM, Harris MI, Haynes SG, Juarez RZ, Knowler WC, Perez-Stable EJ, Stern MP. Prevalence of diabetes in Mexican Americans, Cubans, and Puerto Ricans from the Hispanic Health and Nutrition Examination Survey, 1982-1984. Diabetes Care, 14:628-638, 1991.

Flemming TR, Harrington DP. Counting process and survival analysis. New York: Wiley, 1991.

Fujimoto WY, Leonetti DL, Kinyoun JL, Shuman WP, Stolov WC, Wahl PW. Prevalence of complications among second-generations Japanese-American men with diabetes, impaired glucose tolerance, or normal glucose tolerance. Diabetes, 36:730-739, 1987.

Fujimoto WY. Diabetes in Asian and Pacific Islander Americans. In: Harris, MI (ed.), Diabetes in America, NIH, Washington, DC, 1995, pp. 661-681.

Fuller JH, Shipley MJ, Rose G, Jarrett RJ, Keen H. Coronary-heart-disease risk and impaired glucose tolerance. The Whitehall study. Lancet, 1: 1373-1376, 1980.

Garancini MP, Calori G, Ruotolo G, Manara E, Izzo A, Ebbli E, Bozzetti AM Boari L, Lazzari P, Gallus G. Prevalence of NIDDM and impaired glucose tolerance in Italy: an OGTT-based population study. Diabetologia, 38:306-313, 1995.

Geiss LS, Herman WH, Goldschmid MG, DeStefano. Surveillance for Diabetes Mellitus - United States, 1980-1989. Morbidity and Mortality Weekly Report, 42:1-19, 1993.

Glynn, Thomas J.; Manley, Marc. How to help your patients stop smoking/G. : a National Cancer Institute manual for physicians. [Bethesda, Md.] :/Smoking, Tobacco, and Cancer Program, Division of Cancer Prevention and Control, National Cancer Institute, U.S. Dept. of Health and Human Services, Public Health Service, National Institutes of Health,/1989. (NIH publication ;/89-3064/G).

Grant PT, Oats JN Beischer NA. The long-term follow-up of women with gestational diabetes. Austrian and New Zealand Journal Obstetrics and Gynecology, 16:17-22, 1986.

Gray DS, Fujioka K, Devine W, Bray GA. A randomized double-blind clinical trial of fluoxetine in obese diabetics. International Journal of Obesity and Related Metabolic Disorders, 16 (Suppl 4):S67-72, 1992.

Guidelines for perinatal care./N. 2nd ed. ed. Elk Grove Village, Ill. :/American Academy of Pediatrics ; Washington, DC : American College of Obstetricians and Gynecologists,/c1988.

Haffner SM, Hazuda HP, Mitchell BD, Patterson JK, and Stern MP. Increased incidence of Type II diabetes mellitus in Mexican Americans. Diabetes Care, 14:102-108, 1991.

Haffner SM, Stern MP, Hazuda HP, Mitchell BD, Patterson JK. Cardiovascular risk factors in confirmed prediabetic individuals: does the clock for coronary heart disease start ticking before the onset of clinical diabetes? JAMA, 263:2893-2898, 1990.

Haffner SM, Stern MP, Hazuda HP, Pugh JA, Patterson JK. Do upper body and centralized adiposity measure different aspects of body fat distribution? Relationship to non-insulin dependent diabetes mellitus, lipids and lipoproteins. Diabetes, 36:43-51, 1987.

Haffner SM, Valdez RA, Hazuda HP, Mitchell BD, Morales PA, Stern MP. Prospective analysis of the insulin resistance syndrome (Syndrome X). Diabetes, 41:715-722, 1992.

Hanson RL, Pettitt DJ, Bennett PH, Narayan KM, Fernandes R, de Courten M, Knowler WC. Familial relationships between obesity and NIDDM. Diabetes, 44:418-422, 1995.

Harris MI, Hadden WC, Knowler WC, and Bennett PH. Prevalence of diabetes and impaired glucose tolerance and plasma glucose levels in U.S. population aged 20-74 years. Diabetes, 36:523-534, 1987.

Harris MI. Impaired glucose tolerance in the U.S. population. Diabetes Care, 12:464-474, 1989.

Harris MI. Non-insulin-dependent diabetes mellitus in black and white Americans. Diabetes/Metabolism Reviews, 6:71-90, 1990.

Harris MI. Epidemiological correlates of NIDDM in Hispanics, Whites and Blacks in the U.S. population. Diabetes Care, 14:639-648, 1991.

Helmrich SP, Ragland DR, Leung RW, Paffenbarger RS. Physical activity and reduced occurrence of non-insulin-dependent diabetes mellitus. The New England Journal of Medicine, 325:147-152, 1991.

Henry OA, Beischer NS. Long-term implications of gestational diabetes for the mother. Bailliere's Clinical Obstetrics and Gynecology, 5:461-483,1991.

Hoy WE; Megill DM; Hughson MD. Epidemic renal disease of unknown etiology in the Zuni Indians. American Journal of Kidney Diseases, 9:485-496, 1987.

Hypertension Prevention Trial Research Group. The Hypertension Prevention Trial: three-year effects of dietary changes on blood pressure. Archives of Internal Medicine, 150:153-162, 1990.

Jackson WPV. Diagnosis and dietary management of mild and borderline diabetes. In Proceedings of the VIII Congress, Malaise WJ, ed. Amsterdam Excerpta Medica, 1974, p. 639.

Jarrett RJ, Keen H, Fuller H, and McCartney M. Worsening to diabetes in men with impaired glucose tolerance ("Borderline Diabetes"). Diabetologia, 16:25-30, 1979.

Jarrett RJ. Epidemiology and public health aspects of non-insulin-dependent diabetes mellitus. Epidemiological Review, 11:151-171, 1989.

Kadowaki T, Miyake Y, Hagura R, Akanuma Y, Kajinuma H, Kuzuya N, Takaku F, Kosaka K. Risk factors for worsening to diabetes in subjects with impaired glucose tolerance. Diabetologia, 26:44-49, 1984.

Kalbfleisch JD, Prentice RL. The Statistical Analysis of Failure Time Data. New York: John Wiley, 1980.

Kannel, WB, McGee DL. Diabetes and glucose tolerance as risk factors for cardiovascular disease: the Framingham study. Diabetes Care, 2: 120-6, 1979.

Kawate R, Yamakido M, Nishimoto Y, Bennett PH, Hamman RF, Knowler WC. Diabetes mellitus and its vascular complications in Japanese migrants on the Island of Hawaii. Diabetes Care, 2:161-170, 1979.

Keen H, Jarrett RJ, McCartney P. The ten-year follow-up of the Bedford Survey (1962-1972): Glucose tolerance and diabetes. Diabetologia, 22:73-78, 1982.

King H, Rewers M, and the WHO Ad Hoc Diabetes Reporting Group. Global estimates for prevalence of diabetes mellitus and impaired glucose tolerance in adults. Diabetes Care, 16:157-177, 1993.

King H, Rewers M. Diabetes in adults is now a Third World problem. The WHO AD Hoc Diabetes Reporting Group. Bulletin World Health Organization, 69:643-648, 1991.

King H, Zimmet P, Raper LR, Balkau B. Risk factors for diabetes in three Pacific populations. American Journal of Epidemiology, 119: 396-409, 1984.

Kjos SL, Peters RK, Xiang A, Henry OA, Montoro M, Buchanan TA. Predicting future diabetes in Latino women with gestational diabetes. Utility of early postpartum glucose tolerance testing. Diabetes, 44:586-591, 1995.

Klein R, Klein BEK, Linton KLP, Moss SE. Microalbuminuria in a population-based study of diabetes. Archives of Internal Medicine, 152:153-158, 1992.

Knowler WC, Bennett PH, Hamman RF, Miller M. Diabetes incidence and prevalence in Pima Indians: a 19-fold greater incidence than in Rochester, Minnesota. American Journal of Epidemiology, 108:497-505, 1978.

Knowler WC, Pettit DJ, Savage PJ, Bennett PH. Diabetes incidence in Pima Indians: contributions of obesity an parental diabetes. American Journal of Epidemiology, 113:144-156, 1981.

Kovar MG, Harris MI, and Hadden WC. The scope of diabetes in the United States population. American Journal of Public Health, 77:1549-1550, 1987.

Kumanyika SK, Obarzanek E, Stevens VJ, Herbert PR, Whelton PK. Weight-loss experience of black and white participants in NHLBI-sponsored clinical trials. American Journal of Clinical Nutrition, 53:1631S-1638S, 1991.

Lachin JM and Foulkes MA. Evaluation of sample size and power for analyses of survival with allowance for nonuniform patient entry, losses to follow-up, noncompliance, and stratification. Biometrics, 42:509-519, 1986.

Lachin JM, Wei LJ. Estimators and tests in the analysis of multiple nonindependent 2 x 2 tables with partially missing observations. Biometrics, 44: 513-28, 1988.

Lagakos SW, Lim LL, Robins JM. Adjusting for early treatment termination in comparative clinical trials. Statistics in Medicine, 9:1417-1424, 1990.

Laird NM, Ware JH. Random-effects models for longitudinal data. Biometrics, 38:963-74, 1982.

Lam KS, Li DF, Lauder IJ, Lee CP, Kung AWC, Ma JTC. Prediction of persistent carbohydrate intolerance in patients with gestational diabetes. Diabetes Research and Clinical Practice, 12:181-186, 1991.

Lan KK, Lachin JM. Implementation of group sequential logrank tests in a maximum duration trial. Biometrics, 46:759-70, 1990.

Lan KK and DeMets DL. Discrete sequential boundaries for clinical trials. Biometrika, 64:191-199, 1983.

Leahy JL. Natural history of b-cell dysfunction in NIDDM. Diabetes Care, 13:992-1010, 1990.

Lee ET, Howard BV, Savage PJ, Cowan LD, Fabsitz RR, Oopik AJ, Yeh J, Go O, Robbins DC, Welty TK. Diabetes and impaired glucose tolerance in three American Indian populations aged 45-74 years. Diabetes Care, 18:599-610, 1995.

Levine LR, Enas GG, Thompson WL, Byyny RL, Dauer AD, Kirby RW, Kreindler TG, Levy B, Lucas CP, McIlwain HH, et al. Use of fluoxetine, a selective serotonin-uptake inhibitor, in the treatment of obesity: a dose-response study. International Journal of Obesity, 13: 635-645, 1989.

Liang K, Zeger SL. Longitudinal data analysis using generalized linear models, Biometrika, 73:13-22, 1986.

Long SD, O'Brien K, MacDonald KG, Leggett-Frazier N, Swanson MS, Pories WJ, Caro JF. Weight loss in severely obese subjects prevents the progression of impaired glucose tolerance to type II diabetes. Diabetes Care, 17:372-375, 1994.

Manson JE, Nathan DM, Krolewski AS, Stampfer MJ, Willett WC, and Hennekens CH. A prospective study of exercise and incidence of diabetes among US male physicians. JAMA, 268:63-67, 1992.

Manson JE, Rimm EB, Stampfer MJ, Colditz GA, Willett WC, Krolewski As, Rosner B, Hennekens CH, Speizer FE. Physical activity and incidence of non-insulin-dependent mellitus in women. Lancet, 338:774-778, 1991.

Mau M, Grandinetti A, Arakaki RF, Ladao NKB, Chang HK, Curb DJ. Diabetes Mellitus and Insulin Resistance Syndrome in Native Hawaiians. American Diabetes Association, 639, 1995 (Abstract).

McCullagh P. Regression models for ordinal data. Journal of the Royal Statistical Society, 42:109-142, 1980.

McKeigue PM, Pierpoint T, Ferrie JE, Marmot MG. Relationship of glucose intolerance and hyperinsulinaemia to body fat pattern in south Asians and Europeans. Diabetologia, 35:785-791, 1992.

McPhillips JB, Barrett-Connor E, Wingard DL. Cardiovascular disease risk factors prior to the diagnosis of impaired glucose tolerance and non-insulin-dependent diabetes mellitus in a community of older adults. American Journal of Epidemiology, 131:443-453, 1990.

Megill DM, Hoy WE, Woodruff SD. Rates and causes of end-stage renal disease in Navajo Indians, 1971-1985. West J Med, 149:178-182. 1988.

Meigs J, Singer D, Nathan DM, et al. Metabolic abnormalities associated with glucose intolerance extend across the spectrum of prevalent glucose intolerance in 3297 Framingham offspring study subjects. Diabetes, 44: in press, 1995.

Metzger BE, Bybee DE, Freinkel N, Phelps RL, Radvany RM, Vaisrub N. Gestational Diabetes Mellitus. Correlations between the phenotypic and genotypic characteristics of the mother and abnormal glucose tolerance during the first year postpartum. Diabetes, 34:115, 1985.

Miller RG. Simultaneous Statistical Inference. New York, Springer-Verlag, 1981.

Miller WR, Benefield G, Tonigan JS. Enhancing motivation for change in problem drinking: a controlled comparison of two therapist styles. Journal of Consulting and Clinical Psychology, 61:455-461, 1993.

Mitchell BD, Valdez R, Hazuda HP, Haffner SM, Monterrosa A, and Stern MP. Differences in the prevalence of diabetes an impaired glucose tolerance according to maternal or paternal history of diabetes. Diabetes Care, 16:1262-1267, 1993.

Modan M, Halkin H, Almog S, Lusky A, Eshkol A, Shefi M, Shitrit A, Fuchs Z. Hyperinsulinemia. A link between hypertension obesity and glucose intolerance. Journal of Clinical Investigation, 75: 809-817, 1985.

Modan M, Karasik A, Halkin H, Fuchs Z, Lusky A, Shitrit A., Modan B. Effect of past and concurrent body mass index on prevalence of glucose intolerance and type 2 (non-insulin-dependent) diabetes and on insulin response. The Israel study of glucose intolerance, obesity and hypertension. Diabetologia, 29: 82-89, 1986.

Monterrosa A, Haffner SM, Stern MP, Hazuda HP. Sex difference in lifestyle factors predictive of diabetes in Mexican-Americans. Diabetes Care, 18:448-456, 1995.

Mykkanen L, Haffner SM, Ronnemaa T, Bergman R, Leino A, Laakso M. Is there a sex difference in the association of plasma insulin level and insulin sensitivity with serum lipids and lipoproteins. Metabolism, 43:523-528, 1994.

Nathan DM. Long-term complications of diabetes mellitus. New England Journal of Medicine, 328:1676-85, 1993.

National Diabetes Data Group. Classification and diagnosis of diabetes mellitus and other categories of glucose intolerance. Diabetes, 28:1039-1057, 1979.

National Institute of Diabetes and Digestive and Kidney Diseases. Diabetes Statistics. Bethesda, National Institutes of Health, 1995 (NIH Publication No. 96-3926).

Nelson RG, Newman JM, Knowler WC, Sievers ML, Kunzelman CL, Pettitt DJ, Moffett CD, Teutsch SM, Bennett PH. Incidence of end-stage renal disease in type 2 (non-insulin-dependent) diabetes mellitus in Pima Indians. Diabetologia, 31:730-736, 1988

Nolan JJ, Ludvik B, Beerdsen P, Joyce M, Olefsky J. Improvement in glucose tolerance and insulin resistance in obese subjects treated with troglitazone. New England Journal of Medicine, 331:1188-93, 1994.

O'Rahilly S, Turner RC, Matthews DR, Phil D. Impaired pulsatile secretion of insulin in relatives of patients with non-insulin-dependent diabetes. New England Journal of Medicine, 318:1225-30,1988.

O'Sullivan JB, Mahan CM. Prospective study of 352 young patients with chemical diabetes. New England Journal of Medicine, 278:1038-1041, 1968.

O'Brien PC, Fleming TR. A multiple testing procedure for clinical trials. Biometrics, 35:549-556, 1979.

O'Sullivan JB, Mahan CM. Blood sugar levels, glycosuria, and body weight related to development of diabetes mellitus. Journal of the American Medical Association, 194:117-122, 1965.

O'Sullivan JB. Diabetes Mellitus After GDM. Diabetes, 40(Suppl 2):131-135, 1991.

Ohlson L-O, Larsson B, Svardsudd K, Welin L, Eriksson H, Wilhelmsen L, Fjorntorp P, Tibblin G. The influence of body fat distribution on the incidence of diabetes mellitus: 13.5 years of follow-up of the participants in the study of men born in 1913. Diabetes, 34:1055-1058, 1985.

Pan X, Guangwei L, Yinghua H, Jixing W, Bennett PH, Howard BV. Effect of dietary and/or exercise interventions on incidence of diabetes in 530 subjects with IGT - The Da-Qing IGT and diabetes study. IDF Federation Congress Program, 489, 1994 (Abstract).

Parke Davis. Troglitazone Investigator Manual

Peduzzi P, Wittes J, Detre K, Holford T. Analysis as-randomized and the problem of non-adherence: an example from the Veterans Affairs Randomized Trial of Coronary Artery Bypass Surgery. Statistics in Medicine, 12:1185-1195, 1993.

Perry IJ, Wannamethee SG, Walker MK, Thomson AG, Whincup PH, Shaper AG. Prospective study of risk factors for the development of non-insane dependent diabetes in middle aged British men. British Medical Journal, 310: 560-564, 1995.

Rubin RJ, Altman WM, Mendelson DN. Health care expenditures for people with diabetes mellitus, 1992. Journal of Clinical Endocrinology and Metabolism, 78:809A-809F, 1994.

Rudnichi A, Fontbonne A, Safar M, Bard JM, Vague P, Juhan-Vague I, Eschwege E, and the BIGPRO Study Group. The effect of metformin on the metabolic anomalies associated with android type body fat distribution. Results of the BIGPRO Trial. Diabetes, 43(Suppl 1):150A, 1994.

Saad MF, Knowler WC, Pettitt DJ, Nelson RG, and others. A two-step model for development of non-insulin-dependent diabetes. American Journal of Medicine, 90:229-235, 1991.

Saad MF, Knowler WC, Pettitt DJ, Nelson RG, Mott SM, and Bennett PH. The natural history of impaired glucose tolerance in the Pima Indians. The New England Journal of Medicine, 319:1500-1506, 1988.

Salomaa VV, Jauhiainen M, Pietinen P, Korhonen HJ, Kartovaara L, Vartiainen E, Tuomilehto J. Five-year trend in serum HDL-lipoprotein cholesterol in the Finnish population aged 25-64 years. A suggestion of an increase. Atherosclerosis, 86: 39-48, 1991.

Sartor G, Schersten B, Carlstrom S, Melander A, Norden A, Persson G. Ten-year follow-up of subjects with impaired glucose tolerance: prevention of diabetes by tolbutamide and diet regulation. Diabetes, 29:41-44, 1980.

Savage PJ, Bennion LJ, Nagulesparan M, Mott DM, Roth J, Unger RH, Bennett PH. Diet-induced improvement of abnormalities in insulin and glucogon secretion and in insulin receptor binding in diabetes mellitus. Journal of Clinical Endocrinology Metabolism, 48:999-1007, 1979.

Saxman KA, Barrett-Connor EL, Morton DJ. Thiazide-associated metabolic abnormalities and estrogen replacement therapy: an epidemiological analysis of postmenopausal women in Rancho Bernardo, California. Journal of Clinical Endocrinology and Metabolism, 78:1059-1063, 1994.

Schneider, S. H.; Khachadurian, A. K.; Amorosa, L. F.; Clemow, L.; Ruderman, N. B. Ten-year experience with an exercise-based outpatient life-style modification program in the treatment of diabetes mellitus. Diabetes Care, 15:1800-10, 1992.

Second report of the Expert Panel on Detection, Evaluation, and Treatment of High Blood Cholesterol in Adults (Adult Treatment Panel II). Detection, evaluation, and treatment of high blood cholesterol in adults/. [Bethesda, MD] :National Cholesterol Education Program, National Institutes of Health, National Heart, Lung, and Blood Institute, 1993]. (NIH publication; no. 93-3095/G).

Sicree RA, Zimmet PZ, King HOM, Coventry JS. Plasma insulin response among Nauruans: prediction of deterioration in glucose tolerance over 6 years. Diabetes, 36:179-186, 1987.

Skarfors ET, Lithell HO, Selinus I, Aberg H. Do antihypertensive drugs precipitate diabetes in predisposed men? BMJ, 298: 1147-1152, 1989.

Stamler R, Stamler J, Grimm R, Gosch FC, Elmer P, Dyer A, Berman R, Fishman J, Van Heel J, Cininelli J, McDonald A. Nutritional therapy for high blood pressure: final report of a four-year randomized controlled trial - The Hypertension Control Program. Journal of the American Medical Association, 257:1484-1501, 1987.

Stamler R, Stamler J, Schoenberger JA, Shekelle RB, Collette P, Shekelle S, Dyer A, Garside D, Wannamaker J. Relationship of glucose tolerance to prevalence of ECG abnormalities and to 5-year mortality from cardiovascular disease: findings of the Chicago Heart Association Detection Project in Industry. Journal of Chronic Diseases, 32: 817-828, 1979.

Taylor R, Ram P, Zimmet P, Raper LR, Ringrose H. Physical activity and prevalence of diabetes in Melanesian and Indian men in Fiji. Diabetologia, 27: 578-582, 1984.

Trials of Hypertension Prevention Collaborative Research Group. The effects of nonpharmacologic interventions on blood pressure of persons with high normal levels. Journal of American Medical Association, 267:1213-1220, 1992.

UKPDS Group. United Kingdom Prospective Diabetes Study 13. Relative efficacy of randomly allocated diet, sulphonylurea, insulin, or metformin in patients with newly diagnosed non-insulin dependent diabetes followed for three years. British Medical Journal, 310:83-88, 1995.

Valiquett T, Balagtus B, Whitcomb R. Troglitazone Dose-Response Study in Patients with NIDDM. American Diabetes Association, 406, 1995 (Abstract).

Warram JH, Martin BC, Krolewski AS, Soeldner JS, Kahn CR. Slow glucose removal rate and hyperinsulinemia precede the development of type II diabetes in the offspring of diabetic parents. Annals of Internal Medicine, 113: 909-15, 1990.

Wei LJ, Lachin JM. Two-sample asymptotically distribution-free tests for incomplete multivariate observations. Journal of the American Statistical Association, 79:653-661, 1984.

Wei LJ, Lachin JM. Properties of Urn Randomization in Clinical Trials. Controlled Clinical Trials, 9:345-364, 1988.

Wei LJ. The accelerated failure time model: a useful alternative to the Cox regression model in survival analysis. Statistics in Medicine, 11:1871-1879, 1992.
West KM, Kalbfleisch JM. Influence of nutritional factors on prevalence of diabetes. Diabetes, 20: 99-108, 1971.
West KM. Epidemiology of Diabetes and Its Vascular Lesions. New York: Elsevier, 1978.
Wingard DL, Barrett-Connor EL, Scheidt-Nave C, McPhillips JB. Prevalence of cardiovascular and renal complications in older adults with normal or impaired glucose tolerance or NIDDM. Diabetes Care, 16:1022-1025, 1993.
World Health Organization. WHO Expert Committee on Diabetes Mellitus, Second Report. Geneva, World Health Organization, 1980 (Tech. Rep. Ser. No. 646).
Yudkin JS, Alberti KGMM, McLarty DG, Swai GPM. Impaired glucose tolerance. British Medical Journal, 301:397-402, 1990.
Yudkin JS. Hypertension and non-insulin dependent diabetes. BMJ, 303: 730-732, 1991.
Zimmet P, Type II (non-insulin dependent) diabetes--an epidemiological overview. Diabetologia, 22:399-411, 1982.

14.1 Section 14.1 Bibliography for Background to the Washout Study

Diabetes Prevention Program Research Group. The Diabetes Prevention Program: Design and methods for a clinical trial in the prevention of type 2 diabetes mellitus. Diabetes Care 1999, 22:623-34.
Bailey CJ. Biguanides and NIDDM. Diabetes Care 1992;15:755-72.
Strumvoll M, Nurgham N, Perriello G, et al. Metabolic effects of metformin in non-insulin-dependent diabetes mellitus. N Engl J Med 1995;333:550-54.
Bailey CJ, Turner RC. Metformin. N Engl J Med 1996;334:574-79.

15. Consent Forms......15-1
15.1 Information and Consent for Screening Step 1......15-1
15.2 Information and Consent for Screening Step 2......15-4
15.3 Information and Consent for Screening Step 3......15-7
15.4 Information and Consent for the Treatment Phase......15-14
15.5 Information and Consent for Banking and Use of the Blood and Genetic Material......15-20
15.6 Information and Amendment for the completion of the Masked Treatment Phase......15-26
15.7 Information and Consent for a "Washout" Study of Metformin and Placebo......15-30
15.8 Consent Forms for Bridge Period......15-34

15. Consent Forms

15.1 Information and Consent for Screening Step 1

NAME OF INSTITUTION

INFORMATION AND CONSENT FOR SCREENING STEP 1
DIABETES PREVENTION PROGRAM (DPP)

INVESTIGATORS

Name of Principal Investigator
Title, Affiliation, Phone Number

Name of Co-Investigator(s)
Title, Affiliation, Phone Number

Name of Program Coordinator
Title, Affiliation, Phone Number

Name of Recruitment Coordinator
Title, Affiliation, Phone Number

24-Hour Emergency Telephone Number
(List phone number(s) here)

INVESTIGATOR'S STATEMENT:

PURPOSE

This research study is called the Diabetes Prevention Program (DPP). This study will test several ways to prevent the common form of diabetes (Type 2 Diabetes, also called Non-Insulin-Dependent Diabetes Mellitus or NIDDM). The purpose of this part of screening for DPP is to find out more about your risk for getting Type 2 Diabetes by doing a blood test. Depending upon the results of this test, you will be offered further tests to find out more about your blood level of glucose (sugar).

BENEFITS

This blood test will show whether you might have diabetes. You may choose to not find this out by not taking part in this test. If the test suggests that you might have diabetes, we will inform you and you will be referred to a doctor of your choice for treatment. If you do not have a doctor, we will help you to find a doctor for treatment.

PROCEDURES

You will be asked questions about your health. You will have your height, weight, and blood pressure measured (in some persons this might be postponed to the next visit). A small drop of blood will be taken from your finger in order to measure the amount of glucose (sugar) in your blood. This visit will take up to 30 minutes. If the results from this visit show that it might be all right for you to take part in the study, you will be invited back for more tests.

RISKS AND DISCOMFORTS

There is brief pain in the fingertip from the fingerstick. Taking blood from your arm might cause temporary discomfort and possible bruising or redness of the skin. You might feel uncomfortable about some of the questions asked of you. You may choose not to answer any questions.

OTHER INFORMATION

(Institutional language as suggested by your IRB might be substituted for the following sections.)

Information that we get from you will be kept confidential to the extent allowed by law. Taking part in this study is completely voluntary. You are free to take back your consent and stop taking part at any time. You may ask any questions about the study at any time. All study procedures will be free of charge.

If you are hurt as a direct result of the study, medical treatment will be done at no cost within the limits of our compensation plan. Beyond this, treatment will be at your expense or that of your insurance carrier.

If you have any more questions about your rights as a research subject, you may call (name of IRB contact) at (IRB phone number.)

____
Investigator’s Signature Date

PARTICIPANT'S STATEMENT:

The study described above has been explained to me. I understand that I am consenting to participate in this part of the study. If I have any questions about the study, I know that I can contact any of the persons listed on the first page.

____

Participant's Signature Date

cc: Investigator

15.2 Information and Consent for Screening Step 2

NAME OF INSTITUTION

INFORMATION AND CONSENT FOR SCREENING STEP 2 DIABETES PREVENTION PROGRAM (DPP)

INVESTIGATORS

Name of Principal Investigator
Title, Affiliation, Phone Number

Name of Co-Investigator(s)
Title, Affiliation, Phone Number

Name of Program Coordinator
Title, Affiliation, Phone Number

Name of Recruitment Coordinator
Title, Affiliation, Phone Number

24-Hour Emergency Telephone Number
(List phone number(s) here)

INVESTIGATOR'S STATEMENT:

PURPOSE

This research study is called the Diabetes Prevention Program (DPP). This study will test several ways to prevent the common form of diabetes (Type 2 Diabetes, also called Non-Insulin-Dependent Diabetes Mellitus or NIDDM). The purpose of this part of screening for DPP is to find out more about your risk for getting Type 2 Diabetes by doing an oral glucose tolerance test. If this test shows you have impaired glucose tolerance (also called IGT) and other conditions are met, you will be offered the chance to join DPP.

BENEFITS

By doing the oral glucose tolerance test, early detection of diabetes is possible. From this test, we will be able to tell whether you have diabetes, IGT, or normal glucose tolerance. You may choose not to find this out by not taking part in this test. If the test suggests that you might have diabetes, you will be referred to a doctor of your choice for treatment. If you do not have a doctor, we will help you to find a doctor for treatment.

PROCEDURES

You will be asked to not eat or drink anything, except water, for 12 hours before your appointment.

1. Height, weight, and blood pressure will be checked (if not done previously). This will take about 10 minutes.

2. A blood sample, about 1 teaspoon, will be taken from your arm and the glucose level measured right away. You will also be asked to give a urine sample. This will take about 15 minutes.

3. Depending upon the level of glucose in this test, an oral glucose tolerance test will be done. Another blood sample, about 2 tablespoons, will be taken from your arm (this might be postponed until a later visit). You will then be asked to drink a glassful of flavored sugar water over 5 to 10 minutes. Another blood sample, about 1 tablespoon, will be taken from your arm at 30 and again at 120 minutes later. This test will take up to 2 and ½ hours.

RISKS AND DISCOMFORTS

The risks of drawing blood include temporary discomfort from the needle stick, and possible bruising or redness of the skin. About one out of ten people have mild nausea or an upset stomach with the glucose (sugar) drink that is given during the oral glucose tolerance test. You might feel uncomfortable about some of the questions asked of you. You may choose not to answer any questions.

OTHER INFORMATION

(Institutional language as suggested by your IRB may be substituted for the following sections.)

Information that we get from you will be kept confidential to the extent allowed under law. You are free to take back your consent and stop taking part at any time. You may ask any questions about the study at any time. All study procedures will be free of charge.

If you are hurt as a direct result of the study, medical treatment will be done at no cost within the limits of our compensation plan. Beyond this, treatment will be at your expense or that of your insurance carrier.

If you have any more questions about your rights as a research subject, you may call (name of IRB contact) at (IRB phone number.)

____
Investigator's Signature   Date

PARTICIPANT'S STATEMENT:

The study described above has been explained to me. I understand that I am consenting to participate in this part of the study. If I have any questions about the study, I know that I can contact any of the persons listed on the first page.

____

Participant's Signature   Date

cc: Investigator

15.3 Information and Consent for Screening Step 3

NAME OF INSTITUTION<br><br>INFORMATION AND CONSENT FOR SCREENING STEP 3<br>DIABETES PREVENTION PROGRAM (DPP)

INVESTIGATORS

Name of Principal Investigator
Title, Affiliation, Phone Number

Name of Co-Investigator(s)
Title, Affiliation, Phone Number

Name of Program Coordinator
Title, Affiliation, Phone Number

Name of Recruitment Coordinator
Title, Affiliation, Phone Number

**24-Hour Emergency Telephone Number**<br>(List phone number(s) here)

INVESTIGATOR'S STATEMENT:

PURPOSE AND BACKGROUND

This research study is called the Diabetes Prevention Program (DPP). This study will test several ways to prevent the common form of diabetes (Type 2 Diabetes, also called Non-Insulin-Dependent Diabetes Mellitus or NIDDM).

Diabetes is a disease in which there is too much glucose (sugar) in the blood. Diabetes causes damage to blood vessels, heart, kidneys, eyes, and nerves. Diabetes affects at least 16 million Americans. Ninety to ninety-five percent of those affected have Type 2 Diabetes. Those who have a condition called Impaired Glucose Tolerance (IGT) have a much higher risk of getting Type 2 Diabetes.

Eating too much or the wrong types of food and lack of exercise might lead to Type 2 Diabetes, perhaps by causing weight gain. Healthy eating habits, weight loss, and exercise such as walking might prevent Type 2 Diabetes. Some medicines might also prevent Type 2 Diabetes.

With this in mind, the purpose of DPP is to find out whether healthy eating habits and exercise, or whether medicine will prevent Type 2 Diabetes in persons who have IGT, and the safety of these treatments. We have found that you have IGT.

BENEFITS

Volunteers in this study get a complete physical exam. We will tell you if we find any health problems and if you permit it, we will tell your doctor. Your health will be closely watched during the study, and problems such as diabetes might be found and treated sooner than if you were not in the study. This might improve your health. Your part in this research will also help us learn whether it is possible to prevent or delay Type 2 Diabetes. You also have the choice of not joining this study.

PROCEDURES

You will be asked to have a physical exam and an electrocardiogram (ECG) to learn more about your health. This will take about 1 hour.

This will be followed by a "run-in" period during which you will complete some tasks. At the beginning of this run-in period, you will be taught how to fill out a daily diary. You will also be taught how to take placebo (inactive medication) pills according to a set schedule. DPP will ask participants to attend regular meetings, keep records like these, or take medications daily for a period of 3 to 6 years. This is hard for many people. We do not want to encourage you to join DPP if it does not make sense for you. The purpose of this "run-in" is to give you a chance to see how this will work for you.

If you complete these tasks satisfactorily, you will be asked again whether you would like to join the study. If you do, you will be given another Informed Consent in which you are asked to agree to be placed into one of three treatment groups. You will not be able to choose your group. You will be put into a group by chance (like flipping a coin).

A baseline blood test will be done. You will be asked not to eat or drink anything, except water, for 12 hr before this test. A blood sample (about 2 tablespoons) will be taken from your arm.

Once you have completed these tests, you will be assigned to one of the following three treatment groups:

1. Group A: An intensive program of exercise and diet with goals of walking for 2 ½ hours each week and using healthy eating habits in order to lose about 7% of your weight. You will receive help from a trained professional who will help you change your diet and exercise. You will meet with this person for 16 sessions over a 24 week period. These meetings will last about 30 minutes. After this initial period, you will meet at least once every 2 months for the rest of the study. For extra help, exercise classes will be offered 2 times a week. There will also be small group classes given each year to help you learn more about food choices and where you can talk about problems you might be having in sticking to this exercise and diet program.

2. Group B: Healthy eating and exercise habits ("standard care"), and taking an inactive medicine called placebo. "Standard care" is the type of treatment you expect from your own doctor if you are thought to be at high risk for getting diabetes, if you are overweight, or if you are physically inactive. The placebo will be given once daily and then increased to twice daily. The use of inactive medicine (placebo) is needed so that doctors can find out whether the medicines being tested are really doing good and to find out whether they are having any side effects. Although the placebo medicine is inactive,

your health will be closely watched, making it likely that problems will be found sooner than if you were not in the study. Any problems found will be told to you. You will be asked to return to the clinic at 1 month and at 3 months in order to be sure that you are doing all right on the medicine. Visits will then be every 3 months.

3. Group C: "Standard care", taking metformin, a medicine used for lowering blood sugar. The dose of metformin will start at 850 mg daily and increase to 850 mg twice a day. Like Group B, your health will be closely watched with clinic visits at 1 month, 3 months and then every 3 months.

If you are in the Intensive Diet and Exercise group (Group A) you will need a exercise stress test if you have a history of heart disease. The purpose of this test is to find out your heart's response to exercise. This is an exercise test that is often done by doctors to see whether someone has significant heart disease. In this test you will walk on a treadmill that will have the slope (steepness) and speed increased every 3 minutes. An electrocardiogram will be continuously reading the response of your heart to this exercise test. You may stop the test at any time. The doctor monitoring the test may also stop the test at any time. This test will take about 30 minutes, including preparation time.

If you are in the medicine group (Group B or C) you, your DPP clinic's staff, and your own doctor will not know whether you are taking an active medicine (metformin) or the inactive medicine (placebo). This is to be sure that those taking the inactive medicine and those taking the active medicine are treated the same. If a medical emergency occurs that makes it necessary to find out which of these you are taking, your doctor and the study clinic staff will find out.

The dose of medicine will be increased during the first few weeks. The dose of medicine might also be reduced if you get symptoms that might be side effects related to taking medicine.

Procedures to be carried out while you are in the study include the following:

1. The physical examination and electrocardiogram (ECG) done at baseline will be done every 12 months. This will take about 1 hour.

2. Blood pressure will be measured in your arm every 6 months. This will take about 5 minutes. Blood pressure will be measured in your ankle every 12 months. This will take about 15 minutes.

3. Blood flow and blood vessel wall thickness will be measured by ultrasound in your neck. This will take about 30 minutes.

4. Body measurements: Your height, weight, and waist and hip size will be measured. This test will take 10 minutes and will be done every 12 months. In addition, the height of your abdomen while you are lying down will be measured at baseline, at 1 year, and at the end of the study. The thickness of your skin will be measured at 5 places--upper arm, back, abdomen, and upper and lower leg--at baseline, at 1 year, and at the end of the study. These tests will take 20 minutes.

5. There are several questionnaires that you will be asked to fill out every 12 months. These have to do with your physical activity, your diet, and your reactions (feelings) about the study. These will take about 45 minutes to complete.

6. A blood sample will be taken from your arm (about 2 tablespoons). This will be done every 6 months. You will be asked not to eat or drink anything, except water for 12 hours before your appointment. A repeat blood sample (about 1 tablespoon) might be taken in some persons.

7. Oral glucose test: You had this test during screening for the study. This test will take about 2 and ½ hours. It will be done every 12 months. You will be asked to not eat or drink anything, except water, for 12 hours before your appointment. A blood sample, about 1 tablespoon, will be taken from your arm. You will then be asked to drink a glassful of flavored sugar water over 5 minutes. Another blood sample (about 1 tablespoon) will be taken from your arm at 30 and again at 120 minutes later.

8. A urine sample will be collected at baseline and at the end of the study.

9. DNA: A blood sample (about 2 tablespoons) will be taken from your arm in order to get DNA from the blood cells. This will be used to look for genes that might be related to Type 2 Diabetes and related conditions.

10. If you are in a medication group a blood sample (about 1 tablespoon) will be taken from your arm at month 3, month 6 and then every 6 months.

Taking part in this study will require our knowing about any other medicines that you might be using or might wish to use. Some medicines are not preferred, but many others are all right. Should you need to start taking medicine, the study doctors will work closely with your own doctor to tell you which you may take. If you do not have your own doctor, the study doctors will direct you to medical care.

If you are a woman and there is a chance that you could become pregnant, you will be asked whether you are willing to use medically effective birth control methods for the duration of the study. If you decide to become pregnant during the study, you must notify the clinic staff immediately. If there is a chance that you could be pregnant, a blood pregnancy test will be done.

The study will last until 2002. Thus you will take part in the study for 3 to 6 years, depending upon when you entered the study.

RISKS, STRESS, AND DISCOMFORT

The screening part of the study will let us know who will be able to safely be a part of the study. For those who go on to take part in the treatment part of this study, these are the possible risks:

1. Exercise stress test (Group A): There is a small risk of sudden death, heart attack, and muscle or joint injury with this test. Sudden death has occurred in less than 2 out of 10,000 tests. Heart attack has occurred in less than 1 out of 1,000 tests. The risk of this test is lowered by taking a careful medical history before the test, doing the screening electrocardiogram, and doing the test under medical supervision.

2. Oral glucose and blood tests: The risks of drawing blood include temporary discomfort from the needle stick, and possible bruising or redness of the skin. You might get nausea or an upset stomach with the glucose (sugar) drink that is given during the oral glucose test.

3. Exercise: Fatigue, muscle soreness, and injury such as sprained ankles or pulled muscles might happen. You will be tested to make certain that there are no health reasons why you should not exercise. Risks are reduced by proper warm-up and cool-down periods and careful planning of the exercise for each person. Exercise will be designed for you and tracked by experienced staff while you are being taught.

4. Medicine:

Placebo: Some persons will get inactive medication or placebo. If you receive placebo, you will not get any benefit of the active medicine, metformin, if it does have a good effect. You, the clinic staff, and your own doctor will not know whether you are getting placebo or metformin.

Metformin: This medicine has been used for many years to treat patients with diabetes. Side effects include: loss of appetite, upset stomach, vomiting, stomach pain, diarrhea, bloating or gas, or a funny taste (like metal). These are usually mild and get less with continued use of the medicine. Mild side effects might happen in one out of five persons. Only one or two persons out of fifty are expected to have to stop metformin because of side effects. Anemia or liver test abnormalities might also happen in some persons. Very few persons (3 in 100,000 and usually persons with poor kidney function or with liver disease) have serious problems (a condition known as lactic acidosis) with metformin that might result in death. Metformin should not be used by persons with poor kidney function or liver disease, or women planning to become pregnant. We are therefore not allowing persons with poor kidney function or liver disease, or women who are planning to become pregnant, to take part in this study. Tests will be done every 6 months to check on these. It is always possible that you could have an unexpected serious reaction to metformin or any other medicine.

If you are a woman and there is a chance that you could become pregnant, you will be asked whether you are willing to use acceptable birth control methods for the duration of the study. If you decide to become pregnant during the study, you must notify the clinic staff immediately. If there is a chance that you might be pregnant, a blood pregnancy test will be done. A blood pregnancy test will also be done before some of the procedures.

Metformin has not resulted in any increased risk during pregnancy. However, metformin has not been approved for use in pregnancy. It is important that you tell us if you think that you might be pregnant or decide to become pregnant. Study medicines will be stopped if you become pregnant and restarted after the pregnancy and nursing.

There is no risk or discomfort with the ultrasound blood vessel test.

You may feel uncomfortable about some of the questions asked of you. You might choose not to answer any questions.

OTHER INFORMATION

Some of the test results will not be made known to you or the clinic staff or your own doctor. This is known as "masking". Blood tests will be masked whereas weight and blood pressure will not be masked. Some times knowing the results of the tests might make it harder to treat all of the study groups equally. If any of the test results are abnormal, you will be notified.

Some of the blood taken will be used to get DNA. This will be used to try to find genes that are important in Type 2 Diabetes and related conditions. The DNA material will not be linked to your name.

(Institutional language as suggested by your IRB should be substituted for the following sections.)

Participation in this study is completely voluntary. You are free to take back your consent and stop taking part in this study at any time. You may ask any questions about the study at any time. Your current or future care will not be affected by your stopping the study. All study procedures will be free of charge. You will receive money ($150 a year) and gifts for your time and effort.

Information that we get from you will be kept confidential to the extent allowable under law.

[Each center should incorporate a statement to address medical liability.]

If a test result shows that you should get medical care, you will be referred to a doctor of your choice. If you do not have a doctor, we will help you to see a doctor for treatment.

This study can be stopped or modified at any time.

If you have any questions about your rights as a research subject, you may call (IRB contact name) at (IRB phone number.)

____

Investigator's Signature Date

PARTICIPANT'S STATEMENT:

The study described above has been explained to me. I understand that I am consenting to take part in this part of the screening phase of this study. This will complete the screening phase of this study. If I have any questions, I know that I can contact one of the investigators listed on the first page.

____

Participant's Signature Date

cc: Investigator

15.4 Information and Consent for the Treatment Phase

NAME OF INSTITUTION

INFORMATION AND CONSENT FOR THE TREATMENT PHASE DIABETES PREVENTION PROGRAM (DPP)

INVESTIGATORS

Name of Principal Investigator
Title, Affiliation, Phone Number

Name of Co-Investigator(s)
Title, Affiliation, Phone Number

Name of Program Coordinator
Title, Affiliation, Phone Number

Name of Recruitment Coordinator
Title, Affiliation, Phone Number

24-Hour Emergency Telephone Number
(List phone number(s) here)

INVESTIGATOR'S STATEMENT:

PURPOSE AND BACKGROUND

This research study is called the Diabetes Prevention Program (DPP). You have been given the details concerning the Purpose and Background of this study in the previous Consent Form. You are eligible to take part in the treatment phase of DPP because we have found that you have Impaired Glucose Tolerance (IGT) and have passed the screening phase of the study. **This consent form concerns your taking part in the treatment phase of DPP and being assigned to one of the three treatment groups.**

This study will test several ways to prevent the common form of diabetes (Type 2 Diabetes, also called Non-Insulin-Dependent Diabetes Mellitus or NIDDM). With this in mind, the purpose of DPP is to find out whether healthy eating habits and exercise, or whether medicine will prevent Type 2 Diabetes, and the safety of these treatments. You will take part in this phase of the study for 3 to 6 years.

BENEFITS

You have already received an extensive medical examination. We will tell you if we find any problems and if you permit it, we will tell you doctor. Your health will be closely watched during this study, and problems such as diabetes might be found and treated sooner than if you were not in the study. This might improve your health.

Screening tests have been done that show you may take part in this study. These screening tests have shown that your future risk of getting diabetes is very high because you have Impaired Glucose Tolerance (IGT). Your part in this research will help us learn how to prevent or delay Type 2 Diabetes. Your taking part in this study might delay or prevent your getting diabetes. You also have the choice of not joining this study.

PROCEDURES

Now that you are ready to begin the treatments, you will be randomly placed in one of three groups. You will not be able to choose your group. You will be put into a group by chance (like flipping a coin). The study will last until 2002. Thus you will take part in the study for 3 to 6 years, depending upon when you entered the study.

1. Group A: An intensive program of exercise and diet with goals of walking for 2 ½ hours each week and using healthy eating habits in order to lose about 7% of your weight. You will receive help from a trained professional who will help you change your diet and exercise. You will meet with this person for 16 sessions over a 24 week period. These meetings will last about 30 minutes. After this initial period, you will meet at least once every 2 months for the rest of the study. For extra help, exercise classes will be offered 2 times a week. There will also be small group classes given each year to help you learn more about food choices and where you can talk about problems you might be having in sticking to this exercise and diet program.

2. Group B: Healthy eating and exercise habits ("standard care"), and taking an inactive medicine called placebo. "Standard care" is the type of treatment you expect from your own doctor if you are thought to be at high risk for getting diabetes, if you are overweight, or if you are physically inactive. The placebo will be given once daily and then increased to twice daily. The use of inactive medicine (placebo) is needed so that doctors can find out whether the medicine being tested is really doing good and to find out whether it is having any side effects. Although the placebo medicine is inactive, your health will be closely watched, making it likely that problems will be found sooner than if you were not in the study. Any problems found will be told to you. You will be asked to return at 1 month and at 3 months in order to be sure that you are doing all right on the medicine. Visits will then be every 3 months.

3. Group C: "Standard care", taking metformin, a medicine used for lowering blood sugar. The dose of metformin will start at 850 mg daily and increase to 850 mg twice a day. Like Group B, your health will be closely watched with clinic visits at 1 month, 3 months, and then every 3 months.

If you are in the Intensive Diet and Exercise group (Group A) you will need a exercise stress test if you have a history of heart disease. The purpose of this test is to find out your heart's response to exercise. This is an exercise test that is often done by doctors to see whether someone has significant heart disease. In this test you will walk on a treadmill that will have the

slope (steepness) and speed increased every 3 minutes. An electrocardiogram will be continuously reading the response of your heart to this exercise test. You may stop the test at any time. The doctor monitoring the test may also stop the test at any time. This test will take about 30 minutes, including preparation time.

If you are in the medicine group (Group B or C) you, your DPP clinic's staff, and your own doctor will not know whether you are taking an active medicine (metformin) or the inactive medicine (placebo). This is to be sure that those taking the inactive medicine and those taking the active medicine are treated the same. If a medical emergency occurs that makes it necessary to find out which of these you are taking, your doctor and the study clinic staff will find out.

The dose of medicine will be increased during the first few weeks. The dose of medicine might also be reduced if you get symptoms that might be side effects related to taking medicine.

Procedures to be carried out while you are in the study include the following:

1. The physical examination and electrocardiogram (ECG) done at baseline will be done every 12 months. This will take about 1 hour.

2. Blood pressure will be measured in your arm every 6 months. This will take about 5 minutes. Blood pressure will be measured in your ankle every 12 months. This will take about 15 minutes.

3. Blood flow and blood vessel wall thickness will be measured by ultrasound in your neck. This will take about 30 minutes.

4. Body measurements: Your height, weight, and waist and hip size will be measured. This test will take 10 minutes and will be done every 12 months. In addition, the height of your abdomen while you are lying down will be measured at baseline, at 1 year, and at the end of the study. The thickness of your skin will be measured at 5 places--upper arm, back, abdomen, and upper and lower leg--at baseline, at 1 year, and at the end of the study. These tests will take 20 minutes.

5. There are several questionnaires that you will be asked to fill out every 12 months. These have to do with your physical activity, your diet, and your reactions (feelings) about the study. These will take about 45 minutes to complete.

6. A blood sample will be taken from your arm (about 2 tablespoons). This will be done every 6 months. You will be asked not to eat or drink anything, except water for 12 hours before your appointment. A repeat blood sample (about 1 tablespoon) might be taken in some persons.

7. Oral glucose test: You had this test during screening for the study. This test will take about 2 and ½ hours. It will be done every 12 months. You will be asked to not eat or drink anything, except water, for 12 hours before your appointment. A blood sample, about 1 tablespoon, will be taken from your arm. You will then be asked to drink a glassful of flavored sugar water over 5 minutes. Another blood sample (about 1 tablespoon) will be taken from your arm at 30 and again at 120 minutes later.

8. A urine sample will be collected at baseline and at the end of the study.

9. DNA: A blood sample (about 2 tablespoons) will be taken from your arm in order to get DNA from the blood cells. This will be used to look for genes that might be related to Type 2 Diabetes and related conditions.

10. If you are in a medication group a blood sample (about 1 tablespoon) will be taken from your arm at month 3, month 6 and then every 6 months.

Taking part in this study will require our knowing about any other medicines that you might be using or might wish to use. Some medicines are not preferred, but many others are all right. Should you need to start taking medicine, the study doctors will work closely with your own doctor to tell you which you may take. If you do not have your own doctor, the study doctors will direct you to medical care.

If you are a woman and there is a chance that you could become pregnant, you will be asked whether you are willing to use medically effective birth control methods for the duration of the study. If you decide to become pregnant during the study, you must notify the clinic staff immediately. If there is a chance that you could be pregnant, a blood pregnancy test will be done.

RISKS, STRESS, AND DISCOMFORT

There are several possible risks of the study that were described to you in detail in the previous Consent Form.

As explained in the previous Consent Form, persons with poor kidney function or liver disease should not take part in this study. Periodic tests will also be done to check on these conditions.

If you are a woman and there is a chance that you could become pregnant, you will be asked whether you are willing to use medically effective birth control methods for the duration of the study. If you decide to become pregnant during the study, you must notify the clinic staff immediately. If there is a chance that you are pregnant, a blood pregnancy test will be done.

Metformin has not been approved for use in pregnancy. Metformin has not resulted in any increased risk during pregnancy. Some medicines might, however, cause birth defects to an unborn baby. It is important that you tell us if you suspect at all that you might be pregnant. Study medicines will be stopped if you get pregnant.

It is always possible that you could have an unexpected serious reaction to metformin.

OTHER INFORMATION

Some of the test results will not be made known to you or the clinic staff or your own doctor. This is known as “masking”. Blood tests will be masked whereas weight and blood pressure will not be masked. Some times knowing the results of the tests might make it harder to treat all of the study groups equally. If any of the test results are abnormal you will be notified.

Some of the blood taken will be used to get DNA. This will be used to try to find genes that are important in Type 2 Diabetes and related conditions. The DNA material will not be linked to your name.

(Institutional language as suggested by your IRB should be substituted for the following sections.)

Participation in this study is completely voluntary. You are free to take back your consent and stop taking part in this study at any time. You may ask any questions about the study at any time. Your current or future care will not be affected by your stopping the study. All study procedures will be free of charge. You will receive money ($150 a year) and gifts for your time and effort.

Information that we get from you will be kept confidential to the extent allowed by law.

We will ask you to give us personal information such as address, phone numbers, and social security number, to help us reach you if we lose touch.

[Each center should incorporate a statement to address medical liability.]

If a test result shows that you should get medical care, you will be referred to your doctor. If you do not have a doctor, we will help you to see a doctor for medical care.

This study can be stopped or modified at any time.

If you have any questions about your rights as a research subject, you may call (IRB contact name) at (IRB phone number.)

____

Investigator's Signature Date

PARTICIPANT'S STATEMENT:

The study described above has been explained to me. I understand that I am consenting to take part in the research phase of this study. If I have any questions, I know that I can contact one of the investigators listed on the first page.

____
Participant's Signature   Date

cc: Investigator
Participant

15.5 Information and Consent for Banking and Use of the Blood and Genetic Material

NAME OF INSTITUTION

INFORMATION AND CONSENT FOR BANKING AND USE OF BLOOD AND GENETIC MATERIAL (DNA) OBTAINED IN THE DIABETES PREVENTION PROGRAM (DPP)

INVESTIGATORS

Name of Principal Investigator
Title, Affiliation, Phone Number

Name of Co-Investigator(s)
Title, Affiliation, Phone Number

Name of Program Coordinator
Title, Affiliation, Phone Number

24-Hour Emergency Telephone Number
(List phone number(s) here)

INVESTIGATOR'S STATEMENT:

PURPOSE AND BACKGROUND

You have been given the details concerning the Purpose and Background of the Diabetes Prevention Program (DPP) in the DPP Treatment Phase Consent Form. **This consent form is to provide you with additional information concerning storage (banking) and use of your blood and genetic material (DNA) for future studies. You gave consent previously for blood samples to be drawn for DNA and other testing. These samples were obtained during the DPP and currently are in storage. If the blood sample you already provided for DNA during the DPP was insufficient, you will be asked to provide another sample (about 1 tablespoon) for future genetic testing.**

We are studying the development of Type 2 diabetes, and heart and blood vessel (cardiovascular) diseases in the DPP. New blood factors, and new relationships between blood factors and diabetes or heart disease, may be found in the future. Likewise, we know diabetes and heart disease run in families, but we don't know what genes are involved in the development of these diseases. Laboratory methods are available that allow tests of inherited factors called genes. By studying the DNA in your blood sample, researchers might be able to identify the gene(s) that carry the trait(s) or risk factors for problems such as Type 2 diabetes, heart disease and related conditions (high cholesterol, etc). If we do identify the specific genes, in the future we may be able to develop better diagnostic tools and treatments for these diseases.

You are being asked to participate in a blood and genetic material bank and for permission to use your blood and genetic material in future studies because you are a participant in the Diabetes Prevention Program, and are at high risk of developing diabetes or you have developed diabetes. Approximately 3800 DPP participants nationwide will be asked to participate in the blood and genetic bank and in future blood factor and genetic studies.

BENEFITS

There will be no direct benefit to you as a result of the research performed with your blood or genetic material. The tests that will be done will not be diagnostic (useful for finding medical problems). Therefore, at this time we do not plan to provide results of any blood or genetic testing to you.

The specific test(s) to be done on the blood samples has not been established at this time, and the laboratory that will perform the testing has not yet been identified. We expect that our studies of blood and genetic factors involved in diabetes, cardiovascular disease, and related conditions (such as high cholesterol) will take many years to complete. We may never, in fact, identify the specific factors or genetic material responsible for these conditions.

You will not receive any money for permission to use your blood samples and genetic material. All study procedures are free of charge.

PROCEDURES

In addition to the DPP procedures about which you have already been told and to which you have already agreed, we are asking you to review the information in this consent form concerning use of your blood and genetic material in future studies.

If the blood sample you already provided for DNA during the DPP was not sufficient, you will be asked to provide another sample. About 2 tablespoons of blood will be drawn from a vein in your arm. The blood will be stored for future blood and genetic testing. If your previous sample was adequate, you will not have more blood drawn, but you are asked to tell us about how to use these samples.

Your samples would be used only for obtaining information about blood factors and genes for diabetes, cardiovascular disease and related conditions.

RISKS, STRESS, AND DISCOMFORT

This research will not affect your medical care. Therefore, you, your family, or your doctor will not receive results of these studies, and the results will not become a part of your medical record. The study investigators will make every effort to maintain confidentiality by labeling your samples with a number rather than with your name or other personal information. However, in the unusual circumstance that your test results are unintentionally made known to a third party, or revealed to you because they are important to your medical care, this information could affect your ability to be insured and employed, and your future plans for children or your family relationships. Additionally, having information about your genes may reveal information regarding health risks to other members of your family who are now living or not yet born. If this

should occur, the study will assist you in identifying genetic counseling resources but will not pay for genetic counseling if it is necessary.

Risks associated with drawing blood from your arm include pain, bruising, lightheadedness and, on rare occasion, infection. Whenever possible, blood for the research discussed above will be drawn at the same time as samples for other DPP tests. If it is not possible, an additional needle stick will be required.

By agreeing to donate your blood to the DPP researchers, you may forfeit sharing in any financial gain, which may be obtained in the event that your donated material results in the development of a product with commercial application.

(Institutional language as suggested by your IRB should be substituted for the following sections.)

CONFIDENTIALITY

Information that we receive from you will be kept confidential to the extent allowed by law. Your blood and genetic material will be kept in storage indefinitely or until the sample is no longer viable (living). A code number identifies samples and the link between the code and your personal information is stored in a secure location at the insert your institution name. Thus, your blood and DNA will not be directly identified with your name. Your samples would be released to a DPP investigator (or other investigator authorized by the DPP) only after determination of the scientific usefulness of a proposed study.

OTHER

Participation in the blood and genetic material bank and future blood and genetic studies is entirely voluntary. You are free to take back your consent at any time and you may request that your sample be permanently removed from the blood and DNA bank. If you decide that you do not want to participate in the banking part of the DPP, you may still continue participation in the DPP. You may ask any questions about the study at any time. Your stopping the study will not affect your current or future care.

To request that your sample be permanently removed from the blood and DNA bank, contact: (insert PI name, address, phone and fax numbers).

We will ask you to give us personal information such as address, phone numbers, and social security number, to help us reach you if we lose touch.

[Each center should incorporate a statement to address medical liability.]

This study can be stopped or modified at any time.

If you have any questions about your rights as a research subject, you may call (IRB contact name) at (IRB phone number.)

____

Investigator's Signature   Date

PARTICIPANT'S STATEMENT:

The study described above has been explained to me. I understand that I am consenting to the blood and genetic storage part of DPP only as checked below. If I have any questions, I know that I can contact one of the investigators listed on the first page.

____

Participant's Signature Date

I agree to have my blood drawn (about 2 tablespoons) if determined to be necessary by the investigators:

______YES ______NO ______INITIALS

I give permission for my blood to be stored in a central bank, at a banking site to be determined, for future use by the study investigators in studies of diabetes:

______YES ______NO ______INITIALS

I give permission for my blood to be examined for inherited factors (genes) in the development of diabetes:

______YES ______NO ______INITIALS

I give permission for the results of studies on my blood to be used to develop improved methods for diagnosis, prevention, and treatment of diabetes:

______YES ______NO ______INITIALS

I give permission for the results of studies on my blood to be used for research about other health problems (for example, heart disease or related conditions such as high cholesterol):

______YES ______NO ______INITIALS

I give permission for my blood to be examined for inherited factors (genes) in the development of non-diabetic health problems (for example, heart disease or related conditions such as high cholesterol):

______YES ______NO ______INITIALS

A representative of the investigators associated with this study may contact me in the future to take part in more research.

______YES ______NO ______INITIALS

When I die, the specimens I have donated may still be used for the research purposes agreed to above.

______YES

______NO My specimens MUST be destroyed once you have been notified of my death.

______INITIALS

At the present time, we think that the tests for inherited factors (genes) will be for research purposes only, that is, they will not be useful to you or your health care provider. However, it is possible in the future that the DNA tests will find some results that will be useful to you, your family, and your health care provider. One result could be that there is a gene or genes in you and some members of your family that raises or lowers the risk for diabetes or heart disease. Such a result might take many years to be found. You may want to know the result. The study will assist you in identifying genetic counseling resources but will not pay for genetic counseling if it is necessary.

In the event that a useful result is found, I would like you to:

☐ Tell me of the results Initials:__________

☐ If I am not alive, tell the results to the relative(s) below: Initials:__________

Name of relative: ________________________________ Relationship______________

Name of relative: ________________________________ Relationship______________

Name of relative: ________________________________ Relationship______________

☐ Do not notify me or my family of the results Initials:__________

☐ Other: Initials:__________

____

____

15.6 Information and Amendment for the completion of the Masked Treatment Phase

NAME OF INSTITUTION

INFORMATION AND AMENDMENT FOR THE COMPLETION OF THE MASKED TREATMENT PHASE DIABETES PREVENTION PROGRAM (DPP)

INVESTIGATORS

Name of Principal Investigator
Title, Affiliation, Phone Number

Name of Co-Investigator(s)
Title, Affiliation, Phone Number

Name of Program Coordinator
Title, Affiliation, Phone Number

Name of Other Staff (*optional as per IRB*)
Title, Affiliation, Phone Number

24-Hour Emergency Telephone Number
(List phone number(s) here)

INVESTIGATOR'S STATEMENT:

PURPOSE AND BACKGROUND

This research study is called the Diabetes Prevention Program (DPP). You have been given the details concerning the Purpose, Background, Procedures, Risks and Benefits, and Costs of this study in a previous Consent Form. **This amendment is to provide you with information regarding the completion of the masked treatment phase of the DPP.**

The masked treatment phase of DPP will be completed earlier than planned. Masked treatment is when volunteers in the study don't know which medication they were taking. We have reported the main study results to you. This consent form will explain what we will ask you to do in the next few months, depending on the treatment group to which you were originally assigned.

You will be asked to schedule your individual debriefing visit from August through October. At your debriefing visit, you will be given your individual results collected during the

DPP. If you are assigned to the medication group, you will also be told if you were taking active medication or placebo.

PROCEDURES

You will be asked to continue visits until all study participants have completed their individual debriefing visit, anticipated to be sometime in late 2001.

Procedures to be carried out at one of your next visits include the following:

1. Blood flow and blood vessel wall thickness will be measured by ultrasound in your neck (carotid vessels). This will take about 30 minutes. If you did not have this test at the start of the study, you may not be asked to repeat it.

2. Questionnaires: You will be asked to answer questions about your participation in the DPP, and whether you have trouble with urination. These will take about 10 minutes to complete.

3. If you are in the medication group, a blood sample will be drawn for kidney and liver function tests every six months until your debriefing visit. The total blood drawn for these tests will be about 1 tablespoon. A repeat blood sample (about 1 tablespoon) might be necessary in some persons.

4. A urine sample will be collected.

Persons were randomly assigned to one of three (3) groups. The activities will be somewhat different, depending on your group.

1. <u>Lifestyle Participants</u>: You will be asked to continue to maintain your diet and exercise goals. You will meet with your lifestyle coach every other month. These visits will last about 30 minutes. Your lifestyle coach will contact you by telephone on the months that you are not meeting in person. Exercise classes will continue to be offered during this period.

2. <u>Medication Participants</u> (metformin or placebo): You will be asked to continue taking your masked study medication until your debriefing visit. In addition, you will be asked to continue your quarterly follow-up clinic visits. You may also be asked to take part in a "washout" study, which will be explained in a separate consent form.

You will return all study medication at your individual debriefing session. At your debriefing visit, you will be given your individual results, and you will be told if you were assigned to metformin or placebo.

a. <u>If you were taking metformin</u>: You will be asked to continue taking metformin, as you have done before, unless you have developed diabetes with a fasting blood sugar greater than 140 mg/dl. Metformin will not be "masked". You will know that you are taking metformin. This is called an "open-label" study. The possible risks of metformin have been explained to you in the treatment phase consent form you signed before.

b. <u>If you were taking placebo</u>: You will be told to stop taking placebo.

BENEFITS

During your participation in the DPP, you have received extensive medical examinations. We will continue to tell you if we find any problem. If you have not done so already, you will be asked to sign a written permission to release medical information to your doctor. As in the past, your health will be closely monitored during this final phase of the study. Problems such as diabetes might be found and treated sooner than if you were not in the study. This might improve your health.

At your debriefing visit, you will be given a summary of the results obtained during the time you participated in the study. These results may be beneficial to you in planning for your future health care.

RISKS, STRESS, AND DISCOMFORT

There are several possible risks of the study that were described to you previously in the Treatment Consent Form. There are no additional risks during this period. The carotid ultrasound study uses sound waves to measure the thickness of blood vessel walls. It is a commonly used diagnostic test that is non-invasive and painless.

Risks associated with drawing blood from your arm include pain, bruising, lightheadedness and on rare occasion, infection.

OTHER INFORMATION

(Institutional language as suggested by your IRB should be substituted for the following sections.)

Participation in this study is completely voluntary. You are free to take back your consent and stop taking part in this study at any time. You may ask any questions about the study at any time. Your current or future care will not be affected by your stopping the study.

Information that we get from you will be kept confidential to the extent allowed by law.

[Each center should incorporate a statement to address medical liability.]

If a test result shows that you should get medical care, you will be referred to your doctor. If you do not have a doctor, we will help you to see a doctor for medical care.

This study can be stopped or modified at any time.

If you have any questions about your rights as a research subject, you may call (IRB contact name) at (IRB phone number.)

PARTICIPANT'S STATEMENT:

The study described above has been explained to me. I understand that I am consenting to continue to participate in DPP during the end of the treatment phase. If I have any questions, I

know that I can contact one of the investigators listed on the first page. I have been given a copy of this consent form.

____

Participant's Signature Date

____

Person obtaining consent Date

____

Investigator's Signature Date

cc: Investigator
Participant

15.7 Information and Consent for a "Washout" Study of Metformin and Placebo

NAME OF INSTITUTION
INFORMATION AND CONSENT FOR A "WASHOUT" STUDY OF METFORMIN AND PLACEBO
DIABETES PREVENTION PROGRAM (DPP)

INVESTIGATORS

Name of Principal Investigator
Title, Affiliation, Phone Number

Name of Co-Investigator(s)
Title, Affiliation, Phone Number

Name of Program Coordinator
Title, Affiliation, Phone Number

24-Hour Emergency Telephone Number
(List phone number(s) here)

INVESTIGATOR'S STATEMENT:

PURPOSE AND BACKGROUND

You have been given the details concerning the Purpose, Background, Procedures, Risks and Benefits, and Costs of the Diabetes Prevention Program (DPP) in the DPP Treatment Phase Consent Form. You have also been told that metformin was found to prevent or delay diabetes more than placebo in the DPP. **This consent form is to provide you with information about a study to learn whether the positive treatment effects of metformin are due to a short-term treatment effect on high blood sugar, or due to longer lasting effects that delay the start of high blood sugar and diabetes.**

We do not know how metformin worked to prevent diabetes. During the DPP, all the oral glucose tolerance tests (OGTTs) were done while metformin was still in the body, since it was only stopped on the morning of the test. Thus, it is possible that metformin is simply treating the blood sugar to levels that appear to be normal, yet diabetes may already have developed. Alternatively, metformin may not be simply treating the blood sugar, but may have longer lasting effects through other routes, such as making the body's cells more efficient in using sugar, or lowering the amount of sugar that the liver produces. To determine which of these ways metformin works, we are asking you to stop the medication (metformin or placebo) 7 days before an OGTT, and remain off the medication until you are told of the results of the first test, or to have a second test, if the first test was not normal. This study is called a "washout" study, since the effects of metformin will be "washed out" or removed from your body at the end of the 7 day period.

We are not aware that a washout study has ever been done before with metformin, though similar washout studies have been done with other medications. Because such a study has not been done, we do not have any prior information to tell you about what the likely results will be. It is important to know whether metformin is acting by a short or longer-term effect. The answer to this question may affect how patients with IGT are treated in the future.

You are asked to participate in this study since you were assigned to the masked study medication (either metformin or placebo) during the DPP. You are now asked to take part in this washout study before you know which drug you were taking. After the results of your OGTT (and any repeat OGTT) are given to you, you will be told what drug (metformin or placebo) you were taking during the DPP. Thus, you are asked to stay masked to your individual DPP treatment until your washout test(s) are completed. If you are not currently taking your medication for any reason, you are still eligible for this study. Participation of all persons in the medication arm is important to avoid bias in understanding the results.

If you do not wish to participate in the washout study, you will be told during the next several weeks, which medication you took (metformin or placebo) as soon as appointments can be scheduled.

If you do participate in the washout study, you will also be told over the next several weeks, as soon as the OGTTs can be scheduled and your results completed. You will not need to wait until all participants have finished the washout study to get your results, only until you have finished the tests.

Regardless of your participation in the washout study, we will give you individual recommendations about taking metformin or not.

PROCEDURES

You have already been told of the major results of the DPP. As a participant in the medication arm, you are asked to:

1. Schedule an appointment for an OGTT.

2. Seven (7) days before the date of the OGTT, stop your DPP study medication and do not take any more until instructed to do so.

3. Complete the OGTT. This test will take about 2 and ½ hours. You will be asked to not eat or drink anything, except water, for 12 hours before your appointment. A blood sample, about 1 tablespoon, will be taken from your arm. You will then be asked to drink a glassful of flavored sugar water over 5 minutes. Another blood sample (about 1 tablespoon) will be taken from your arm at 30 and again at 120 minutes later.

4. Do not restart your medication until told to do so. Two (2) to (4) days after the OGTT, the results will be available. They will either be “normal”, or “repeat test needed”.

5. If a repeat test is needed, do not take your study medication, and schedule the next OGTT as soon as possible.

6. After finishing one or two OGTTs, schedule a visit to learn about your results during the DPP, the medication you were on, and the results of the washout OGTT tests.

BENEFITS

There is no direct benefit to you as a result of this study. However, by participating in this study, you will be helping DPP investigators to understand how metformin works in preventing or delaying diabetes. This information will help determine how metformin will be used in the future.

RISKS, STRESS, AND DISCOMFORT

This research could affect your medical care. There is a risk that by stopping the medication you will be "diagnosed" with diabetes by the OGTT testing. The diagnosis of diabetes may increase your insurance rates, make it difficult or impossible to get health or life insurance, and cause you and your family emotional distress. It may also increase your personal expenses for treatment of diabetes. The consequences are the same as those in the DPP from repeated OGTT testing for diabetes.

Risks associated with drawing blood from your arm include pain, bruising, lightheadedness and, on rare occasion, infection. To decrease the number of intravenous punctures performed we may place an intravenous catheter to obtain the blood samples during the OGTT.

(Institutional language as suggested by your IRB should be substituted for the following sections.)

CONFIDENTIALITY

Your investigator and the NIH will treat your identity with professional standards of confidentiality to the extent the law allows.

OTHER

[Insert institutional language as needed for other sections]

Source of funding

All funding for this study will be provided by the National Institutes of Health (NIH).

Costs to Participant

There is no cost to you for participating in this study. There will be no charge for procedures required by the study, which will be paid for by NIH.

Participation in the washout study is entirely voluntary. You are free to take back your consent at any time and you may request to stop the tests, and to be told of your medication assignment. You stopping the study will not affect your current or future care.

[Each center should incorporate a statement to address medical liability.]

If the results of the OGTT test(s) shows that you have developed diabetes, you will be referred to your doctor, as in the DPP. If you do not have a doctor, we will help you to see a doctor for medical care. You will also get education about diabetes and how to test your blood sugar levels. You will be given a blood sugar test meter free of charge.

This study can be stopped or modified at any time.

If you have any questions about your rights as a research subject, you may call (IRB contact name) at (IRB phone number.), or the investigators listed on this consent form.

PARTICIPANT'S STATEMENT:

The study described above has been explained to me. I understand that I am consenting to take part in the washout study to find out if metformin is treating high blood sugar or preventing high blood sugar from starting. I also agree to remain masked to whether I am taking metformin or placebo until my tests are finished and the results are known. If I have any questions, I know that I can contact one of the investigators listed on the first page. I have received a copy of this consent form.

____

Participant's Signature Date

____

Person explaining study signature Date

____

Investigator’s Signature Date

cc: Investigator
Participant

15.8 Consent Forms for Bridge Period

IRB Templates - Attached separately.

NAME OF INSTITUTION

INFORMATION AND ADDENDUM FOR THE COMPLETION OF THE TREATMENT PHASE OF THE DIABETES PREVENTION PROGRAM (DPP)

INVESTIGATORS

Name of Principal Investigator
Title, Affiliation, Phone Number

Name of Co-Investigator(s)
Title, Affiliation, Phone Number

Name of Program Coordinator
Title, Affiliation, Phone Number

Name of Other Staff (optional as per IRB)
Title, Affiliation, Phone Number

24-Hour Emergency Telephone Number
(List phone number(s) here)

INVESTIGATOR'S STATEMENT:

PURPOSE AND BACKGROUND

This research study is called the Diabetes Prevention Program (DPP). You have been given the details concerning the Purpose and Background of this study in a previous Consent Form. **This addendum is to provide you with information regarding the completion of the treatment phase of the DPP and the start of Group Lifestyle Sessions.**

Because of positive results, the masked phase of the DPP ended early. In August 2001, the results were announced, showing that the risk of Type 2 diabetes was reduced by 58% in the lifestyle arm of the study and 31% in the metformin arm. We are now completing the treatment phase of the DPP. This period will begin in January 2002. Group Lifestyle Sessions will be offered to all DPP participants during this time. You are invited to take part in these sessions as well as to continue your participation in the ongoing DPP interventions.

BENEFITS

During your participation in the DPP, you will continue to receive medical testing. We will continue to tell you if we find any problem. If you have not done so already, you will be asked to sign a written permission to release medical information to your doctor. As in the past, your health will be closely monitored during this final phase of the study. Problems such as diabetes might be found and treated sooner than if you were not in the study. This might improve your health.

Intensive lifestyle intervention was shown to be very effective in reducing the onset of diabetes. Participation in a group lifestyle intervention may help to reduce your risk of developing diabetes.

PROCEDURES

All study participants will be invited to participate in a 16-lesson lifestyle change program with the same goals and similar in content to the curriculum that was included in the DPP intensive lifestyle intervention. The program will be offered in two parts consisting of 8 lessons each and will occur in a group class rather than an individual format. A trained professional will lead the group meetings. Each lesson will be offered several different times per week in order to allow you to attend at a time that is convenient for you. Classes will begin in January and be completed by mid-2002 and will last 1-2 hours per session.

The exercise and diet goals of the program are walking 2 1/2 hours (150 minutes) per week (or similar activity) and using healthy eating habits to lose 7% of your body weight. You will be weighed at the classes. You will also be given materials to help track your fat and calorie intake and your activity. After completion of the first 8 lessons, you will be invited to sign up for the second half of the program (the last 8 lessons). The classes will help you learn more about making healthy food choices and increasing activity. They will also allow you to talk about concerns you have in changing your lifestyle habits. If you do not attend a lifestyle class, handouts from that lesson will be mailed to you.

Although the group lifestyle sessions will be offered to ALL DPP participants, you may choose not to take part in these sessions and still remain a participant in the DPP. In addition to the group lifestyle sessions, you will be asked to do the following:

Lifestyle Participants: You will be asked to continue to maintain your diet and exercise goals throughout the remainder of the study or until study investigators decide the lifestyle intervention can no longer be offered. This could occur anytime after February of 2002 You will meet with your lifestyle coach at least every other month through the end of June 2002. These visits will last about 30 minutes. Your lifestyle coach will contact you by telephone on the months that you are not meeting in person. You will be asked to attend your regularly scheduled mid-year and annual visits, and you will be contacted quarterly by telephone or for a clinic visit.

Medication Participants: (metformin) You will be asked to continue taking metformin open-label (unmasked) until the study investigators decide it can no longer be offered. This could occur anytime after February of 2002. You will be asked to return all unused study medication when you stop taking it, or at the end of June 2002, whichever occurs first. In addition, you will be asked to attend your regularly scheduled mid-year and annual visits, and you will be contacted

quarterly by telephone or for a clinic visit. Some people may be asked to attend one extra visit to collect measurements that were deferred during the washout study.

Placebo Group: You will be asked to attend your regularly scheduled mid-year and annual visits, and you will be contacted quarterly by telephone or for a clinic visit. You will no longer be asked to have safety blood testing (kidney, liver function and complete blood count). Some people may be asked to attend one extra visit to collect measurements that were deferred during the washout study.

In addition, participants who have developed diabetes during the study will be asked to attend an interim visit for a blood glucose check between the annual and mid-year visits.

Procedures to be carried out at the clinic visits:

Mid-year visit: (Approximately 45 Minutes)

1. Blood pressure will be measured in your arm. This will take about 10 minutes

2. Body Measurement: Your weight will be measured.

3. A blood sample (about 1 tablespoon) for fasting glucose will be taken from your arm. You will be asked not to eat or drink anything, except water for 12 hours before your appointment. If you are in the metformin group, an additional sample will be drawn for kidney and liver function tests. A repeat blood sample (about 1 tablespoon) may be necessary for some persons.

4. You will be asked to complete a questionnaire. This will take about 10 minutes.

Annual Visit: **(Approximately 1.5-3.5 hours)**

1. **[Clinics should insert whether they will do a physical examination - it is now optional]**

   [if yes - insert: A physical examination will be completed. This will take about ½ hour.]

2. An electrocardiogram (ECG) will be performed. This will take about ½ hour.

3. Blood pressure will be measured in your arm, and may be measured in your ankle as well. This will take about 10-30 minutes.

4. There are several questionnaires that you will be asked to complete. You will be asked questions about your health, physical activity, diet, and feelings. These will take about 90 minutes to complete.

5. Body measurements: Your weight and waist and hip size will be measured. This will take about 10 minutes.

6. Oral glucose test: This test will take about 2 and ½ hours. You will be asked to not eat or drink anything, except water, for 12 hours before your appointment. A blood sample will be taken from your arm. You will then be asked to drink a glassful of flavored sugar water over 5 minutes. Another blood sample) will be taken from your arm at 30 and again at 120 minutes. The total amount of blood drawn for this test is approximately 1 tablespoon. A repeat oral glucose test may be necessary for some persons. People with diabetes will not be asked to complete the oral glucose test, but will have a fasting blood glucose drawn.

7. Additional blood samples will be taken from your arm at the same time that you are having blood drawn for the oral glucose test, for lipids (blood fats), and other blood tests related to diabetes and heart disease. If you are in the metformin group, an additional sample will be drawn for kidney, liver function tests, and complete blood count. The total blood drawn for all these tests will be 4-5 tablespoons. A repeat blood sample (about 1 tablespoon) might be necessary in some persons.

Interim Blood Glucose Check for Participants with Diabetes (Approximately 30 minutes)

1. A blood sample (about 1 tablespoon) for fasting glucose will be taken from your arm. You will be asked not to eat or drink anything, except water for 12 hours before your appointment.

RISKS, STRESS, AND DISCOMFORT

There are several possible risks of the study that were described to you previously in the Treatment Consent Form. There are no additional risks during this end of study period. [If local IRB requires, insert all risks from metformin from treatment phase consent form].

Risks associated with exercise include fatigue, muscle soreness, and injury such as sprained ankles or pulled muscles. Risks are reduced by proper warm-up and cool-down periods. There may be additional risk of heart problems for those who have a chronic disease or experience symptoms with exercise, although this risk is extremely minimal considering the intensity of the recommended exercise, i.e., walking. You will need to contact your primary care provider for his/her permission for you to take part in the physical activity segment of the healthy lifestyle program. If you do not receive signed permission from your primary care provider, you will not be able to take part in the exercise segment of the program.

Risks associated with drawing blood from your arm include pain, bruising, lightheadedness and on rare occasion, infection.

CONFIDENTIALITY

Information that we receive from you will be kept confidential to the extent allowed by law. Information we collect about you will be put into a research record that will be sent to a central data site. A code number and initials identify your records. The link between the code and your name is stored in a secure location at the insert your institution name. Thus, your central research record will not be directly identified with your name. Anonymous information may be released to a DPP investigator (or other investigator authorized by the DPP) only after determination of the scientific usefulness of a proposed study. [Insert appropriate language as determined by your IRB].

OTHER INFORMATION

As in the past, some of the test results will not be made known to you or the clinic staff or your own doctor. This is known as "masking". The only blood tests that will be masked in some cases will be the results of the oral glucose tolerance test or fasting glucose test, until it is confirmed to be normal or shows that diabetes has developed, when it will be unmasked.
(Institutional language as suggested by your IRB should be substituted for the following sections.)

Participation in this study is completely voluntary. You are free to take back your consent and stop taking part in this study at any time. You may ask any questions about the study at any time. Your current or future care will not be affected by your stopping the study

[Each center should incorporate a statement to address medical liability.]

If a test result shows that you should get medical care, you will be referred to your doctor. If you do not have a doctor, we will help you to see a doctor for medical care.

This study can be stopped or modified at any time.

If you have any questions about your rights as a research subject, you may call (IRB contact name) at (IRB phone number.)

PARTICIPANT'S STATEMENT:

The study described above has been explained to me. I understand that I am consenting to continue to participate in the last phase of the DPP study period. If I have any questions, I know that I can contact one of the investigators listed on the first page.

____
Participant's Signature Time Date

____
Investigator's Signature Date

____
Person obtaining consent Date

cc: Investigator
Participant
